# Prioritizing Colombian plant genetic resources for investment in research using indicators about the geographic origin, vulnerability status, economic benefits, and food security importance

**DOI:** 10.1007/s10531-023-02599-7

**Published:** 2023-05-19

**Authors:** I. Cerón-Souza, D. Delgadillo-Duran, S. M. Polo-Murcia, Z. X. Sarmiento-Naizaque, P. H. Reyes-Herrera

**Affiliations:** CI Tibaitatá, Corporación Colombiana de Investigación Agropecuaria, AGROSAVIA, Km 14 via Mosquera, Bogotá, Colombia

**Keywords:** Crop species, Ex situ conservation, Fuzzy logic, Genebanks, Germplasm banks, Information imputation

## Abstract

Germplasm banks are the most significant repository for plant genetic resources for food and agriculture (PGRFA) worldwide. Despite their strategic importance, national germplasm banks of tropical megadiverse developing countries such as Colombia have extremely limited funds. Therefore, making strategic decisions about research investment is essential. Here, we designed a data-driven approach to build an index that sorts Colombian PGRFA into three groups: high, medium, or low priority, based on four pillars of information from open-access databases and aligned with the sustainable goals of no poverty and zero hunger: Geographic origin, vulnerability status, economic benefits, and food security importance. We analyzed 345 PGRFA using the index, separating them into two groups, 275 already conserved in the Colombian germplasm bank (BGVCOL group) and 70 not currently conserved in the BGVCOL (NCB group). We used fuzzy logic to classify each PGRFA by each pillar and integrate it to obtain a priority index. Missing data for native crops were frequent in the BGVCOL group. Therefore we adopted an imputation strategy to fill the gaps and calculated the uncertainty. After applying the index, PGRFA with higher priority were 24 (8.72%) from the BGVCOL (i.e., 15 potatoes, three tomatoes, two tree tomatoes, pineapple, cocoa, papaya, and yacon) and one from NCB (i.e., coffee). We concluded that this methodology successfully prioritized PGRFA in Colombia and shows the big holes of knowledge for future research and alternatives to improve this index. The versatility of this methodology could be helpful in other genebanks with budget limitations for research investment.

**Supplementary Information:**

The online version contains supplementary material available at 10.1007/s10531-023-02599-7.

## Introduction

Biodiversity hotspots are mainly tropical and subtropical zones (Myers et al. 2000). These areas also match 28% of the centers of crop domestication (Gepts 2006). From these areas, Latin American countries are essential. They include the Mesoamerican and Andean centers of diversity defined by Vavilov, where several crop species are critical for worldwide food security, such as maize, potato, and beans (León-Lobos et al. 2012). This favorable situation should imply the sustainable use of biodiversity for goods and services among Latin American countries. However, this is still not the case. In 2015, hunger and malnutrition affected around 34 million people in Latin America and the Caribbean, contributing to 5.5% worldwide (FAO 2015). Also, one in five people lives in chronic poverty within this region (Vakis et al. 2016). This situation is getting worse because of the COVID-19 pandemic. The lockdown closed the food programs for poor people in Latin America and the Caribbean, affecting mainly women, children, and immigrants (Swinnen and McDermott 2020; FAO 2020). Also, the lockdown impacted the demand and supply of food, both associated with national food security (López-Feldman et al. 2020; Siche 2020; Swinnen and McDermott 2020).

Before the pandemic, Latin American countries (except Brazil) invested less than 1% of GDP in scientific research, corresponding to less than the lower-middle-income economies worldwide (Lemarchand 2015). Nevertheless, the COVID-19 pandemic has exemplified health and agriculture’s importance in strengthening food security (Pray et al. 2021; Becerra-Posada et al. 2021). Hence, the current global context is an opportunity to reexamine the extreme importance of Latin American countries in supporting the well-planned collection, ex situ conservation, and research of plant genetic resources for food and agriculture (PGRFA). Ultimately, this investment means insurance to prevent the social cost of biodiversity loss (Perrings 1995; Gepts 2006).

The PGRFA includes all the plants with actual or potential value for agricultural production that breeders use to develop new crop varieties. Those materials cover crops, wild crop relatives, old cultivars, landraces, and traditional cultivars obtained by farmers using natural or artificial selection. Therefore, there is the basis of humankind’s survival because it generates food, fuel, and fibers (Ho 2010). Currently, 68.7% of national food supplies are foreign crops, and no country is enterely self-sufficient (Khoury et al. 2016). This interdependence among countries results from human migration history that has promoted over centuries the interchange of landraces, and varieties (Ho 2010). Thus, the economic benefits of the discovery and use of PGRFA vastly exceed the investment in searching for and conserving them within and among countries (Rubenstein et al. 2011).

Unfortunately, PGRFA is especially susceptible to the decline of genetic variation (i.e., genetic erosion). The main reason is the frequent replacement worldwide of local varieties by modern varieties and intensive agriculture that promotes genetic uniformity, unsustainable agriculture, and neglected local PGRFA. Moreover, new pests, diseases, and environmental factors such as global warming, urbanization, and land destruction are other main threats to PGRFA (Khoury et al. 2021). Many countries combine strategies of conservation in situ (in their natural habitat) and ex situ (outside their natural habitat) for PGRFA. The ex situ method implies the long-term conservation of PGRFA, mainly in the germplasm banks, also known as genebanks. However, since establishing the germplasm banks across different countries, the main concern has been scarce funds to maintain long-term research programs, appropriate facilities for long-term conservation of PGRFA, and specialized staff (Rubenstein et al. 2011). Currently, the United Nations generates 17 sustainable development goals (SDGs), known as global goals focused on improving peace and prosperity for humankind by 2030 (UNDP 2021). From these goals, goal 1: no poverty, and goal 2: zero hunger, represent opportunities to align the mission of national germplasm banks with stakeholders to increase the financial support for PGRFA conservation and research across different countries, including Colombia (Mba et al. 2020).

Colombia is classified as a “megadiverse” country hosting around 10% of global biodiversity (Clerici et al. 2019). Since 1995, Colombia has been part of the Convention of Biological Diversity (CBD), a global initiative that promotes the protection of life diversity and its sustainable use. One of the most powerful frameworks for action within the CBD is the investment in ex situ conservation. In Colombia, the National Plant Germplasm Bank (BGVCOL) is part of this ex situ public effort for conservation which started in 1994. However, the PGRFA collection across the country started between the 1920s and 1950s as part of a national breeding program supported by the Rockefeller Foundation and the US technical cooperation in Latin America, generating the first seed banks (Valencia et al. 2010). In 1996 the government promoted the national germplasm bank system with an annual budget for the conservation of all PGRFA conserved in the BGVCOL besides the animal and microorganism national banks. Moreover, since 2018, the Agriculture and Rural Development Minister has delegated the administrative function of national germplasm banks to Agrosavia (*Corporación Colombiana de Investigación Agropecuaria*), including a material delivery agreement (MTA) for acquiring and delivering PGRFA according to the national and Agrosavia’s internal policies under the CBD. However, some details about this policy could change midterm because the national congress recently ratified and approved the entrance to the ITPGRFA (International Treaty on Plant Genetic Resources for Food and Agriculture) and now is in the process of legal clearance by the Colombian Constitutional Court.

Currently, the BGVCOL maintains more than 30,000 different accessions from 275 species and 109 groups of taxa conserved in three systems: seeds, field, and in vitro (Valencia et al. 2010). However, despite the dimension of the BGVCOL, there is no system to prioritize research investment for the PGRFA already conserved within the BGVCOL, and a criterium to determine which external PGRFA not conserved in the BGVCOL should start to be part of an ex situ conservation plan. The absence of information for biodiversity applies to all PGRFA, and it is necessary to evaluate updated inventories of Colombian diversity, especially for wild plants and orphan crops; our rationale is that the importance of PGRFA could change under a current criteria revision and become a priority for the national ex situ conservation effort.

**Fig. 1:**
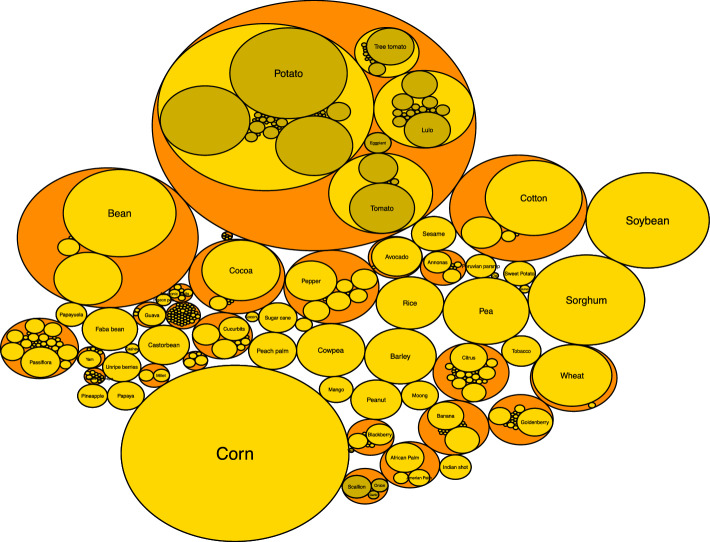
Circular plot representing the 275 plant genetic resources for food and agriculture (PGRFA) currently conserved in the National Plant Germplasm Bank (i.e., BGVCOL group). Each circle represents a crop species using its common name, and the radius’s size is according to the number of accessions conserved

**Fig. 2:**
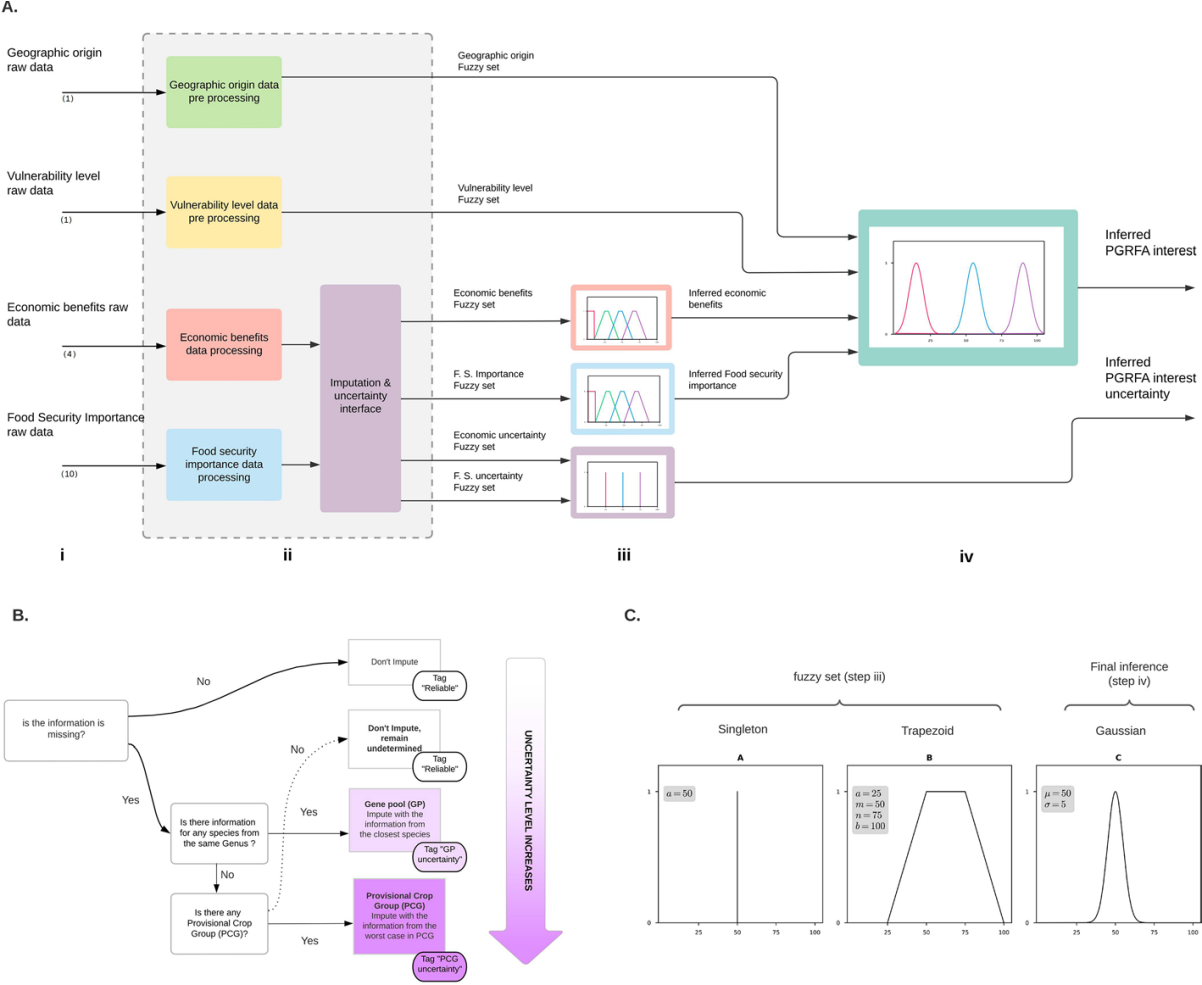
**A** The general strategy for analyzing raw data of 345 Plant Genetic Resources for Food and Agriculture (PGRFA) from Colombia. Two hundred seventy-five (275) are part of the National Plant Germplasm Bank (i.e., BGVCOL group), and 70 are essential for the Colombia government but not currently conserved in the BGVCOL (i.e., NCB group). (i) The analysis includes four data-driving pillars with different variables (the number in parenthesis). (ii) The preprocessing of the raw data for each variable within each pillar: geographic origin (green), vulnerability (yellow), economic importance (red), and food security importance (blue). Both economic importance and food security importance had several holes of information for some PGRFA. In those cases, the preprocessing included values from the phylogenetically closest PGRFA to impute and, therefore, the uncertainty calculation (purple box). (iii) The construction of the variables’ membership function (fuzzy sets) is based on either singleton or trapezoid. The geographic origin and vulnerability involved a unique qualitative variable that generated a singleton fuzzy logic function. Economic and food security importance had four and ten quantitative variables, generating trapezoid fuzzy logic functions. (iv) The final inference for the prioritization list and uncertainty level is based on Gaussian and singleton fuzzy logic functions. **B** The detail of imputation strategy with paths and outcomes (purple box in **A**). According to the imputation path taken, the information received a tag to track the level of uncertainty (i.e., reliable, GP uncertainty, or PCG uncertainty). **C** An example for the membership functions (fuzzy sets) used in steps iii and iv from A. The singleton (for categorical data) used *a* = 50, and SD = 0.001. The trapezoid (for numerical data) used 25, 50, 75, and 100 as the limits of the shape at the bottom left *a*, top left *m*, top right *n*, and bottom right *b*, respectively. At the end of the process, the Gaussian membership function (for inferring the PGRFA interest) used $\mu$ = 50 and $\sigma$ = 5. The three functions had a membership grade equal to one

Accordingly, this study focused on creating a prioritizing index to fill this gap. This index used four pillars (i.e., criteria of information) to construct a data-driven approach based on fuzzy logic (Jones and Cheung 2018) for well-planned research investment in the future in Colombia in two PGRFA groups: the currently conserved in the BGVCOL and the externals (Figs. 1, 2).

The pillars we proposed are geographic origin, vulnerability status, economic benefits, and food security importance. The endemism level and the vulnerability status have usually been considered ranking species for ex situ conservation (Barazani et al. 2008; Farnsworth et al. 2006; Jiménez-Alfaro et al. 2010). In contrast, as far as we know, the economic benefits and the importance of food security are not usually included as indicators for ranking PGRFA and making research investment decisions. In this study, we defined the economic benefits as the economic profit for the country to cultivate a determined PGRFA, and we used four macroeconomic variables to comprehend this pillar. Moreover, we defined the importance of food security as to how nutritious an edible PGRFA is to fulfill the food security needs of Colombians, especially infants from rural families. Also, we used four variables to analyze them based on micronutrients (i.e., calcium, iron, zinc, and energy).

We assessed these four pillars together for two main reasons. First, they could evaluate critical information associated with the SDGs, such as no poverty and zero hunger, as part of the ex situ conservation mission of the BGVCOL (UNDP 2021). Second, Colombia currently has particular challenges that need urgent attention in national agriculture research. They included the high risk of agriculture production in the face of global warming (MADR 2021), the peace agreement signed in 2016 with a big focus on rural development (i.e., comprehensive rural reform) but with delays in their implementation (Final Agreement 2016), and a severe population inequity exacerbated by the COVID-19 pandemic affecting more rural zones (DANE 2021). Thus, our rationale was that by identifying native, vulnerable, profit, and nutritive PGRFA, we align the long-term ex situ conservation mission of the BGVCOL with high-impact research for the country.

Using the prioritizing index based on these four pillars proposed here, we found 24 PGRFA from 275 with high research priority conserved in the BGVCOL and only one high-prioritized PGRFA from 70 externals. This data-driven approach developed in this study is adaptable to include more PGRFA, pillars, and variables within each pillar in the future. Moreover, although this study focused on Colombia, other countries and institutions could also adopt this tool to rank and prioritize the investment in ex situ conservation of their PGRFA.

## Methods

This study focused on generating a prioritizing index that sorted 345 national PGRFA from two different data sets. The first set corresponded to 275 currently conserved ex situ in the BGVCOL (i.e., BGVCOL group) (Fig. 1). The second set corresponds to 70 PGRFA never conserved in the BGVCOL (i.e., NCB group). These externals to the BGVCOL represent a small portion of all the PGRFA Colombia has as a megadiverse country (Gori et al. 2022). However, we selected them because they have appeared listed in several national agencies since 2013 because of their cultural, economic, and nutritional value, thus: 49 from the Ministry of Agriculture and Rural Development and EVA (Evaluaciones Agropecuarias Municipales) (MADR 2017), 14 from ICBF (Instituto Colombiano de Bienestar Familiar) (2018), five from the Ministry of Culture (MinCultura 2012) and two from PNSAN (National Plan for Food and Nutritional Security) (2012).

We created a data-driven methodology based on four pillars (i.e., geographic origin, vulnerability status, economic benefits, and food security importance) to sort and rank this 345 PGRFA from the most prioritizing to the less prioritizing for investment in research. We combined the four pillars’ data using fuzzy logic. The fuzzy logic discipline is the opposite of pure probabilistic reasoning, where everything in a universe is true or false. Instead, the fuzzy logic departs from the dichotomy principle and assumes that everything is a question of degree where neither is true nor false (Pedrycz and Gomide 2007). Thus, the membership function allows assigning different memberships to each contributing information element in a specific data universe (Zadeh 1965).

The fuzzy logic methodology employed here had several consecutive steps (Fig. 2). The first step, commonly called preprocessing, consisted of the normalization and scaling of raw numerical data. Then, we assigned a tag and a fuzzy membership function for every variable, called fuzzification. Geographic origin and vulnerability status corresponded to a unique variable for each one. In contrast, economic benefits and food security importance result from a combination of four variables for each pillar, as multiple factors influence them (Fig. 2A). Although there may be dependencies between variables within a given pillar, the four pillars remain separate and distinct in determining the priority of PGRFA.

There was no information for all variables for each one PGRFA. Therefore, in the BGVCOL group, we imputed the information if we found within the BGVCOL a species from the same genus or same FAO category and calculated a level of uncertainty for each species (Fig. 2B). However, for the NCB group, we did not use an imputation strategy because as externals to the BGVCOL group, we did not have in all the cases a straight-forward strategy to impute.

Next, after obtaining the variables directly or imputed, each variable by each pillar corresponds to a data universe X:[0,100] containing information elements that could be discrete or continuous. If the information was discrete, we used singleton membership functions. In contrast, if the information was continuous, we used either a trapezoid (used for input membership functions) or a Gaussian function (output membership function) (Table 1). We defined the membership function using the R-package Sets 1.0-18 (Meyer and Hornik 2009) under R Studio ver. 3.6. (Table 1, Fig. 2C). Finally, we used all the inferred information for each pillar to determine the priority index and the uncertainty level for each species from the 345 PGRFA. The following sections explain the rationale of each step in all detail.

**Table 1:** The variables evaluated in this study consider four pillars of information: geographic origin, vulnerability, economic importance, and food security importance

Pillar	Variable	Type	Number of classes	Classes (priority level)	Function	The parameters of the membership functions
Geographic origin	Geographic origin	Singleton	3	Distant origin (low priority)		$a = 0$
				Closest origin (middle priority)		$a = 45$
				Local origin (high priority)		$a = 70$
Vulnerability level	Vulnerability level	Singleton	3	Not evaluated		$a = 0$
				Minor concern (low priority)		$a = 30$
				Threatened (high priority)		$a = 70$
Economic Importance	Lafay Index	Singleton, trapezoid	4	Undetermined		$a = 0$
				Low (Low priority)		$a = 1, \; m = 5.2,$ $n = 10.4, \; b = 17.68$
				Medium (middle priority)		$a = 10.4, \; m = 20.8,$ $n = 20.8, \; b = 31.12$
				High (high priority)		$a = 20.8, \; m = 31.2,$ $n = 100, \; b = 100$
	Yield	Singleton, trapezoid	4	Undetermined		$a = 0$
				Low (low priority)		$a = 4.9, \; m = 5,$ $n = 28.2, \; b = 37.6$
				Medium (middle priority)		$a = 28.2, \; m = 37.6,$ $n = 54, \; b =65.2$
				High (high priority)		$a = 54, \; m = 65.2,$ $n = 100, \; b = 100$
	Income	Singleton, trapezoid	4	Undetermined		$a = 0$
				Low (low priority)		$a = 4.9, \; m = 5,$ $n = 28.2, \; b = 37.6$
				Medium (middle priority)		$a = 28.2, m = 37.6,$ $n = 54, b = 65.2$
				High (high priority)		$a = 54, \; m = 65.2,$ $n = 105, \; b = 110$
	Municipality coverage	Singleton, trapezoid	4	Undetermined		$a = 0$
				Narrow (low priority)		$a = 0.99, \; m = 1,$ $n = 15, b = 30$
				Medium (medium priority)		$a = 20, m = 25,$ $n = 50, b = 60$
				Large (high priority)		$a = 55, m = 60,$ $n = 101, b = 105$
	Species economic importance (output)	Trapezoid	4	Undetermined		$a = 0, m = 0.01,$ $n = 9.9, \; b = 10$
				Low (low priority)		$a = 10, m = 25,$ $n = 30, b = 45$
				Medium (medium priority)		$a = 30, m = 45,$ $n = 50, b = 65$
				High (high priority)		$a = 50, \; m = 65,$ $n = 70, \; b = 85$
Food security importance	Government priority list	Singleton	2	No Included (low priority)		$a = 20$
				Included (high priority)		$a = 60$
	Traditional consumption	Singleton, trapezoid	4	Undetermined narrow use (low priority) medium use (middle priority) large use (high priority)		$a = 0$ $a = 0.11, \; m = 1,$ $n = 1.1, \; b = 1.25$ $a = 1.05, \; m = 1.1,$ $n = 1.15, \; b = 1.35$ $a = 1.35, \; m = 19,$ $n = 100, \; b = 100$
	Nutritional contribution	Trapezoid	4	Undetermined		$a =0, \; m = 0.01$ $n = 9.99, \; b = 10$
				Low (low priority)		$a = 10, \; m = 26,$ $n = 30, \; b = 45$
				Medium (middle priority)		$a = 30, \; m = 45,$ $n = 50, \; b = 65$
				High (high priority)		$a = 50, \; m = 65,$ $n = 100, \; b = 100$
	Affordability based on ts nutrients	Trapezoid	4	Undetermined		$a =0, \; m = 0.01$ $n = 9.99, \; b = 10$
				Low (high priority)		$a = 10, \; m = 26,$ $n = 30, \; b = 45$
				Medium (middle priority)		$a = 30, \; m = 45,$ $n = 50, \; b = 65$
				High (low priority)		$a = 50, \; m = 65,$ $n = 100, \; b = 100$
	Species importance for food security (output)	Trapezoid	4	Undetermined		$a = 0, \; m = 0.01,$ $n = 9.99, \; b = 10$
				Low (low priority)		$a = 10, \; m = 25,$ $n = 30, \; b = 45$
				Medium (middle priority)		$a = 30, \; m = 45,$ $n = 50, \; b = 65$
				High (high priority)		$a = 50, \; m = 65,$ $n = 70, \; b = 85$
Prioritizing index		Gaussian	3	Low (low priority)		$\mu =15, \;\sigma =5$
				Medium (middle priority)		$\mu =55, \;\sigma =5$
				High (high priority)		$\mu =90, \;\sigma =5$

Each variable within the pillar specifies the type of data (i.e., singleton for categorical data and trapezoid or gaussian for continuous data), the number of classes for each type (i.e., fuzzy sets), the name of each class with its priority in parenthesis, the fuzzy logic function, and the parameters for each membership function. The undetermined class means unavailable or uncertain data

### Geographic origin

According to their geographic origin, we classified the 345 PGRFA following the classification of regions of diversity for crops in the world (Khoury et al. 2016) and the Powo database (2021) that recognizes 26 areas globally. We grouped the 26 regions into three classes (fuzzy sets): local, close, and distant. The local label included the Andes and Tropical South America because both contained Colombia. The close title corresponded to the Caribbean, Central America and Mexico, and temperate South America because they are neighboring areas around the two locals. Finally, we grouped the 22 remaining regions within the distant label. They were Australia, Indian Ocean Islands, Central Africa, East Africa, Southern Africa, East Africa, West Africa, North America (Canada and USA), Asia, West Asia, South Asia, Southeast Asia, Central Asia, East Asia, Southeast Asia, Europe, Southeast Europe, South Mediterranean, Northeast Europe, Southwest Europe, Northwest Europe, and East Mediterranean. Thus, the membership functions for this pillar are of the singleton type with three priority ranking categories thus, high for local, middle for close, and low for distant (Table 1).

### Vulnerability status

We classified the 345 PGRFA vulnerability status into five categories: endangered, vulnerable, near threatened, minor concern, and not evaluated according to the Colombian national catalog (Universidad Nacional de Colombia 2019). We updated the classification for three local endangered species *Elaeis oleifera*, *Bactris gasipaes*, and *Passiflora jardinesis*, using the resolution 1912 of 2017 from Colombia’s Ministry of Environment and Sustainable Development. These lists did not contain all the species considered in this study. Consequently, we consulted the Botanical Garden for International Conservation International (2019) and Red List platforms (2019). Both databases separated the species into six categories instead of five: critically endangered, endangered, vulnerable, near threatened, minor concern, and no evaluated. Hence, to analyze the 345 PGRFA, we merged the national and the international vulnerability categories into three: not evaluated, minor concern, and threatened.

The not evaluated category included all PGRFA without national or international information across the databases consulted. The minor concern category combined the categories near threatened and least concern from national and international databases. Finally, the threatened category involved both endangered and vulnerable classifications from the national database and three classes (i.e., critically endangered, endangered, and vulnerable) from the international databases. Based on the final merged categories of vulnerability, the membership function for this pillar resulted from the singleton type and with three levels of ranking categories threatened with high priority, minor concern with low priority, and not evaluated (Table 1).

### Economic benefits

We identified the Colombian agricultural production trends for each 345 PGRFA using four variables: Income, Lafay index, municipality coverage, and yield. There may be dependencies between variables within the economic benefits pillar, as multiple factors may influence each variable. We used the information of each variable separately to organize each PGRFA into categories within the BGVCOL and NCB groups. However, we only ranked the PGRFA after integrating four variables into a single economic benefits index based on fuzzy logic (Table 1 and Fig. 1). For obtaining the information on each variable, we used three databases: (1) the Information and Communication Network of the Minister of Agriculture and Rural Development (MADR) for data about the harvested area (ha), production (t), and yield (t ha$^{-1}$) of each PGRFA separated by municipalities for ten years from 2007 to 2017 (MADR 2017), (2) the consumer prices for each PGRFA from the Agricultural Sector Price Information System (DANE) in 2019 (DANE 2019), (3) the import and export information registered in the LegisComex database from 2019 (LegisComex 2019).

#### Income

Increasing farmers’ productivity and income is a fundamental aspect of reducing rural poverty and ensuring food security (World Bank 2007; Habtemariam et al. 2019). Here, we chose the income per unit area as a proxy for each PGRFA’ economic efficiency at the aggregate level within Colombia. We expressed this relation as ${Inc_{i}}=({P_{i}} \times {P_{r_{i}}})/{A_{i}}$. The $Inc_i$ represented the income per hectare of species *i* (units in USD ha$^{-1}$, where the last available data is from 2017). The $P_i$ indicated the sales price of species *i* expressed in USD t$^{-1}$). The $P_{r_i}$ showed the annual production of species *i* described in tons. Finally, the $A_i$ represented the harvested area of species *i* expressed in hectares.

We labeled the income data as undetermined, low, medium, and high. The undetermined label referred to the absent or unavailable information and had a zero value. The other three tags (i.e., low, medium, high) corresponded to continuous available data with values between 1 and 50 (thousand USD ha$^{-1}$). The distribution of this data had a large range, and therefore we preprocessed using a logarithm $(Log (Inc_i ))$. Based on $Log (Inc_i)$, we calculated the centroid of three clusters (i.e., K = 3) and assigned the individuals with data to one of the three clusters by using k-means [R package Ckmeans.1d.dp (Wang and Song 2011)]. The low, medium and high labels had trapezoid membership functions for the PGRFA (see Table 1 for details about the function parameters).

#### Lafay index

We measured the Lafay index (*Li*) for the contribution to the trade balance thus: $Li=Pdi/(Pdi+Mi-Xi)$, where *Pdi* is the annual production of the species *i* (t), *Mi* is the yearly imports for the species *i* (t), and *Xi* is the annual exports for the species *i* (t) (Lafay 1992). The result of this equation is a quotient between the production of a crop and its apparent consumption (i.e., production plus import minus export) in a year. If this value is higher than one, the country is a net exporter of the crop, and the higher the level, the more important are the exports as a destination for the domestic production of the crop.

We defined four categories based on the Lafay Index interpretations to divide the PGRFA under analysis: undetermined, low, medium, and high. The category undetermined means unavailable information in the database, represented by a singleton function. The data had a range of values between − 0.07 and 5.28. Then, we used trapezoid membership functions for the low (Lafay < 1), medium (Lafay index = 1), and high (Lafay index > 1) categories, for the PGRFA analyzed (see Table 1 for details about the function parameters).

#### Municipality coverage

The municipality coverage (%) is the percentage of Colombian municipalities that cultivate a specific species. This coverage describes both the concentration and adaptability of each species at a regional level. Thus, values closest to 100% represent widely cultivated crops that are the main base on the farmers’ incomes. We calculated it as $CM_i=(M_i/TM) \times 100,$ where $CM_i$ is the municipal coverage for the species i (as a percentage). $M_i$ is the number of municipalities where the species i is cultivated. Moreover, *TM* is the total number of Colombian municipalities. Using this equation, we determined the classes to separate the PGRFA under analysis thus: undetermined (singleton membership function), and narrow coverage, medium coverage, and large coverage, with a trapezoid membership function (see Table 1 for details about the function parameters).

#### Yield

The yield (t ha^−1^) represents agriculture productivity per area measured as $Ri=Pri/Ai.$ Where *Ri* is the yield of the species i (t ha^−1^), *Pri* is the average annual production in 10 years (2007–2017) of the species i (t), and *Ai* is the harvested mean area for the species i in 10 years (2007–2017) in ha in Colombia.

We labeled this data set in four categories: Undetermined, low, medium, and high. The Undetermined category indicated the absence of data in the Agronet database (MADR 2017). For the other three categories (i.e., low, medium, and high), we grouped the species in crop yield according to the FAO categories of food (FAO 2020) (Table 1).

We found considerable differences in scales for yield across all the nine FAO groups. Therefore, we had to normalize all the data before applying fuzzy logic. We used *k*-means (*k* = 3) for the three categories high, medium, and low from the package CKmeans.1d.dp (Wang and Song 2011) to obtain the centroids separately within each of the nine FAO groups. The membership function was a singleton for the undetermined category and a trapezoid for the other three categories (i.e., low yield, medium yield, and high yield) (see Table 1 for details about the function parameters).

#### Economic benefits fuzzy rules

The economic benefits index integrates four variables: the Lafay index, yield (t ha^−1^), income (USD ha^−1^), and municipality coverage (%). Each of these four variables had four options for each label and a unique value assigned through their membership function in order to classify PGRFA under analysis (Table 1). Therefore, we defined rules for the 256 different combinations (four variables with four options each) to describe each PGRFA’ economic benefits within this study.

We calculated a category of economic benefits as output for the 256 rules by using the equation $ID=\sum _{z=1}^{Y}(S_z w_z )\times 100$; Y = 4. The *ID* is the decision index, and $S_z$ represents the score for each variable (i.e., 0 for not information, 1 for low, 2 for medium, and 3 for high). Moreover, the $w_z$ is the weight of each variable, which is 0.25 where $\sum w_z =1$. Under this equation, we used a trapezoid membership function to represent four different classes. If $ID < 0.24,$ the output class was "no decision". If $0.25< ID <0.45$ the output class was low economic benefits. If $0.46< ID <0.64$ the output class was medium economic benefits. Finally, if $0.65<ID <1$, the output class was of high economic benefits. These three categories also represent the three priority levels for the PGRFA analyzed for this pillar (Table 1).

### Food security importance

The second Sustainable Development Goal (SDG) challenges the world to achieve food security and improve nutrition by 2030 (UNDP 2021). Consequently, we adopted a multidimensional approach to evaluate four variables for food security for each PGRFA in this study. They are affordability based on nutrients, nutritional contribution, being part of the government priority list, and the traditional consumption list by regions. There may be dependencies between variables within the food security importance, as multiple factors may influence each variable. We used the information of each variable separately to organize each PGRFA into categories within the BGVCOL and NCB groups. However, we only ranked the PGRFA after integrating the four variables into a food security importance index based on fuzzy logic.

#### Nutritional contribution

Colombia has made significant economic and social progress in recent decades. Although its current classification is an upper-middle-income country, there are still considerable challenges in achieving a convergence toward higher living standards. In this sense, micronutrient deficiencies continue to prevail among children under five years of age and contribute to the deterioration of child development and the increase in the national disease burden (Pinzón-Rondón et al. 2019). We evaluated the 275 PGRFA based on their contribution to the daily nutrient intake requirements to guarantee three micronutrients’ (calcium, iron and zinc) dietary sufficiency in the country’s child population.

We developed two equations based on the nutritional composition of each species (ICBF 2018), the daily dietary target of three deficient micronutrients in the Colombian population (i.e., Calcium, Iron, Zinc) de Bienestar Familiar (ICBF) (2015), and the consumption of each species per day de Bienestar Familiar (ICBF) (2015). First, we calculated the average daily contribution $AD_{ij}$ of the species *i* based on its micronutrients *j*. We estimated $AD_{ij}=(CD_i \times M_j)/100$ where $CD_i$ represented the dairy consumption of the species i in grams (g/day) and $M_j$ was the average composition of the micronutrient *j* in 100 gs of edible food (mg of *j* /100 g of *i*). Then, we estimated the contribution of species *i* to the daily requirement of micronutrient *j* in percentage (%) thus: $C_{ij}=AD_{ij}/MN_j \times 100$. In the equation $C_{ij}$ was the contribution of species *i* to the daily micronutrient *j* requirement (in percentage). The $AD_{ij}$ was the average nutritional contribution of micronutrient *j* during a day (mg /day), and the $MN_j$ was the micronutrient target *j* in a day (mg day^−1^).

We defined a fuzzy system for the global contribution, where inputs are an average daily contribution by each micronutrient (i.e., Calcium, Iron, Zinc, and energy contribution). We settled on four categories: undetermined (absence of information), low, medium, and high average daily contribution of each micronutrient. The undetermined type for all micronutrients had a singleton membership function, while the other three had trapezoid membership functions. Then, we normalized the available data for each micronutrient in values from 1 to 100. Finally, we defined membership function parameters based on normalized data quartile distribution (Table 1).

Same as the fuzzy rules defined for Economic benefits, the output category of nutritional contribution was the following. If $ID<0.24$, then the species has not enough information to decide its nutritional contribution. If $0.25< ID <0.45$ the species had low nutritional contribution. If $0.46< ID <0.64$ the species had medium nutritional contribution. Finally, if $0.65< ID <1$, the species had a high nutritional contribution (Table 1).

#### Affordability based on its nutrients

We defined the affordability of the 345 PGRFA in monetary terms to fill micronutrient deficiency with local resources. We calculated the nutrient-price ratio as the consumer price in USD for every 100 gs of an edible portion in four variables: units of Calcium (mg), units of Iron (mg), units of Zinc (mg), and units of energy (kcal). Then, we estimated the species’ competitiveness from the nutritional and market standpoint using these values.

We defined a fuzzy system to obtain affordability by species; in this case, inputs are the average price per micronutrient (i.e., energy contribution, Calcium, Iron, and Zinc). Available data for each micronutrient were separately normalized to the range from 1 to 100, and then, based on normalized data quartile distribution, we defined membership function parameters. We categorized the data as undetermined (information absence), low, medium, and high. The unknown category had a singleton membership function, whereas we used trapezoid membership functions for the other three ranking types (Table 1).

Finally, we calculated a category of nutritional contribution by combining the four nutrients’ affordability. We used the same approach to define the fuzzy rules in the Economic benefits with the same ID thresholds to estimate the output class for the combination of variables. Thus, the output classes had four target categories: undetermined, low, medium, and high. The species for the increased nutrient affordability, and the higher percentage of the nutritional target of each micronutrient, correspond to the species with higher priority (Table 1).

#### Government priority list

In 2013, the Colombian Government defined a list of plant genetic resources to improve their stable consumption in the Colombian population’s diet and guarantee policies to improve their production and supply (Plan Nacional de Seguridad Alimentaria y Nutricional (PNSAN) 2012) (Fig. S1). We use this information to construct a singleton membership function with two labels, “included” and “not included” (Table 1).

#### Traditional consumption by regions

This variable linked the agricultural and food traditions that reflect Colombia’s multicultural, multi-ethnic, and biodiverse nature across 11 geographic regions (MinCultura 2012) (Fig. S2). We obtained a numerical variable based on this information that shows the number of regions where each species is essential for the food tradition. We assigned a label with three categories based on the number of areas where each species is listed. Narrow use (i.e., Between one and three regions), medium use (i.e., Between four and six areas), and ample use (i.e., between seven and 11 regions).

The membership function was a singleton for the undetermined category and corresponded to missing data. In addition, the membership function for the other three categories (i.e., narrow, medium, and large use) was a trapezoid. Moreover, we applied range normalization over available data from one to eight into one to 100 (Table 1).

#### Food security fuzzy rules

We constructed the food security indicator for each plant species by integrating four variables. First, the government priority list ($A_p$) with two labels: included or not included (Plan Nacional de Seguridad Alimentaria y Nutricional (PNSAN) 2012). Second, traditional consumption ($T_c$) contains narrow, medium, and extensive use (MinCultura 2012). Third, the nutritional contribution ($C_N$) with four labels: low, medium, high, and undetermined (ICBF 2018). Finally, the affordability of the nutrients ($P_N$) with four tags: undetermined, low, medium, and high. Thus, the number of combinations by each variable and label was $A_p (2) \times T_c (3) \times C_N (4) \times P_N (4)=96$.

Then, we calculated an index value using the equation $ID=\sum _{z=1}^Y(S_z w_z) \times 100$; Y=4 (same as the Economic benefits fuzzy rules). The *ID* is the decision index. The $S_z$ represents the score of each variable, and it has different values depending on the labels. For the labels not included narrow use and undetermined then, $S_z=0.$ For the labels medium use and low, then $S_z=1.$ Moreover, for the labels included, extensive use and high, then $S_z=3.$ Finally, the $w_z$ is the weight of each variable, which is 0.25, from 0 to 1 where $\sum w_z=1$. Under this equation, if $ID < 0.24$, the species had no information to decide its food security importance. If $0.25< ID <0.44$ the species had low food security importance. If $0.45< ID <0.64$ the species had medium food security importance. If $0.65< ID <1$, the species had high food security importance (Table 1).

### Data imputation and levels of uncertainty for each PGRFA analyzed

Many of the PGRFA conserved at the BGVCOL had limited data about economic benefits (62%) and food security importance (73%). A reliable solution for the absence of information was to impute the values exclusively for the BGVCOL group. Within the BGVCOL group, we had available information to fill the missing data from either the closest representative crop or the closest species from the same genus (i.e., imputation from the gene pool - GP) or the same food category defined by FAO (i.e., The provisional crop group - PCG). In contrast, this was not the case for the NCB group because they represented unique crops in most cases. Therefore, searching for potential GP or PCG implied selecting species outside the 70 PGRFA from the Government databases. Thus, in the absence of data for PGRFA within the NCB group, we directly classified them as undetermined.

We applied this imputation strategy within the BGVCOL group to three pillars: geographic origin, economic benefits, and food security importance. We omitted the vulnerability pillar because these variables are unique for each species. Therefore, the levels of vulnerability between crops and wild relatives could be opposite, even if they are in the same genus or the same FAO food category.

The procedure followed this sequence. If there was missing information for the PGRFA within the BGVCOL group, we searched a species within the GP group as the first option. If there were no available species within the GP group, we imputed the data using the PCG group. For this case, we chose a species that is the worst-case scenario in terms of data to avoid bias due to the imputation (Table 2). Finally, if there were no GP nor PCG groups to impute, the PGRFA was undetermined. In parallel to the imputation process, we also assigned a tag of uncertainty. If the information exists for the PGRFA or was undetermined, the given tag was "reliable". In contrast, there were two possible tags when the information was missing, and we imputed the data. The label was "GP uncertainty" if we imputed from the gene pool (GP) or "PCG uncertainty" if we imputed with the provisional crop group defined by FAO (Table 1, Fig. 2B).

**Table 2:** The list of crop groups and the number of species without information imputed using one species within the National Plant Germplasm Bank (i.e., BGVCOL group) for the economic and food security pillars

Crop group	Number of species without information	Imputation process	
		Selected species	Group that represents the chosen species
Pepper	7	*Capsicum annuum*	Gene Pool
Annona	5	*Annona muricata*	Gene pool
Cotton	3	*Gossypium hirsutum*	Gene Pool
Cocoa	3	*Theobroma cacao*	Gene Pool
Citrus	19	*Citrus paradisi*	Provisional crop group
Cucurbit	6	*Cucurbita moschata*	Gene Pool
Bean	3	*Phaseolus vulgaris*	Gene Pool
Lulo	14	*Solanum quitoense*	Gene Pool
Blackberry	5	*Rubus urticifolius*	Provisional crop group
Musaceae	9	*Musa Acuminata*	Provisional crop group
Yam	5	*Dioscorea esculenta*	Gene Pool
Potato	24	*Solanum tuberosum*	Gene Pool
Passiflora	23	*Passiflora maliformis*	Provisional crop group
Tree tomato	9	*Solanum betaceum*	Gene Pool
Tomato	3	*Solanum lycopersicum*	Gene Pool
Golden berry	12	*Physalis peruviana*	Gene Pool
nuts	13	*Hymenaea courbaril*	Provisional crop group

There were two possible options to impute. The first option was the gene pool, used when there is a species in the study list representing the closest representative crop or the closest species from the same genus. In the absence of the closest species o genus, the option was the provisional crop group. They are the species that share the same food category defined by FAO. For the species not currently conserved in the BGVCOL (i.e., NCB group), we did not impute any species

### Fuzzy logic rules used for each group of information

Once we calculated the variables of each one of the four pillars and the level of uncertainty for each one, we defined the rules for the final ranking of interest for the 345 PGRFA list (i.e., 275 conserved in the BGVCOL and 70 from the NCB group). We used a Gaussian membership function for the output classes and used a centroid to map from the membership function into a value that is the BGVCOL interest.

If 4/4 or 3/4 of the pillars had a "high" label, the PGRFA interest was ranked as high priority. If the geographic origin had a medium or "high" tag, and 2/3 of the other three pillars had tags higher or equal than the category medium, we ranked the PGRFA as medium priority. Otherwise, we ranked the PGRFA as low priority (Table 1).

## Results

**Fig. 3:**
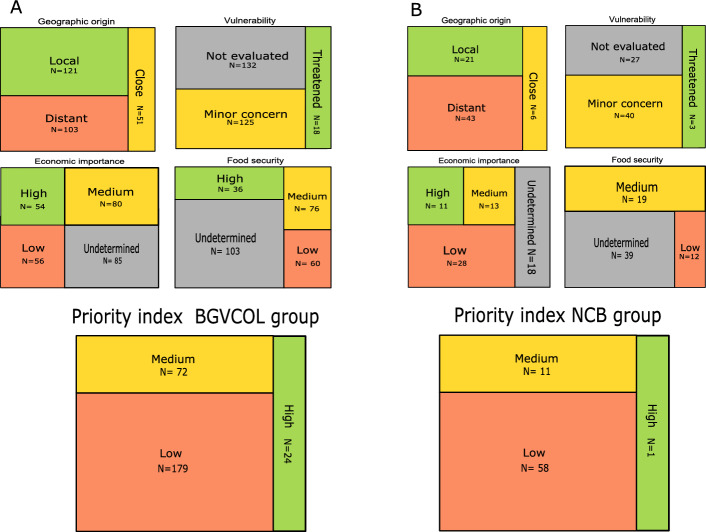
The final classification of 345 plant genetic resources for food and agriculture (PGRFA) in different categories within the four pillars shows the number and the percentage. **A** the 255 PGRFA conserved in the National Plant Germplasm Bank after imputation (i.e., BGVCOL group) and **B** the 70 PGRFA not currently conserved in the BGVCOL (i.e., NCB group). The geographic origin pillar had three categories: Local (green), Close (yellow), and Distant (red). The Vulnerability pillar had three categories: Threatened (green), minor concern (yellow), and not evaluated (grey). The Economic importance pillar had four categories: High (green), medium (yellow), Low (red), and undetermined (grey). Food security importance had four categories: High (green), medium (yellow), Low (red), and undetermined (grey). Finally, the priority index that combined the four pillars had three categories: High (green), medium (yellow), and low (red)

**Fig. 4:**
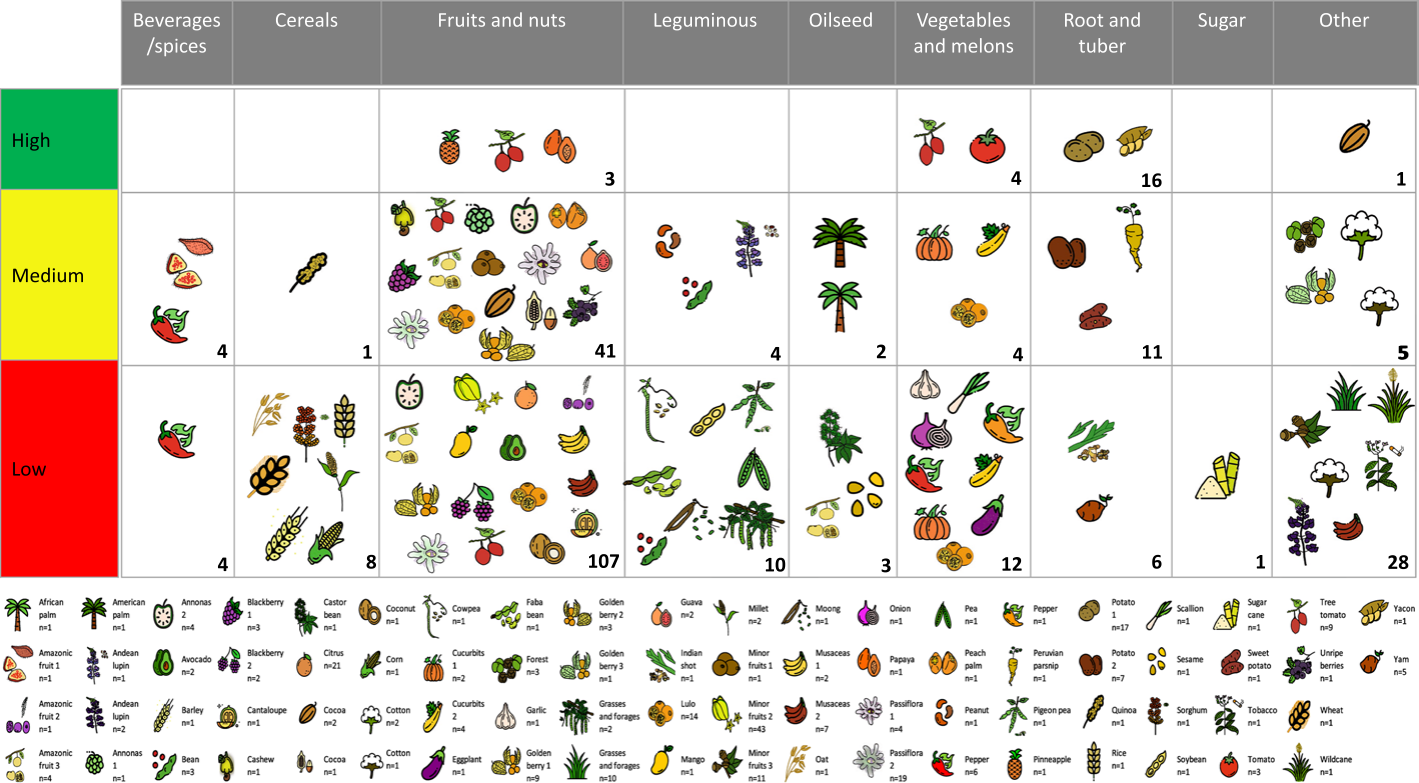
The priority index result for the 275 plant genetic resources for food and agriculture (PGRFA) conserved in the National plant Germplasm Bank (i.e., BGVCOL group). The columns represent the FAO categories for food species. The rows represent the three levels of priority for research investment; thus: 24 in high priority (green), 72 in middle priority (yellow), and 179 in low priority (red). The icons represent each taxa group, and the number below the icon represents the number of species for each taxon (The icons references are in Table S2)

The analysis of all pillars of information generated an index that ranked the 345 PGRFA with values from 0 to 100. Of the 275 PGRFA from the BGVCOL group, 24 are high priority (8.72%), 72 are medium (26.18%), and 179 are low priority (65.09%) (Fig. 3). The 24 PGRFA in the high priority class represents seven taxa: potato (15 species), tomato (3 species), tree tomato (2 species), pineapple, cocoa, papaya, and yacon (Fig. 4). Of the 24 PGRFA, 20 are from the *Solanum* genus, also the most represented genus at the BGVCOL (Fig. 1). Moreover, these crops belong to four FAO food categories: root and tubers, fruits and nuts, vegetables and melons, and other crops. The final ranking for the 70 NCB not conserved in the BGV leads to one species (1.42%), coffee (i.e., *Coffea arabica*, from the FAO’s group beverage and spice crops), in the high priority class. Furthermore, 11 (15.72%) resulted in the medium interest and 58 (82.86%) in the lowest level. Below, we present the results obtained by each pillar after applying the fuzzy logic, organizing the tendencies of the FAO food groups (i.e., median, mean, and standard error), and emphasizing the PGRFA we identified as a high priority for research and conservation investment.

### Geographic origin

This pillar had the smallest undetermined category with only one species without information about their geographic origin (i.e., *Solanum faoensis*). They corresponded to 0.28% of the 345 PGRFA species from the two groups. We imputed it in the category of close origin, considering the broad distribution of the genus *Solanum* in Central and South America. After imputation of this species, none of the PGRFA analyzed was in the undetermined category. Using this information of geographic origin, we found that the BGVCOL conserved PGRFA from 21 regions of the 26 worldwide, whereas the NCB group represented 16 regions (Fig. S3).

The 44% (n = 121) PGRFA conserved in the BGVCOL are local and therefore had high priority because they originated in Tropical South America (n = 88) and the Andes (n = 33), two regions where Colombia is localized. Moreover, 18.55% (n = 51) of PGRFA originated from Central America and Mexico, Caribbean and the three imputed, representing a close origin and middle priority. The other 37.45% (n = 103) originated from distant regions and are of low importance (Fig. 3 and S3A). From the high priority list (n = 121), 70 are fruits and nuts, 20 are roots and tubers, nine are vegetables and melons, five are beverages and spices, four are leguminous, three are oilseeds, one is cereal, and nine correspond to other crops (Fig. S12A).

In the case of the 70 PGRFA from the NCB group, 61% (n = 43) originated from distant regions and therefore had low priority. Moreover, 30% (n = 21) are local (i.e., 17 formed in Tropical South America and four from the Andes) with high priority. Finally, 8.5% (n = 6) had the closest origin (Central America and Mexico) with an intermediate focus (Fig. S3B). The high priority NCB (n = 21) represents seven FAO food categories, thus: four beverage and spice crops, four fruits and nuts, one leguminous, two oilseeds, five roots and tubers, one vegetable and melons, and four classified as other crops.

### Vulnerability status

From the 345 PGRFA of the study, we found that 48% (n = 132) of the BGVCOL group and 38.6% (n = 27) of the NCB group do not have data associated with their vulnerability state across the databases analyzed. The FAO food categories with more absence data about the vulnerability status of the PGRFA analyzed were sugar crops (n = 1), vegetables and melons (n = 20), and tubers and roots (n = 33) (Fig. S4A). In terms of the geographic region of origin, more missing data was in Tropical South America (n = 34), followed by Central America and Mexico (n = 22) and the Andes (n = 25) (Fig. S4B). The missing data corresponded to the undetermined category for this pillar, and we did not apply an imputation strategy. After the fuzzy logic process, we found that 6.5% (n = 18) of PGRFA conserved in the BGVCOL are in the prioritized threatened category. Moreover, 45.4% (n = 125) had a minor concern and low priority (Fig. 3). The threatened BGVCOL represents five FAO food categories: 13 fruits and nuts, one leguminous, one oilseed, one vegetable and melons, and two classified as other crops (Fig. S12B).

In the NCB group, three PGRFA (4.3%) are in the threatened category with high conservation priority, and 40 (57.1%) have a minor concern. The threatened PGRFA represents two FAO food categories: two beverages and spices (i.e., Coffee - *Coffea arabica, cardamom - Elettaria cardamomum*) and one oilseed (i.e., almond - *Prunus dulcis*).

### Economic benefits

The economic benefits pillar integrated four variables: Lafay index, yield, municipality coverage, and income. For this pillar, we applied an imputation process within the BGVCOL group. After this process, 54 (19.6%) of PGRFA had high priority, 80 (29%) with middle focus, 56 (20.3%) with low priority, and 85 (30.9%) undetermined from the 275 analyzed (Fig. 3). The 54 groups of species represented 15 taxonomic groups with high priority thus: golden berry (n = 13), rice (n = 1), pineapple (n = 1), sweet potato (n = 1), sugar cane (n = 1), tomato (n = 3), potato (n = 23), yacon (n = 1), bunching onion (n = 1), papaya (n = 1), palm (n = 1), cotton (n = 3), bean (n = 1), cocoa nuts (n = 2), and citrus (n = 1). From them, sweet potato, tomato, and sugar cane presented the highest income. Cocoa and citrus had the highest Lafay index. Moreover, sugar cane and cocoa had the highest municipality coverage. Finally, the species with the highest yield groups were rice, pineapple, sugar cane, tomato, potato, palm, cotton, and bean (Fig. S14A). In comparison, for the NCB group, we did not apply an imputation methodology, and the 70 PGRFA distributed thus: 11 (15.7%) with high priority, 13 (18.6%) with middle focus, 28 (40%) with low, and 18 (25.7%) undetermined (Fig. 3). The 11 with high priority were: *Coffea arabica* (coffee), *Zingiber officinale* (ginger), *Raphanus sativus* (radish), *Curcuma longa* (Indian saffron), *Spinacia oleracea* (spinach), *Lactuca sativa* (lettuce), *Opuntia ficus-indica* (fig opuntia), *Beta vulgaris* (sugar beet), *Brassica oleracea* (cabbage), *Fragaria vesca* (wild strawberry), and *Manihot esculenta* (cassava). Below, we describe the mean results of each variable, compiling the PGRFA by the FAO food categories to show the main trends.

#### Imputation process within the BGVCOL group

Within the BGVCOL group, 171 (62%) PGRFA have missing information about the four variables considered in the economic benefits pilar. For this group, 86 PGRFA had either a gene pool (GP) or a provisional crop group (PCG) to impute the values (Table 2). Thus, after imputation, 85 (31%) resulted in undetermined, 54 (20%) high, 80 (29%) middle, and 56 (20%) in the low category of the ranking (Table 3).

**Table 3:** The number of PGRFA conserved in the National Plant Germplasm Bank (i.e., BGVCOL group) separating them by the priority level for the economic importance and food security importance pillars before and after the imputation process for the species without information across all the databases consulted (i.e., on the undetermined category)

Pillar	Label/ priority level	Original count (%) of PGRFA for each label	Redistribution of final count (%) on each label after imputed the PGRFA without information (undetermined)
Economic importance	High	15 (5)	54 (20)
	Medium	39 (14)	80 (29)
	Low	50 (18)	56 (20)
	Undetermined	171 (62)	85 (31)
	Total	275 (100)	275 (100)
Food security importance	High	10 (4)	36 (13)
	Medium	38 (14)	76 (28)
	Low	26 (9)	60 (22)
	Undetermined	201 (73)	103 (37)
	Total	275 (100)	275 (100)

The imputation redistributed the final count for each category. Still, 31% and 37% of PGRFA for each pillar did not have a gene pool or a provisional crop group that could be used for imputation (see Table 2)

#### Income

For the BGVCOL group, we found information about this variable for 55 (20%) of the PGRFA. The visualization of the income (thousand USD/ha) for each PGRFA organized by FAO food categories showed that sugar crops had the highest income, followed by vegetables and melons, roots and tubers, oilseed, beverage and spice, fruits and nuts, other crops, cereals, leguminous (Table 4 and Fig. S6A). In the case of the NCB, 38 (54%) had information for this variable. Separating the PGRFA by FAO food categories, vegetables, and melons had the highest income mean, followed by beverages and spices, Fruits and nuts, and leguminous. In contrast, the roots and tubers had a lower income (Table 4, Fig. S7A).

**Table 4:** The mean and standard deviation of each variable (Income, Lafay Index, Municipality coverage and yield) within the Economic Benefits pillar for the nine FAO food categories were calculated for the species conserved at the BGVCOL and NCB

FAO classification	Income (thousand USD/ha)		Lafay Index		Municipality coverage (%)		Yield (t ha^−1^)	
	BGVCOL	NCB	BGVCOL	NCB	BGVCOL	NCB	BGVCOL	NCB
Beverage and spice	7.99	26.74 ± 16.69	1.0 ± 0	1.09 ± 0.20	0.5	3.55 ± 2.78	1.8	7.5 ± 1.5
Cereals	4.80 ± 1.95	NA	0.42 ± 0.15	NA	19 ± 13.7	NA	4.2 ± 0.7	NA
Fruits and nuts	7.17 ± 0.76	22.77 ± 9.46	1.11 ± 0.09	0.82 ± 0.75	6.4 ± 1.3	8.3 ± 4.2	9.7 ± 0.8	12.3 ± 1.3
Leguminous	3.03 ± 1.16	2.87 ± 0.41	0.71 ± 0.15	0.67 ± 0.33	8.5 ± 7.4	0.1	4.3 ± 1.2	3.7 ± 1.2
Oilseed	11.08 ± 1.77	4.09	1.0 ± 0	1.0 ± 0	11.6	1.3 ± 1.19	17.0 ± 7.5	7.5 ± 5.5
Other crops	5.85 ± 3.66	NA	1.20 ± 0.20	1.0 ± 0	13.6 ± 11.6	5.75 ± 5.45	3.6 ± 1.7	1.5 ± 0.5
Roots and tubers	12.74 ± 6.44	3.45	1.0 ± 0	1.0 ± 0	9.6 ± 4.3	20.17 ± 18.08	12.8 ± 2.2	10.8 ± 0.9
Sugar crops	37.73	NA	1.03	1.0	60	0.1	60.1	3.8
Vegetables and melons	16.11 ± 4.00	44.64 ± 22.85	0.91 ± 0.08	0.93 ± 0.77	9.8 ± 4.3	2.0 ± 0.7	21.8 ± 2.6	20.2 ± 3.7

The abbreviation “NA” is used when information is not available

#### Lafay index

For the BGVCOL group, we found only the information to create this index for 88 (32%) species. Therefore, we imputed using other PGRFA within the BGVCOL corresponding to the GP or PCG group (Table 2). After the imputation process, the Lafay index mean by FAO food categories showed that cereals, leguminous and vegetables, and melons have a production not enough for the country requirements (i.e., mean <1). Therefore, they represent the low-priority group because importing them is mandatory. The opposite case is the species in the category of other crops, the fruits and nuts, indicating that Colombia mainly exports them (i.e., mean > 1) and has high priority. Finally, the sugar crops, roots and tubers, oilseed, and beverage and spice showed equal production and internal consumption (i.e., mean = 1) with middle priority (Table 4, Fig. S6B).

In the case of the NCB, 61 (87%) of the PGRFA had information to generate this index. Separating them by FAO food categories, we found that Leguminous, fruits and nuts, and vegetables and melons have mean values < 1, indicating that the country is importing this PGRFA because of the low national production. Therefore, they have a low priority for this index. The other categories, such as sugar crops, roots and tuber, oilseed, and other crops indicating similar production and consumption in the country with middle priority (Table 4, Fig. S7B).

#### Municipality coverage

For the BGVCOL group, we found information about this variable for 85 (31%) of the species. The visualization of the municipality coverage (%) for each PGRFA organized by FAO food categories showed that sugar crops had the highest coverage, followed by cereals. The other categories had a municipality coverage around 10%. They included oilseed, roots and tubers, vegetables and melons, other crops, fruits and nuts, leguminous, and beverage and spice (Table 4, Fig. S6C).

In the case of the NCB, 36 (51%) of the PGRFA had information for this variable. Separating them by FAO food categories, we found that other crops, fruits, nuts, vegetables, and melons have a low coverage (i.e., ~ 10%). Moreover, sugar crops (n = 1) and leguminous (n = 3) did not have any municipality coverage in the country, with a mean of 0.1% for both categories (Table 4, Fig. S7C).

#### Yield

For the BGVCOL group, we found information about this variable for 115 (42%) of the species. The yield (t ha^−1^) mean by FAO food categories showed that sugar crops had the highest yield compared with the other categories. The vegetable and melons, oilseed, and roots and tubers had a yield higher than > 10 t ha^−1^. In contrast, the fruits and nuts, cereals, leguminous, beverage and spice, and other crops had a yield < 10 t ha^−1^ (Table 4 and Fig. S6D).

In the case of the NCB, 66 (94%) of the PGRFA had information for this variable. Separating them by FAO food categories, we found that vegetables and melons, fruits and nuts, roots and tubers had the higher yield mean (i.e., > 10 t ha^−1^. In contrast, oilseed, beverage and spice, sugar crops, leguminous and other crops had the lower mean (i.e., < 10 t ha^−1^) (Table 4 and Fig. S7D).

### Food security importance

The food security pillar was composed of four variables: government priority list, traditional consumption, nutritional contribution, and affordability based on its nutrients. For the 275 PGRFA from the BGVCOL, we obtained after imputation 36 (13%) in high importance, 76 (27.6%) in medium, 60 (21.81%) in low, and 103 (37.45%) undetermined (Fig. 3). The 36 groups of species with high importance included rice, cocoa, cucurbit, mango, pineapple, papaya, sweet potato, bean, guava, and potato. Except for pineapple and cocoa, all the species show lower affordability based on their nutrients (i.e., higher importance). The nutritional contribution is also very high for all 36 taxa, rice and potato with lower values than the others. Moreover, all species are important in the Government prioritizing the list. Finally, rice is essential for traditional consumption, with a median value for pineapple, beans, and potatoes. The other cocoa, cucurbit, mango, papaya, sweet potato, and guava had less importance for this factor (Fig. S13B).

In the case of the 70 PGRFA from the NCB group, no one resulted in the category of high importance; 19 (27.1%) in the medium, 12 (17.1%) in the low, and 39 (55.7%) were undetermined, without any imputation process (Fig. 3). Below, we describe the results of each variable, compiling the PGRFA by the FAO food categories to show the main trends.

#### Imputation process within the BGVCOL group

For the BGVCOL group, 201 (73%) PGRFA did not have any information about the importance of food security importance pilar (i.e., undetermined). 98 PGRFA had either a gene pool or a provisional crop group to impute the values (Table 2). Therefore, 103 (37%) were undetermined. Moreover, after imputation, 36 (13%) were high, 76 (28%) middle, and 60 (22%) in the low ranking of prioritization (Table 3). In the case of the NCB, we did not impute any of the 70 PGRFA.

#### Affordability based on its nutrients

To analyze this variable, we used information about three micronutrients, Calcium (Ca), Iron (Fe), Zinc (Zn), and units of energy. In the BGVCOL group, we found that the information about this variable was unbalanced for each nutrient considered. For Calcium, we found available data in 64 (23%), Iron 64 (23%), Zinc 29 (11%), and units of energy 67 (24%) from the 275 PGRFA analyzed in this group (Fig. S8).

The visualization of the affordability of nutrients organized by FAO food categories showed that the cheapest USD per 100 g of edible portion for Ca were sugar crops, leguminous crops, vegetables, and melons, followed by cereals, fruit, and nuts. The most expensive Ca by 100 g of the edible portion was for beverages, spices, other crops, roots, and tubers (Table 5 and Fig. S8A). In the case of Fe, the cheapest category was for sugar crops, followed by leguminous crops, fruits and nuts, cereals, vegetables and melons, and other crops. The most expensive categories were beverages, spices, roots, and tubers (Fig. S8B). Moreover, the cheapest USD for 100 g of edible portion with Zn was sugar crops, followed by cereals, vegetables and melons, fruits and nuts, and leguminous. The most expensive categories (i.e., > 0.4 USD) were roots and tubers and other crops (Table 5, Fig. S8C). Finally, regarding energy, the cheapest category was sugar crops, followed by cereals, fruits and nuts, roots and tubers, and vegetables and melons. The most expensive categories (i.e., > 3.6 × 10^−3^ USD) were other crops and beverages, and spices (Table 5, Fig. S8D). The affordability of nutrients indicated that 36 (13.1%) are highly prioritized with lower expenses, 6 (2.2%) are middle, 20 (7.6%) in the lower priority because of their higher cost, and 212 were undetermined for this variable.

In comparison, in the case NCB group, we found that the information about this variable was unbalanced for each nutrient considered in the study. For Calcium, we found available data in 23 (33%), Iron 21 (30%), Zinc 8 (11%), and units of energy 19 (27%) from the 70 PGRFA analyzed (Fig. S9). Visualizing the micronutrients that composed this variable organized by FAO food categories, we found that the cheaper USD per 100 edible portions for Ca was roots and tubers and leguminous, followed by vegetables and melons, and fruits and nuts. The most expensive category was beverage and spice (Table 5, Fig. S9A). In the case of Fe, the cheaper category was leguminous, followed by roots and tubers, vegetables, and melons. The highest cost was for fruits, nuts, beverages, and spices (Table 5, Fig. S9B). Furthermore, the cheaper category for Zn was roots and tubers, followed by vegetables and melons. The highest cost was for fruits and nuts (Table 5, Fig. S9C). Finally, in the case of energy, the cheaper value was for roots and tubers, followed by fruits and nuts, beverages and spices, and leguminous. The most expensive category was vegetables and melons (Table 5, Fig. S9D). The analysis of the affordability of nutrients by categories indicated that 12 (17.4%) resulted in the higher priority with low cost, 5 (7.1%) in the middle priority, and 4 (5.7%) in the lower priority because of their higher price, and 49 (70%) were undetermined for this variable.

**Table 5:** For the Food Security pillar, the average and standard deviation of the affordability variable were computed for each of the nine FAO food categories for species conserved at BGVCOL and NCB

FAO food categories	Zn (USD/100 g edible portion)		Ca (USD/100 g edible portion)	
	BGVCOL	NCB	BGVCOL	NCB
Beverage and spice	NA	NA	7.3 × 10^−3^	4.5 × 10^−3^ ± 4.0 × 10^−3^
Cereals	6.6 × 10^−2^ ± 2.5 × 10^−2^	NA	4.7 × 10^−3^ ± 1.4 × 10^−3^	NA
Fruits and nuts	1.6 × 10^−1^ ± 4.7 × 10^−2^	6.1 × 10^−1^ ± 4.2 × 10^−2^	5.5 × 10^−3^ ± 2.0 × 10^−3^	9.2 × 10^−3^ ± 3.0 × 10^−3^
Leguminous	2.3 × 10^−1^ ± 5	NA	1.5 × 10^−3^ ± 6.4 × 10^−4^	1.0 × 10^−3^
Oilseed	NA	NA	NA	NA
Other crops	6.0 × 10^−1^ ± 0.49	NA	8.1 ± 1.7 × 10^−3^	NA
Roots and tubers	3.7 × 10^−1^ ± 1.1 × 10^−1^	5.4 × 10^−2^	1.1 × 10^−2^ ± 3.6 × 10^−3^	1.0 × 10^−3^ ± 1.0 × 10^−3^
Sugar crops	4.5 × 10^−2^	NA	3.3 × 10^−4^	NA
Vegetables and melons	1.2 × 10^−1^	1.6 × 10^−1^ ± 2.2 × 10^−2^	1.9 × 10^−3^ ± 7.1 × 10^−4^	6.5 × 10^−3^ ± 3.9 × 10^−3^

FAO food categories	Energy (USD/100 g edible portion)		Fe (USD/100 g edible portion)	
	BGVCOL	NCB	BGVCOL	NCB
Beverage and spice	3.9 × 10^−3^	1.9 × 10^−3^ ± 9.4 × 10^4^	3.1 × 10^−1^	1.8 ± 1.6
Cereals	4.9 × 10^−4^ ± 2.3 × 10^−3^	NA	6.7 × 10^−2^ ± 2.8 × 10^−2^	NA
Fruits and nuts	8.3 × 10^−4^ ± 2.3 × 10^−4^	1.9 × 10^−3^ ± 3.8 × 10^−4^	6.2 × 10^−2^ ± 1.5 × 10^−2^	1.4 × 10^−1^ ± 3.2 × 10^−2^
Leguminous	1.27 × 10^−3^ ± 9.0 × 10^4^	2.2 × 10^−3^	4.6 × 10^−2^ ± 2.1 × 10^−2^	2.4 × 10^−2^
Oilseed	NA	NA	NA	NA
Other crops	3.2 × 10^−3^ ± 1.5 × 10^−3^	NA	1.1 × 10^−1^ ± 8.3 × 10^−3^	NA
Roots and tubers	1.7 × 10^−3^ ± 1.1 × 10^−3^	4.6 × 10^5^ ± 4.6 × 10^5^	3.2 × 10^−1^ ± 2.5 × 10^−1^	3.2 × 10^−2^ ± 3.0 × 10^−2^
Sugar crops	1.7 × 10^−4^	NA	1.3 × 10^−2^	NA
Vegetables and melons	2.1 × 10^−3^ ± 5.3 × 10^−4^	3.7 × 10^−3^ ± 1.4 × 10^−3^	1.1 × 10^−1^ ± 2.6 × 10^−2^	4.9 × 10^−2^ ± 1.9 × 10^−2^

If information was not available, “NA” was used as an abbreviation

#### Nutritional contribution

We used information about three macronutrients, Calcium (Ca), Iron (Fe), Zinc (Zn), and units of energy. The availability of this information was unbalanced for each nutrient considered. In the BGVCOL group, the PGRFA with available data was for Calcium in 200 (73%), Iron 184 (67%), Zinc 102 (37%), and units of energy 198 (72%) from the 275 PGRFA analyzed in this group (Table 6, Fig. S10). The visualization of this variable organized by FAO food categories separating each micronutrient showed that the higher percentage of the daily nutritional target for Ca was beverage and spice, sugar crops, and vegetables and melons. The other categories had a percentage of Ca <10%. They are in descendant order leguminous, cereals, fruits and nuts, other crops, roots and tubers, and oilseed (Table 6, Fig. S10A). In the case of Fe, the categories with a higher percentage of the daily nutritional target (i.e., >10%) were beverage and spice, sugar crop, cereals, and leguminous. The other categories with <10% included fruits and nuts, other crops, vegetables and melons, oilseed, and roots and tubers (Table 6, Fig. S10B). Moreover, for Zn, the highest percentage of the daily nutritional target was cereals, fruits, and nuts. The other categories with <10% included oilseed, roots and tubers, leguminous, other crops, and vegetables and melons (Table 6, Fig. S10C). Finally, for energy, the highest percentage of the daily nutritional target (i.e., >10%) were cereals, sugar crops, other crops, and leguminous. The other categories with <10% included roots and tubers, fruits and nuts, beverages and spice, oilseed, and vegetables and melons (Table 6, and Fig. S10D). The analysis of the nutritional contribution by priority categories indicated that 25 (9.1%) resulted in the higher priority with higher contribution, 17 (6.2%) in the middle priority with middle contribution, and 18 (6.5%) in the lower priority because of their lower contribution, and 214 were undetermined for this variable.

**Table 6:** The nutritional contribution variable was analyzed for each of the nine FAO food categories for species conserved at BGVCOL and NCB under the Food Security pillar, with the average and standard deviation being calculated

FAO food categories	Zn (% of the daily nutritional target)		Ca (% of the daily nutritional target)		Energy (% of the daily nutritional target)		Fe (% of the daily nutritional target)	
	BGVCOL	NCB	BGVCOL	NCB	BGVCOL	NCB	BGVCOL	NCB
Beverage and spice	NA	60.0	34.6 ± 4.9	16.0 ± 9.2	3.8 ± 1.1	36.3 ± 32.3	26.8 ± 3.2	28.7 ± 25.9
Cereals	40.1 ± 17.1	NA	4.5 ± 1.1	NA	24.5 ± 3.4	NA	19.6 ± 9.1	NA
Fruits and nuts	23.6 ± 6.8	5.7 ± 2.4	3.2 ± 0.4	5.2 ± 2.9	5.7 ± 0.6	4.5 ± 0.6	8.1 ± 0.9	5.9 ± 1.7
Leguminous	6.0 ± 2.2	NA	9.7 ± 3.7	12.5	15.8 ± 6.8	3.0	17.1 ± 7.8	24.6
Oilseed	10.0	NA	0.7	NA	3.0	NA	6.2	NA
Other crops	6.86 ± 0.37	NA	1.0 ± 0.3	NA	4.9 ± 0.7	NA	1.04 ± 2.8	NA
Roots and tubers	7.4 ± 0.5	12.5	1.0 ± 0.2	3.4 ± 1.2	7.1 ± 0.4	16.7 ± 7.7	4.1 ± 0.3	11.6 ± 8.4
Sugar crops	19.4	NA	17.6	NA	17.1	NA	20.9	NA
Vegetables and melons	3.8 ± 0.6	13.7 ± 5.5	14.4 ± 5.2	9.1 ± 5.4	2.7 ± 0.5	2.5 ± 0.6	6.6 ± 1.7	25.9 ± 12.3

In cases where information was unavailable, “NA” was used as an abbreviation

In comparison, in the NCB group, the available data for Calcium was 26 (37%), Iron 23 (33%), Zinc 8 (11%), and units of energy 21 (30%) from the 70 PGRFA analyzed in this group (Fig. S11). The visualization of this variable organized by FAO food categories separating each micronutrient showed that the higher percentage of the daily nutritional target for Ca was beverage and spice, and leguminous. The other categories with <10% included vegetables and melons, fruits and nuts, and roots and tubers (Table 6, Fig. S11A). In the case of Fe, the higher percentage of the daily nutritional target was beverage and spice, vegetable and melons, leguminous, and roots and tubers. The category with <10% was fruits and nuts (Table 6, Fig. S11B). Moreover, the higher percentages of the daily nutritional target for Zn were beverages and spices, vegetables and melons, and roots and tubers. The category with 10% was fruits and nuts (Table 6, Fig. S11C). Finally, in the case of energy, the categories with a higher percentage of the daily nutritional target were beverages and spices and roots and tubers. The categories with <10% were fruits, nuts, leguminous, vegetables, and melons (Table 6, Fig. S11D). The analysis of the nutritional contribution by priority categories indicated that 10 (14.3%) resulted in the higher priority with higher contribution, 10 (14.3%) in the middle priority with middle contribution, and 7 (10%) in the lower priority because of their lower nutritional contribution, and 43 (61.4%) were undetermined for this variable.

#### Government priority list

From the 345 PGRFA of the study, we found that 8.7% (n = 24) of the BGVCOL group and 10% (n = 7) of the NCB group are on the Colombian government’s priority list must improve their productivity and supply. They represent the label "included" (i.e., *a* = 60) with high priority. The PGRFA absent from this list had the "not included" label (i.e., *a* = 20) and represented a low priority for this variable.

#### Traditional consumption

From the 345 PGRFA of the study, we found that 36.7% (n = 101) of the BGVCOL group and 38.6% (n = 27) of the NCB group are present in at least one of the 11 geographic regions of Colombia. The missing data, 63.3% (n = 174) in the BGVCOL and 61.4% (n = 43) in the NCB group represent the undetermined category.

Of the 101 conserved in the BGVCOL, 21 are in only one region across the country, 22 in two, and nine in three areas. Thus, these 52 PGRFA represent limited use and have low priority. Moreover, 16 are in four regions and 10 in five regions. These 26 represent the middle priority. Finally, seven in seven regions and 16 in eight regions. These 23 represent ample use with high priority. In comparison, of the 27 with information from the NCB group, 10 are in only one region, four are in two regions, and six are in three regions, these with low focus, and seven are in seven regions with high use and priority.

### Limitations: Gaps of information for native crops and wild relatives from the bank

Even with the imputation strategy for the BGVCOL group, 132 PGRFA resulted in the undetermined category because of the absence of information about at least one pillar affecting mainly Fruits and nuts (52%, n = 68) and the local region (43%, n = 23 from the Andes and n = 34 for Tropical South America) (Table S1). The lack of information by pillars was: 48% (n = 132) for the vulnerability status, 37.5% (n = 103) for food security, 30.9% (n = 85) for economic benefits, and zero for geographic origin. In the case of the NCB group, we did not adopt an imputation strategy for the 70 PGRFA. However, we found also lack of information for each pilar thus: 55.7% (n = 39) for food security importance, 38.6% (n = 27) for vulnerability status, 25.7% (n = 18) for economic benefits, and zero for geographic origin.

## Discussion

Colombia is a megadiverse country that hosts 10% of the life diversity on Earth, including plant genetic resources for food and agriculture (PGRFA) (Clerici et al. 2019). Although there have been worldwide efforts to conserve PGRFA, the study of agrobiodiversity for most species remains at an early step worldwide. Germplasm banks preserve thousands of accessions of several species. However, broad unknown gaps are frequent, even for economically valuable crops. Ranking lists using several criteria help highlight what should be taken exceptional care of and considered for studies in conservation and sustainable use of PGRFA even with a limited budget.

We proposed prioritizing the PGRFA and applying a fuzzy logic methodology to the BGVCOL group (i.e., 275 conserved in the national germplasm bank—BGVCOL) and NCB group (i.e., 70 external species considered by the government as essential but never conserved in the BGVCOL). We used a data-driven method to build an index based on four pillars: geographic origin, vulnerability status, economic benefits, and food security importance, dividing BGVCOL and NCB groups of data into three classes: high, middle, and low priority. Except for geographic origin, those pillars must be continually updated as trends evolve and more comprehensive data becomes available for additional species.

Although this methodology is specific to Colombia, other national genebanks can apply this methodology to prioritize species and orientate activities. Below, we explain the main results we obtained and the rationale behind each pillar as a framework to construct research and conservative efforts for the high-priority class. Moreover, we highlight the limitations and future studies to strengthen this methodological approach in the future.

### The geographical origin and vulnerability status

There have been several criteria to sort and prioritize species for collection schemes and long-term ex situ conservation worldwide within germplasm banks or genebanks (Farnsworth et al. 2006; Barazani et al. 2008; Jiménez-Alfaro et al. 2010). The geographic origin (or endemism) and the vulnerability status are standard variables in prioritizing crop species for conservation (Barazani et al. 2008; Farnsworth et al. 2006). The primary rationale for choosing these two criteria is Vavilov’s concept of centers of crop diversity to explain the patterns of evolution and diversification of food crops (Harris 1990; Cohen and Loskutov 2016). Based on this concept, genebanks should be located just in the regions where major PGRFA evolved. They have a high degree of unique genetic variation not found in other territories (Hummer and Hancock 2015). Accordingly, the main goal of germplasm banks is to rescue and long-term preserve native endangered PGRFA from extinction because of habitat loss, genetic erosion, or vulnerability to abiotic or biotic stresses such as global warming, plagues, and diseases (Priyanka et al. 2021).

Colombia is part of the center of diversity for several Andean and north of South American PGRFA. Therefore, it should invest in the research and long-term conservation of native and threatened PGRFA as an opportunity to understand how this genetic variation evolved, how to use it for bioeconomy to improve traits of interest to reduce poverty and hunger, and how to increase their resilience in the face of global warming (Rao and Hodgkin 2002). Indeed, our data showed that the BGVCOL is already conserving local PGRFA, and they represent almost half of the list. This pattern suggests that BGVCOL, since it started, has been conserving unique PGRFA that probably are absent abroad germplasm banks, making this Colombian germplasm bank vital for the agrobiodiversity ex situ conservation of the world. However, we detected significant information gaps about vulnerability status across both data sets, BGVCOL and NCB, especially for local PGRFA. Therefore, we may be missing critical information in our index. The most affected FAO food groups were cereals, fruits and nuts, and beverages and spices.

The absence of basic information about biodiversity is common in tropical regions such as Colombia and occurs across different taxa (21; Collen et al. 2008; Amano and Sutherland 2013; Meyer et al. 2016; Tessarolo et al. 2017; Jara et al. 2018). For example, for small-scale marine fisheries, significant gaps exist in basic biological, and ecological information (Jara et al. 2018). Moreover, in mammals, a conspicuous group in the Caribbean of Colombia showed that 50% of the analyzed regions do not have registers since 1950 (21). Furthermore, the most updated study about edible plants in Colombia found that 17.6% (n = 673) do not have any records. Also, spatial analysis suggests insufficient on-site research that generates an unequal distribution of records across departments and bioregions (Gori et al. 2022). Global tendencies showed that rich countries (i.e., with high per capita gross domestic product—GDP) have more biological records per square kilometer than countries with low GPD, even if these rich countries are not the most biodiverse (Amano and Sutherland 2013). There are several reasons to explain this disparity. For example, the lack of funds, not adequate infrastructure, lack of expertise for data collection, difficulties in accessing places for political reasons or personal security, difficulties to get publishing or giving access to data, and a low proportion of English speakers (Collen et al. 2008; Amano and Sutherland 2013). In Colombia specifically, the security problems and lack of funds are transversal to the 60 years of internal conflict and the instability of government agencies in charge of records (Identifying biodiversity data-gap hotspots within biodiversity rich but data-poor countries (Asociación Colombiana de Zoología 2019; Jara et al. 2018; Gori et al. 2022). Fortunately, within Colombia, the peace agreement with FARC signed in 2016 has opened for national scientists new opportunities to visit those forbidden territories through field expeditions supported by the Ministry of Science, Technology, and innovation in Colombia named “Colombia BIO”. These grants generated basic information across wild species and ecosystems through 20 expeditions between 2016 and 2018 (MINCIENCIAS 2022a). Of them, only one was on PGRFA named "the cacao BIO,” which collected *Theobroma cacao* and wild relatives in regions of probable origin within Colombia (MINCIENCIAS 2022b)

Based on this experience, we encourage research institutions, universities, and organizations with projects focused on quantifying wild biodiversity in several Colombian territories that integrate PGRFA as part of the landscape analysis to find the quality and quantity of data available. Several methodologies in biodiversity research combine coverage and uncertainties in the information to generate indicators for prioritizing significant gaps and actions (Ruete 2015; Meyer et al. 2015, 2016). In Colombia, the methodological approach relating quantity and quality information to generate a research urgency scoring across 271 species of Colombian mammals from the Caribbean region has the potential to be adapted for several PGRFA [21]. Moreover, for the analysis of PGRFA, the ethnobotanical perspective and the participatory sciences are extremely important to find the gaps and priorities of the biodiversity inventory (Gori et al. 2022; Torres-Morales et al. 2021; Meyer et al. 2015; Amano et al. 2016). An important question is what PGRFA is in use and if the rural community or farmer’s markets has noted some changes in the presence/absence of this PGRFA during the last decades. Like many Latin American countries, Colombia has several imbricate problems associated with small farmers’ agriculture and farmer’s markets, besides a long-term internal conflict. They included the economic opening in the 1990 decade and fluctuations in the national and international market (Hylton and Tauss 2016). Therefore, a combined research effort from a multidisciplinary and transdisciplinary perspective that combines local and scientific knowledge could fill missing essential information over time and is also an opportunity to design investment strategies to conserve local biodiversity with a sustainable development perspective (Jara 2018). Indeed, missing information also represents an opportunity to design initiatives where the ex situ BGVCOL effort interacts with communities doing in situ/*on farm* conservation and monitoring the state of PGRFA across time (Rajpurohit and Jhang 2015).

### The economic benefits and food security importance

In contrast with the geographic origin and vulnerability status, as far as we know, the economic benefits and food security importance are not standard variables used to rank PGRFA and make investment decisions about ex situ conservation research. We chose them because they are highly informative nowadays to attend to our particular social-economic circumstances of poverty and hunger in the face of unsustainable agrodiversity. Therefore, both pillars helped us put this problem into the mission of the national germplasm bank.

Colombia reported that in 2021, 39.3% of the national population suffered from monetary poverty, but the average is worse for the rural population with 44.6% (DANE 2021). Moreover, the FAO-WFP report for February to May (FP FAO 2022) alerted that food insecurity in Colombia could deteriorate in the following months by several factors such as political instability, high inflation rates, regional migratory crisis, delays in the implementation of the 2016 peace agreement, and COVID-19 pandemic. Thus, by December 2021, 7.3 million Colombians were in food insecurity or needed food assistance (WFP FAO 2022). These reports reinforce the importance of aligning the mission of BGVCOL to attend to the urgent needs of 22.9% of the rural population (DANE 2022).

Our index found 54 PGRFA from the BGVCOL group from 15 taxonomic groups and 11 from the NCB group highly prioritized for their economic benefits. They have a high yield, income, and export prospect, covering most of the country. Therefore, they have the potential to reduce poverty across rural and agricultural families. In 2020, agriculture contributed 7.6% of GPD (World Bank 2022). Thus, conservation and research investment in these highly prioritized rank PGRFA could increase the income of rural families and provide a direct opportunity for connecting the demobilization of combatants in rural productive projects (World Bank 2018). Moreover, by focusing the investment on the PGRFA’s high highest rank for economic benefits in the areas with the most significant conservation return, it could also facilitate the prevention of forest conversion by agricultural activities that currently are accelerating biodiversity loss within Colombia (Guerrero-Pineda et al. 2022).

Moreover, our index found 36 PGRFA from 10 taxonomic groups conserved in the BGVCOL as highly prioritized for food security, but none within the NCB group resulted in high priority. They represent nutritious and affordable food that is part of our cultural and culinary traditions (Mehta et al. 2010). Most are part of the NUS (Neglected and underutilized species), with very few ethnobotanical studies to promote their use in the Country (Albuquerque et al. 2013; Cámara-Leret et al. 2014). Therefore, they represent an opportunity to be encouraged among researchers, plant breeders, and stakeholders as part of the crops that could help with the SDG goal to reduce poverty and hunger. Unfortunately, rural households within Colombia have progressively replaced this NUS exponentially since the green revolution because of the imposition of a technocratic approach to rural development (Lasso 2021). The consequence has been the disuse of many NUS, including culinary knowledge, the loss of autonomy, and increased poverty for specific communities and territories (Gori et al. 2022). Therefore, studying those identified PGRFA with high potential to solve food security needs to continue strengthening the basic biological studies with ethnographic and sociological perspectives to safeguard this agrobiodiversity legacy (Lasso 2021; Gori et al. 2022).

The economic benefits and food security pillars were included in the index to establish an understanding with decision-makers. However, it is important to note that the BGVCOL conserves a wide range of crops, including profitable crops, wild plants, NUS, and crop wild relatives, which are crucial for ensuring food security. The index integrates four independent pillars, and we proposed an imputation strategy to mitigate the effects of information gaps and avoid bias toward the most profitable crops.

### Present and future actions for PGRFA that resulted in high-prioritized

We applied the index methodology to 345 PGRFA from two sets, the BGVCOL and the NCB. Our methodology prioritized 25 species, 24 from the BGVCOL (i.e., potato, tomato, tree tomato, Pineapple, cocoa, papaya, and yacon) and one from the NCB (i.e., coffee). Within prioritized BGVCOL, we identified two groups. The first group comprises taxonomic groups within the *Solanum* genus (i.e., potato, tomato, and tree tomato) and cocoa. This group has more than 200 accessions conserved per species, including wild relatives. In contrast, the second group comprises NUS crops, Pineapple, papaya, and yacon. They have few accessions within the BGVCOL, only one representative species within each taxon, and no wild relatives conserved.

Due to the significant differences between the two groups, we suggest different future actions for research. In the case of the first group, we suggest continuing to support the phenotypic and genomic diversity analysis as a base for strengthening breeding programs within Colombia. Below, we detail the research results associated with these species and future research actions.

In the case of the potato collection conserved in the BGVCOL, 809 potato accessions from the andigenum group (diploids and tetraploids) have a genomic characterization. The analysis demonstrated a highly diverse germplasm collection at the phenotypic and genomic levels (Berdugo-Cely et al. 2017). This diversity is promissory for breeding programs. Currently, we are completing molecular characterization of most of the landraces accessions, exploring historical phenotypic data to associate traits, and defining a core collection. Future research activities included using specific accessions for breeding programs to find resistance to certain diseases.

The cocoa collection conserved in the BGVCOL has 565 accessions characterized at the genomic level. The analysis demonstrated a high genetic diversity among groups, supporting that some genetic groups originated in Colombia, which probably is a center of origin for this crop (Osorio-Guarín et al. 2017). Moreover, this collection has association studies that support a breeding program (Osorio-Guarín et al. 2020; Rodriguez-Medina et al. 2019). In addition, researchers from Agrosavia conducted two expeditions in the upper Amazon and the Pacific, searching for wild cacao relatives. Future research actions are associated with the phenotypic and genotypic characterization of wild relatives and with the strength of wild relatives’ research and conservation (González-Orozco et al. 2020).

In the cases of tomato and tree tomato, we should follow the same path previously mentioned. However, they are behind in research goals. Efforts to characterize the accessions for morphological traits are at least thirty years old, and there is no molecular characterization of these collections conserved in the BGVCOL. Therefore, the high-priority status for those species collections is an opportunity to apply for grants associated with phenotypic and genomic characterization. Fortunately, worldwide efforts to characterize tomato genetics, including 100 genomes (Alonge et al. 2020) and the pan-genome (Gao et al. 2019), will facilitate this path.

In contrast with the first group of high-prioritized PGRFA, the future research goals and funds are pretty different for the second group of high-prioritized that correspond to NUS. Papaya, Pineapple, and Yacon have 110, 72, and 2 accessions, respectively, conserved within the BGVCOL. Even inside the BGVCOL, these NUS are underrepresented based on the number of accessions. Moreover, no wild relatives are conserved. In these cases, we suggest reactivating their research by designing fieldwork to identify and collect samples across the country. We encourage different research centers and academic institutions (national or international) to work collaboratively to determine the crops and wild relatives’ distribution in the country to plan expeditions and protect these genetic resources.

Finally, the results showed one prioritized species of coffee from the NCB group (70 PGRFA never conserved in the BGVCOL). Fortunately, CENICAFE (http://www.cenicafe.org) (a Colombian research institution focused on coffee) conserves 1031 accessions from 10 species, with a broad representation of Coffea arabica (85%). At CENICAFE, the bank characterizes the collection to support the breeding program (Alvarado 2022). Nevertheless, we want to highlight the importance of a national research plan for species because of its economic benefits for the country and its potential vulnerability in the face of global warming (DaMatta et al. 2019; Icaro 2014).

### Future research initiatives to improve the index

This study represents the first attempt to align the Colombian plant germplasm bank’s ex situ conservation mission with the SDGs and determine how to allocate the limited budget for Plant Genetic Resources for Food and Agriculture (PGRFA) in a megadiverse country. We selected variables and pillars that used a single data point for each 345 PGRFA and successfully sorted them into high, middle, or low priority using fuzzy logic. However, we also identified several opportunities to improve this methodological effort in the future. They are: (1) add other external species not considered in the 70 PGRFA from the NCB group. (2) for some variables, instead of having a single data per PGRFA, include the diversity across accessions in the analysis. (3) include a new pillar associated with climate actions (13th SDG), and (4) estimate variables for the cost and management of ex situ conservation across collections.

One of the most exciting perspectives to improve the index is to extend the focus on other PGRFA from the NCB group. The current study focused on 70 external PGRFA that appear in several government lists. However, none of the NCB group species had a high score for solving food security, and only one resulted prioritized for economic benefits (i.e., coffee). We hypothesize that the 70 PGRFA from Government lists that conform to the NCB group resulted biased toward external and non-vulnerable PGRFA, which are not necessarily the best options to reduce hunger and poverty. Therefore, it is imperative to update the Government lists and identify wild relatives from PGRFA already conserved in the BGVCOL. Fortunately, as far as we know, three recent studies are moving the focus on those local, underutilized PGRFA and wild relatives not considered before. Unsurprisingly, these studies untangled astonishing plant native biodiversity that deserves future analysis because of their potential for economic benefits and national food security (Gori et al. 2022; Diago and García 2021; Torres-Morales et al. 2021). Moreover, they suggest that this updated external PGRFA list far exceeds previous studies that only reported between 50 and 167 PGRFA, as well as the importance of continuing exploring an ethnobotanical perspective (Pérez-Arbeláez 1978; Romero-Castañeda 1991; Gori et al. 2022; Diago and García 2021).

The first study focused on Bogota’s herb and aromatics market on traditional medicine and rituals. The transdisciplinary analysis found 391 species, 201 of them native. The study prioritized 80 species by interviewing farmers, foragers, and vendors and registered the vulnerability status, best storage, recipes, and uses for food and pharmaceutical industries (Torres-Morales et al. 2021). The second study focused on wild edible fruits from Colombia with high potential for economic benefits and food security. Combining literature review with herbarium databases, they found 706 species, 45 endemics to Colombia. Moreover, 613 are exclusively wild, and 90 are wild or cultivated (Diago and García 2021). Finally, the most recent study of edible plants with a biogeographic perspective found 3805 species, 662 cultivated, and 158 natives (Gori et al. 2022).

Another topic for improving the index is to include the diversity across accessions in the analysis instead of having a single data per PGRFA for certain variables. For example, the characterization of PGRFA conserved in several germplasm banks has demonstrated that there is broad variation in the nutritional contribution variable (food security pillar) (Calliope et al. 2018; Serrano et al. 2017). This phenotyping process is costly for all genebanks independently of the budget available because it requires growing all the accessions in the field or a selected group that represent all the genetic diversity (i.e., core collection or mini-core collection) (Engels and Ebert 2021). Thus, starting with those PGRFA that resulted in high priority in this study, we could design projects that allowed us to characterize those informative traits of agronomic interest at the accession level and, eventually, start to include this information for a future improved index.

Moreover, we could include new variables or pillars, which is mandatory to have data per accession, which is the case of the climate action, the 13th SDGs. This goal aims to “integrate climate change measures into national policies, strategies, and planning” (United Nations 2022). Our current index version does not contain any pillar associated with measuring the vulnerability or adaptation capacity of the PGRFA in the face of climate change. This study focused on Colombia as a single unit (see the Geographic origin pillar) or as thick cultural divisions (see the variable of cultural regions within the food security pillar) to construct the four pillars index. Moreover, we used a single data for each variable per each PGRFA. Therefore, it was out of the main goal to detail the spatial geographic distribution of each PGRFA accession and divide Colombia into regions or departments for modeling the effect or the adaptation capacity of each 375 PGRFA across climate change scenarios. However, this is undoubtedly a short-term future study in mind. The main problem in designing this analysis is the absence of spatial data at accession level, especially those PGRFA without good passports that omit spatial information within the BGVCOL and externals. However, a potential solution is to use the updated National Statistics Department (DANE) to determine the distribution of crops (Ramirez-Villegas et al. 2012). Also, we could combine the spatial phylogenetic diversity approach and gap analysis already used for some crops and wild relatives to identify prioritized PGRFA for ex situ conservation and global climate refugia for PGRFA already conserved in the BGVCOL and externals (González-Orozco et al. 2021, 2022). Once we complete this analysis and identify their potential and limitations, we can create an index to sort the PGRFA for climate change vulnerability or adaptation (Jarvis et al. 2008). Then, we can join this climate change pillar with the current four-pillar index to make informed decisions about research investing of prioritized PGRFA and designing in situ/*on-farm* conservation with communities across identified refugia areas (González-Orozco et al. 2021, 2022).

Finally, we expect the index to be associated with internal management decisions within the BGVCOL related to the cost of conservation, regeneration, and updated documentation, including the open-access database constructed in this study. Despite the importance of this economic analysis, we do not yet have any analysis associated with the cost-benefit of ex situ conservation in the country. There are some clues from calculations from other germplasm banks. These previous analyses included all the management steps, the introduction process (i.e., regeneration, testing of viability and health), the annual increase cost for long-term storage, the cost of regeneration and viability test that occurred periodically (every 20–30 years), and the cost of distribution. Thus, across 11 germplasm banks from the CGIAR system, the cost for storage per accession per year for most species is $1.50. However, for species with cross-pollination as maize, it is $2.16, and for in vitro conservation as cassava is $11.98. Also, for species that require repeated regeneration, the cost increases. For example, the cost per *accession*/*year* for forages is $89.35, wild rice is $68.76, chickpeas is $15.48, and sorghum is $14.66 (Koo et al. 2003). Due to the cost of ex situ conservation changes depending on the biology of the PGRFA, another critical pillar criterion for the Colombian germplasm bank that currently conserves 275 PGRFA should be the cost of ex situ long-term conservation based on the periodicity of regeneration and viability tests (Koo et al. 2003; Gepts 2006). Thus, a ranking of PGRFA based on the conservation cost pillar could be beneficial. First, it could convince stakeholders about the national genebank investment as insurance for the country as a responsible action for the future (Gepts 2006; Perrings 1995). Second, these economic evaluations could help understand other cost-effective methodologies for long-germ conservation for certain PGRFA (i.e., cryopreservation instead of *in-field* conservation) (Pence et al. 2020; Li and Pritchard 2009). Finally, it would help place the ex situ conservation and national germplasm bank research as a fundamental investment in the bioeconomy grants currently promoted by the Science Ministry research goals (Marqueź et al. 2020; Hanley and Perrings 2019).

## Conclusions

The main problem in the PGRFA ex situ conservation research is the lack of funding. In the case of megadiverse countries such as Colombia, the decision about how to invest money is even more critical. Therefore, developing a tool for deciding what conserved PGRFA is a priority for research investment aligned with solving both sustainable uses and social problems is imperative. Here we developed for the first time a data-driving index based on four pillars of information to rank 345 Colombian PGRFA aligned with the sustainable goals of zero hunger and no poverty. They include geographic origin, vulnerability status, economic benefits, and food security importance. The four-pillar index used fuzzy logic and successfully ranked each PGRFA in three groups: high, middle, and low priority. The index found 25 PGRFA in high priority. Of them, 24 are already ex situ collections from the Colombian germplasm bank (BGVCOL), and one was external (i.e., not currently conserved in the BGVCOL). Our methodology demonstrated several advantages: the data used to construct the index came from open-access databases that we also summarized in a single open-access database. Also, we used a data-driven approach independent of the bank scientists’ preferences and biases aligned with two sustainable goals. The approach described aligned with two of the four missions recently defined by the Colombian government to address the country’s biggest challenges: (1) Bioeconomy, natural ecosystems, and sustainable territories, and (2) Right to Food Minciencias Ministerio de ciencia t.e.i (2022).

Likewise, classifying species into three prioritizing categories simplified the information for non-scientific training stakeholders and politicians who usually decide how to invest research funds. Besides, the methodology identified the most significant information gaps for native PGRFA and wild relatives and the possibilities to impute information when information of the closest PGRFA is available. Finally, the index is versatile either for adding in the future more PGRFA (especially for those not currently conserved in the BGVCOL) or for adding more pillars associated with other sustainable goals, such as vulnerability or adaptation to climate change. These advantages make this index a flexible decision tool to implement in other national genebanks that lack funding but are interested in identifying key PGRFA for aligning the ex situ conservation mission with sustainable goals. Acknowledging that many of the above problems are in Latin America, this study is also available in Spanish to promote a discussion about this tool across regional genebanks (File S1).

## Supplementary Information

Below is the link to the electronic supplementary material.

Supplementary file1 (XLSX 43 KB)

Supplementary file2 (XLSX 24 KB)

Supplementary file3 (PDF 6656 kb)

Supplementary file4 (PDF 402 kb)

## Data Availability

We built a web interface to give access to the data presented here. The datasets used during the current study are available in the repository http://indicebgv.agrosavia.co:3997/. The data set used and the scripts used for the priority index in R are available at https://github.com/phrh/BGVCOLPriorityIndex.
