# Supplementary material for: Prioritizing Colombian plant genetic resources for investment in research using indicators about the geographic origin, vulnerability status, economic benefits, and food security importance
Source: Biodivers Conserv. 2023 May 19;32(7):2221–61. doi: 10.1007/s10531-023-02599-7 (PMC10195663; doi:10.1007/s10531-023-02599-7)
Supplement: Supplementary file 3 — Supplementary file3 (PDF 6656 kb) [file 10531_2023_2599_MOESM3_ESM.pdf]

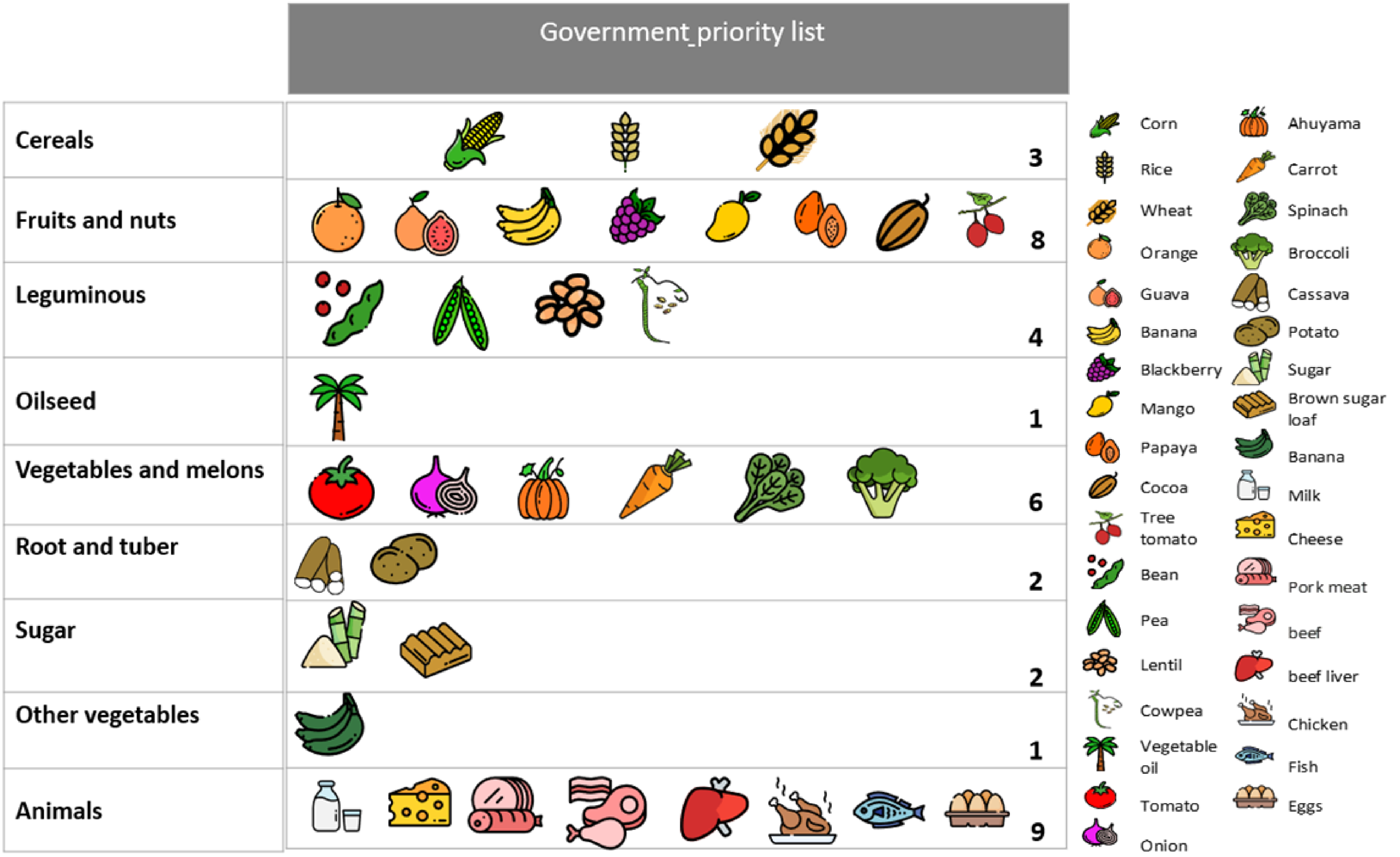

Figure S1. The prioritized list of plant genetic resources for food and agriculture (PGRFA) and animal products. The Colombian government developed this list to guarantee its production and supply policies to improve the stable consumption in the Colombian population's diet. A unique icon represents each species or product, showing the separation by FAO classification of food groups.

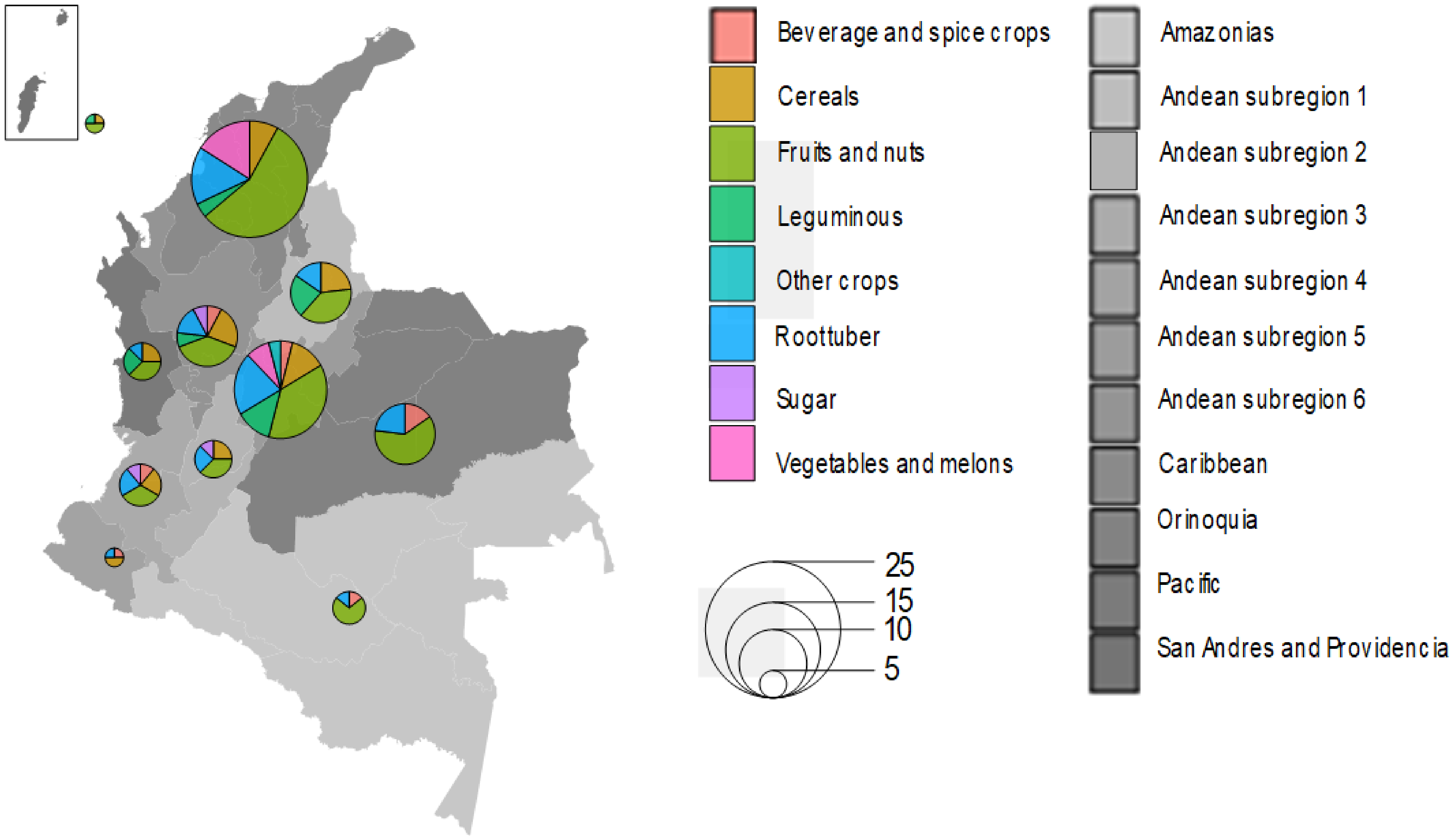

Figure S2. The Colombian map shows 11 geographic regions with different levels of grey. Within each region is a pie with the number of Plant Genetic Resources for Food and Agriculture (PGRFA) essential for each region's food tradition, separating them by different colors representing eight FAO food groups. The size of the pie is equivalent to the number of PGRFA included.

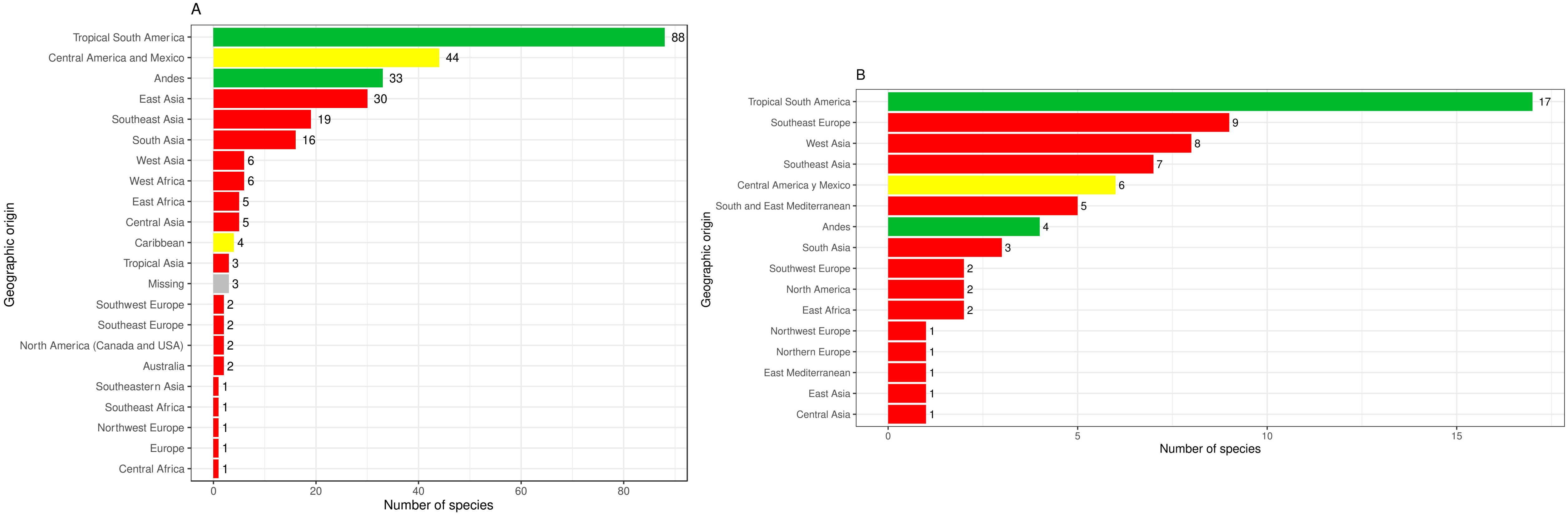

Figure S3. The geographic origin of the 345 plant genetic resources for food and agriculture (PGRFA) across 26 world regions. (A) Two hundred seventy-five conserved in the National Plant Germplasm Bank (BGVCOL group), (B) 70 are not currently conserved in the BGVCOL (i.e., NCB group). The bar shows the region's name, indicating the number of PGRFA with origin in each region in blue. The bar's color represents the three prioritizing categories used in this study. In green, the local origin with high priority includes Tropical South America and Andes, two regions where Colombia is localized. The close origin with middle priority includes Central America, Mexico, and the Caribbean in yellow. Finally, in red, the distant regions with low priority.

Figure S4. The classification of 275 PGRFA conserved in the National Plant Germplasm Bank (BGVCOL) by their vulnerability state. (A) The percentage of vulnerability state of the PGRFA analyzed by FAO food categories: Not evaluated (grey), Minor concern (yellow), and threatened (orange). (B) The number of PGRFA without vulnerability information by geographic regions in the world.

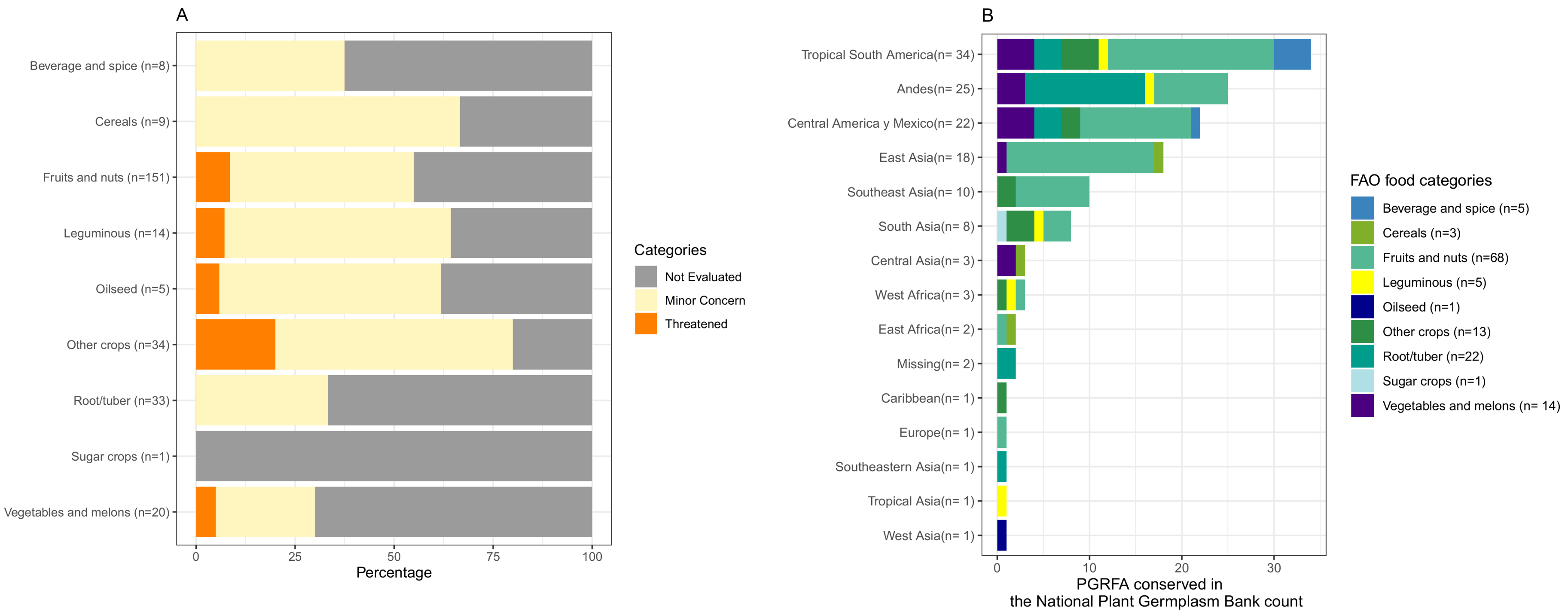

Figure S5. The classification of 70 PGRFA not conserved in the National Plant Germplasm Bank (i.e., NCB group) by their vulnerability state. (A) The percentage of vulnerability state of the PGRFA analyzed by FAO food categories: Not evaluated (grey), Minor concern (yellow), and threatened (orange). (B) The number of PGRFA without vulnerability information by geographic regions in the world.

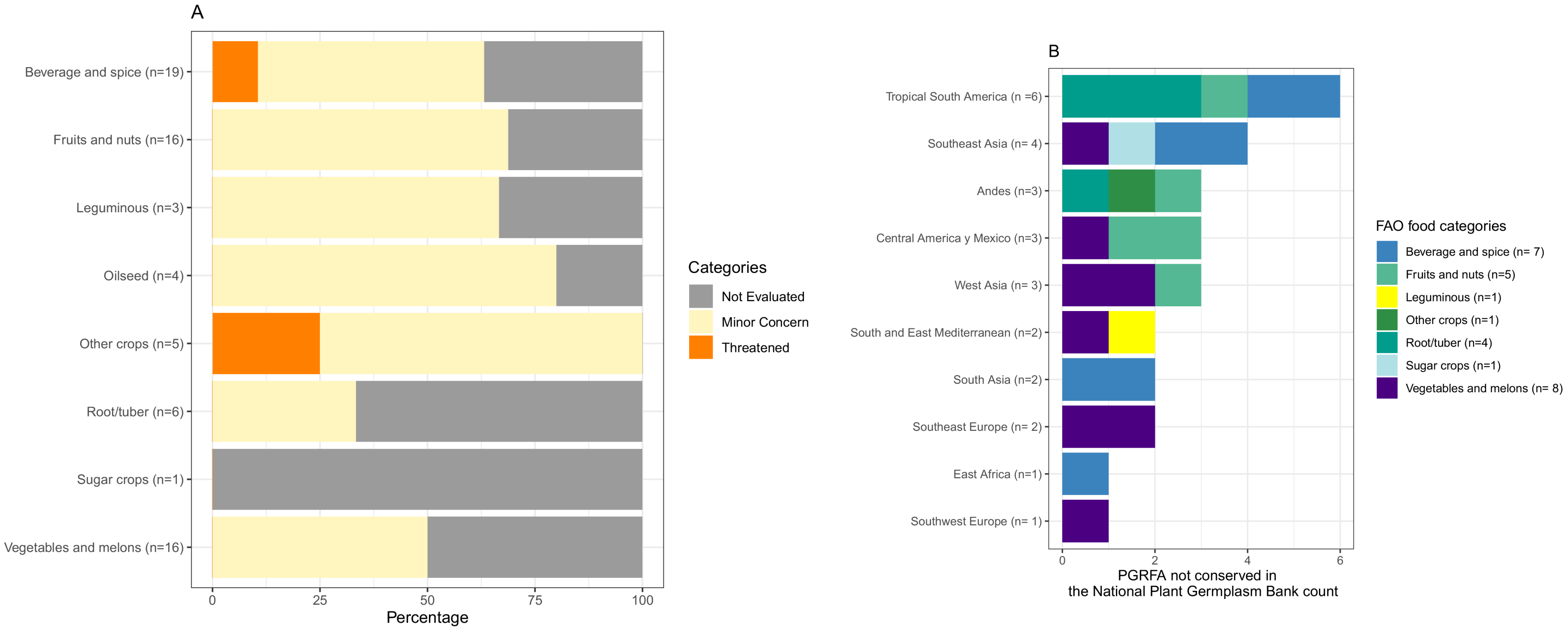

Figure S6. Boxplots indicate the median and variation of four variables considered in the economic benefits pillar for the PGRFA conserved in the National Plant Germplasm Bank (BGVCOL) separated by FAO food categories with the number of species within each category (n). (A) Income (thousand USD ha^{-1}). (B) Lafay index. (C) Municipality coverage (%). (D) Yield (t ha^{-1}). One USD = 3,418 COP average annual over the last five years.

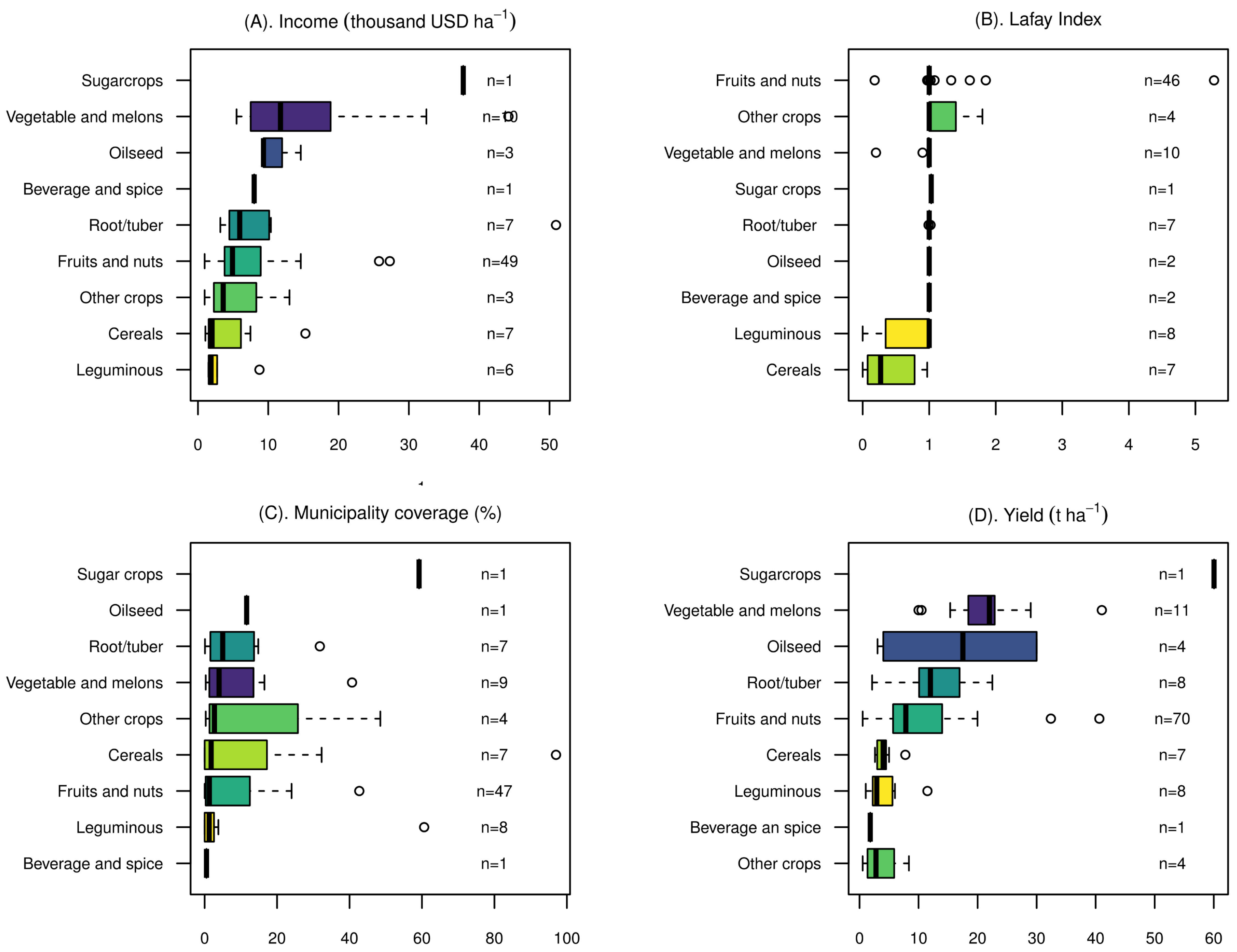

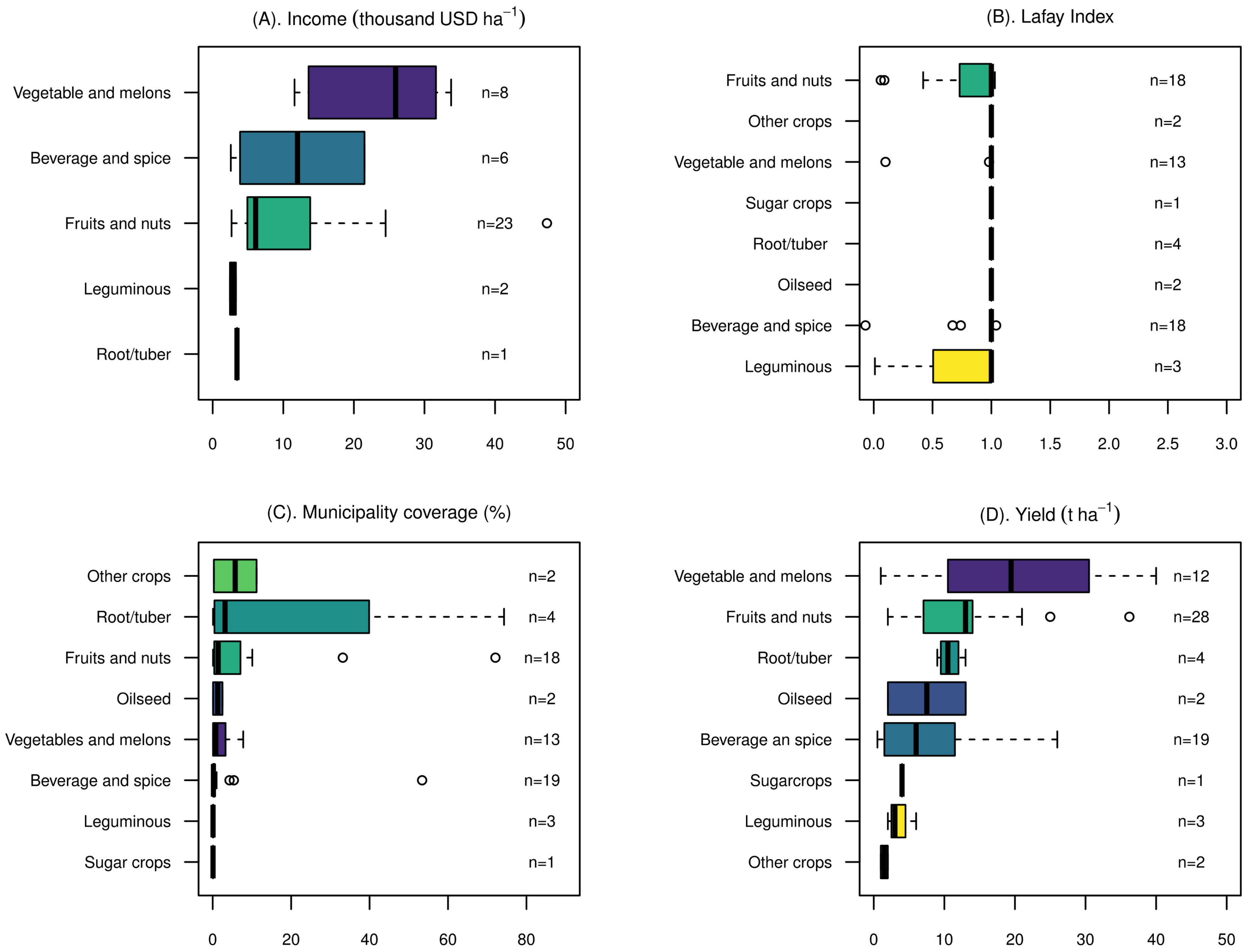

Figure S7. Boxplots indicate the median and variation of four variables considered in the economic benefits pillar for the PGRFA not conserved in the National Plant Germplasm Bank (NCB group) separated by FAO food categories with the number of species within each category (n). (A) Income (thousand USD ha^{-1}). (B) Lafay index. (C) Municipality coverage (%). (D) Yield (t ha^{-1}). One USD = 3,418 COP average annual over the last five years.

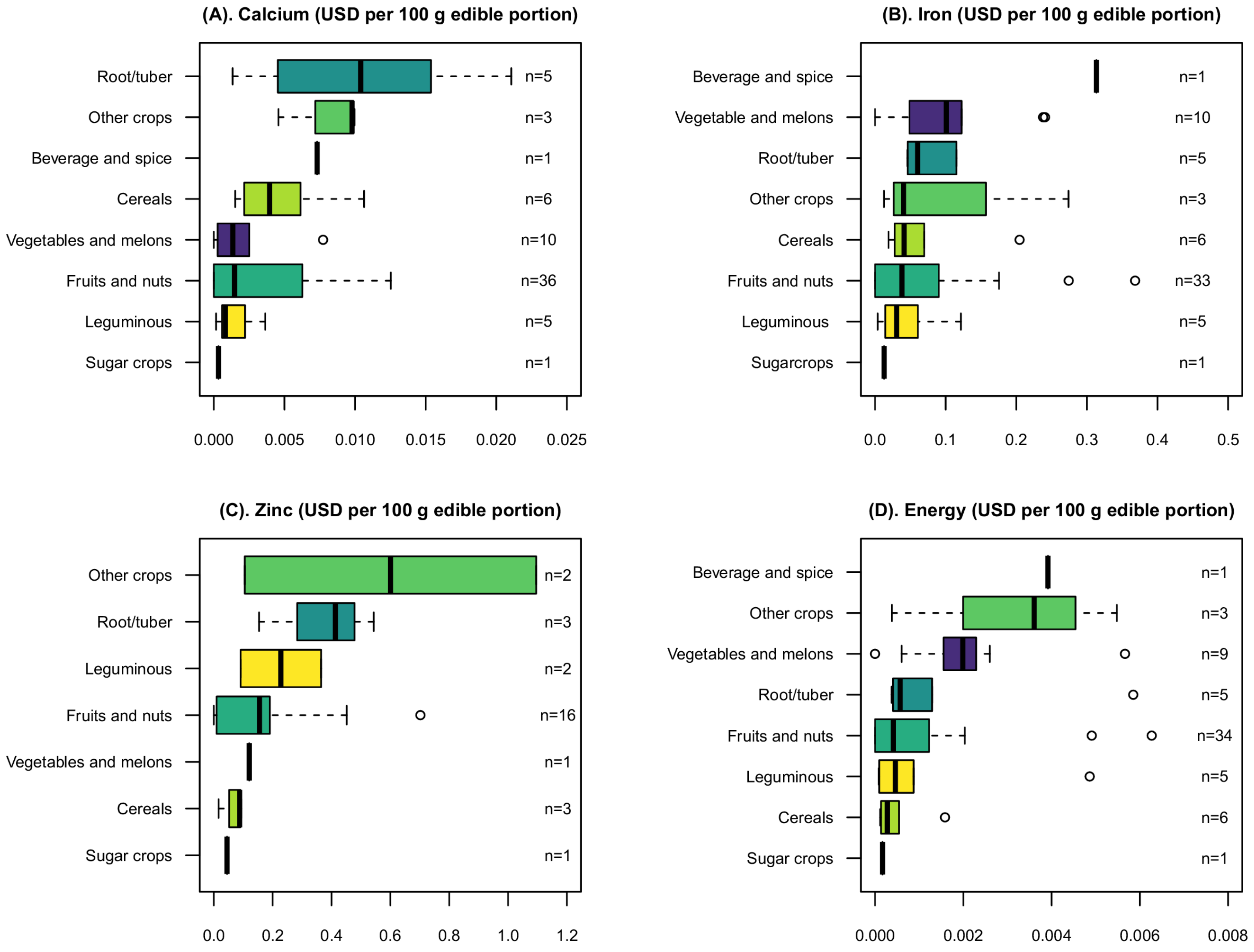

Figure S8. Boxplots indicate the median and variation of the affordability in USD per 100 g edible portion for (A) Calcium-Ca, (B) Iron-Fe, (C) Zinc-Zn, and (D) Energy separating the PGRFA conserved in the National Plant Germplasm Bank (BGVCOL) by FAO food categories indicates the number of species (n). One USD = 3,418 COP average annual over the last five years.

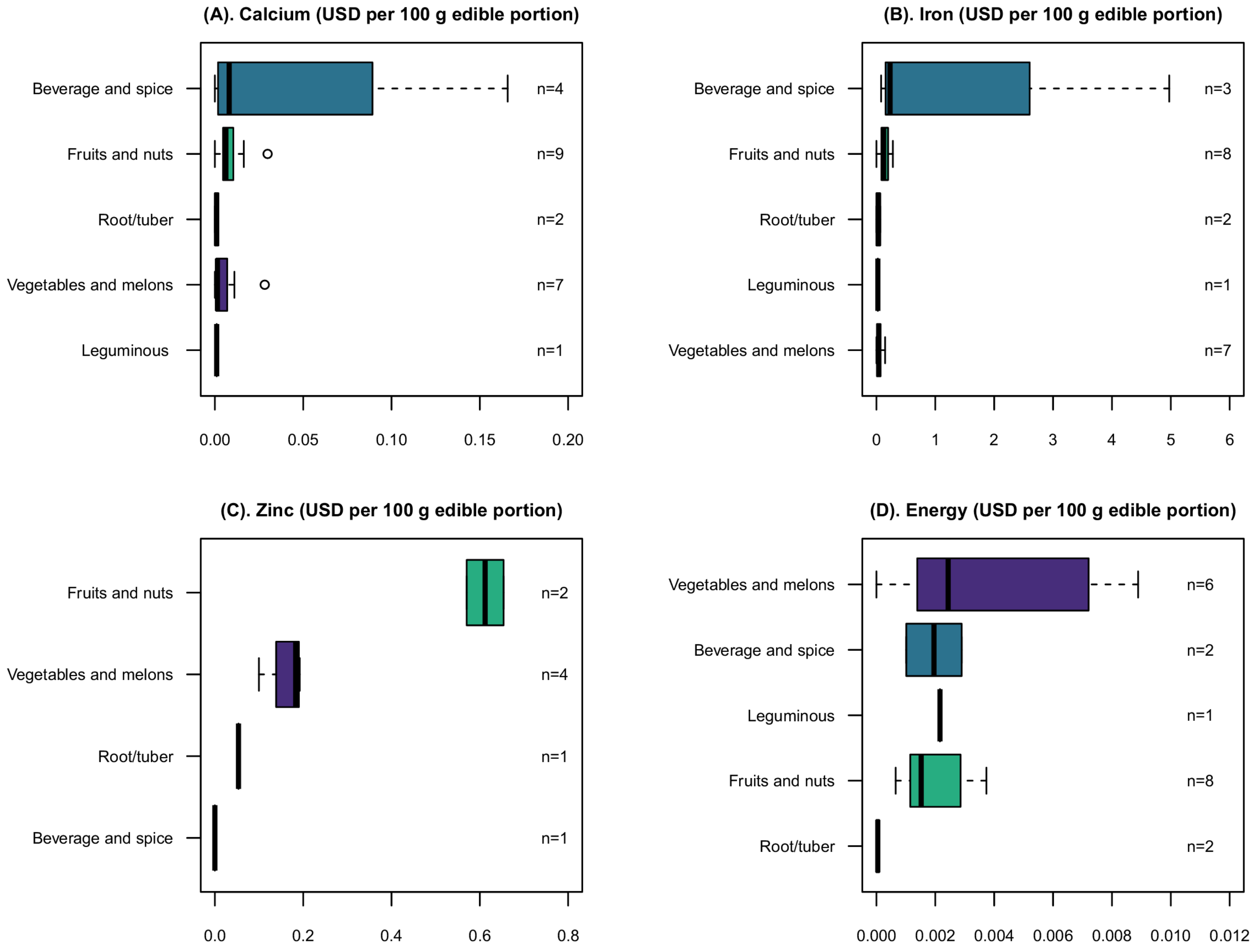

Figure S9. Boxplots indicate the median and variation of the affordability in USD per 100 g edible portion for (A) Calcium-Ca,(B) Iron-Fe, (C) Zinc-Zn, and (D) Energy separating the PGRFA not currently conserved in the National Plant Germplasm Bank (i.e., NCB group) by FAO food categories indicates the number of species (n). One USD = 3,418 COP average annual over the last five years.

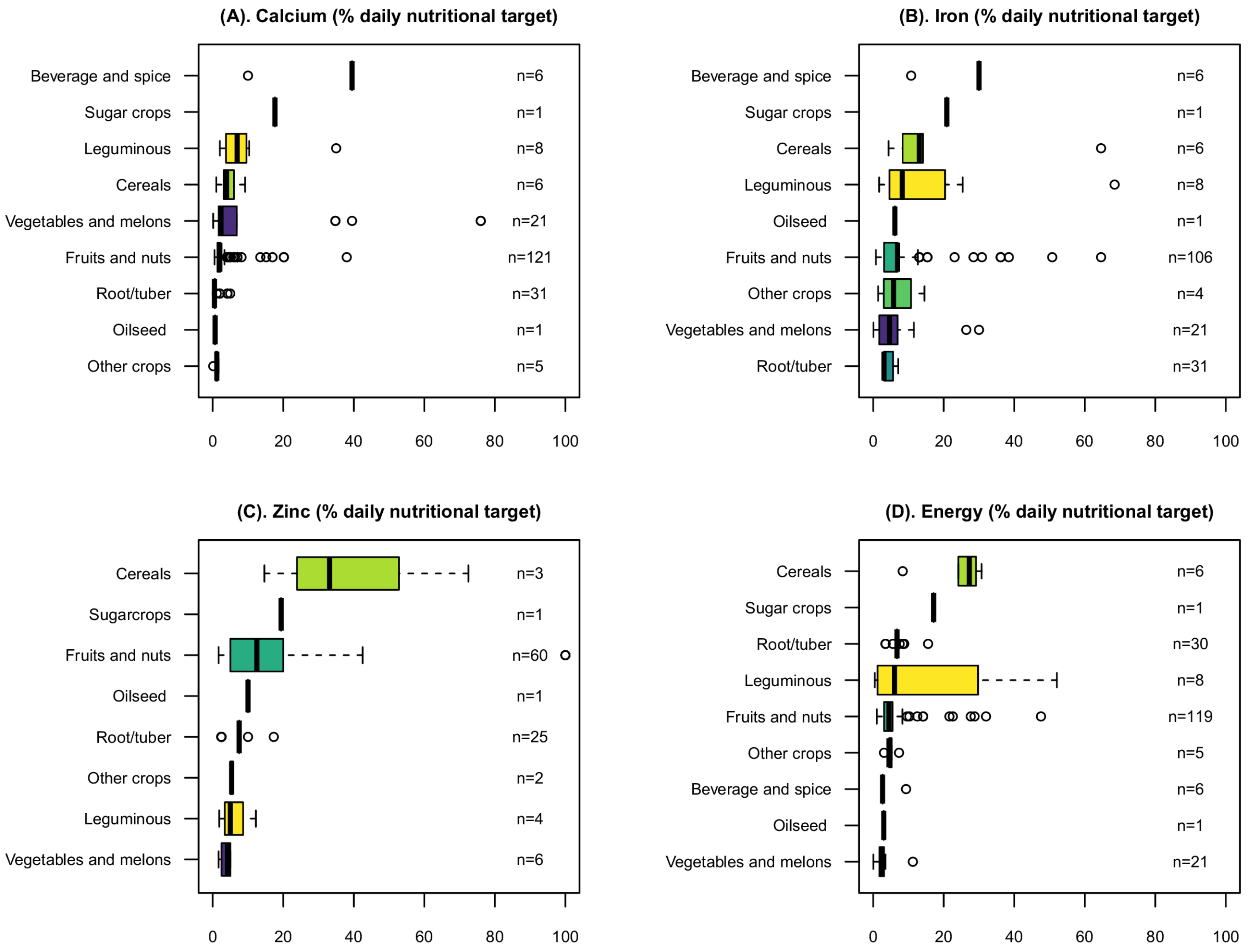

Figure S10. Boxplots indicate the median and variation of the percentage (%) of the daily nutritional target for (A) Calcium-Ca,(B) Iron-Fe, (C) Zinc-Zn, and (D) Energy, separating the PGRFA conserved in the National Plant Germplasm Bank (BGVCOL) by FAO food categories indicates the number of species (n).

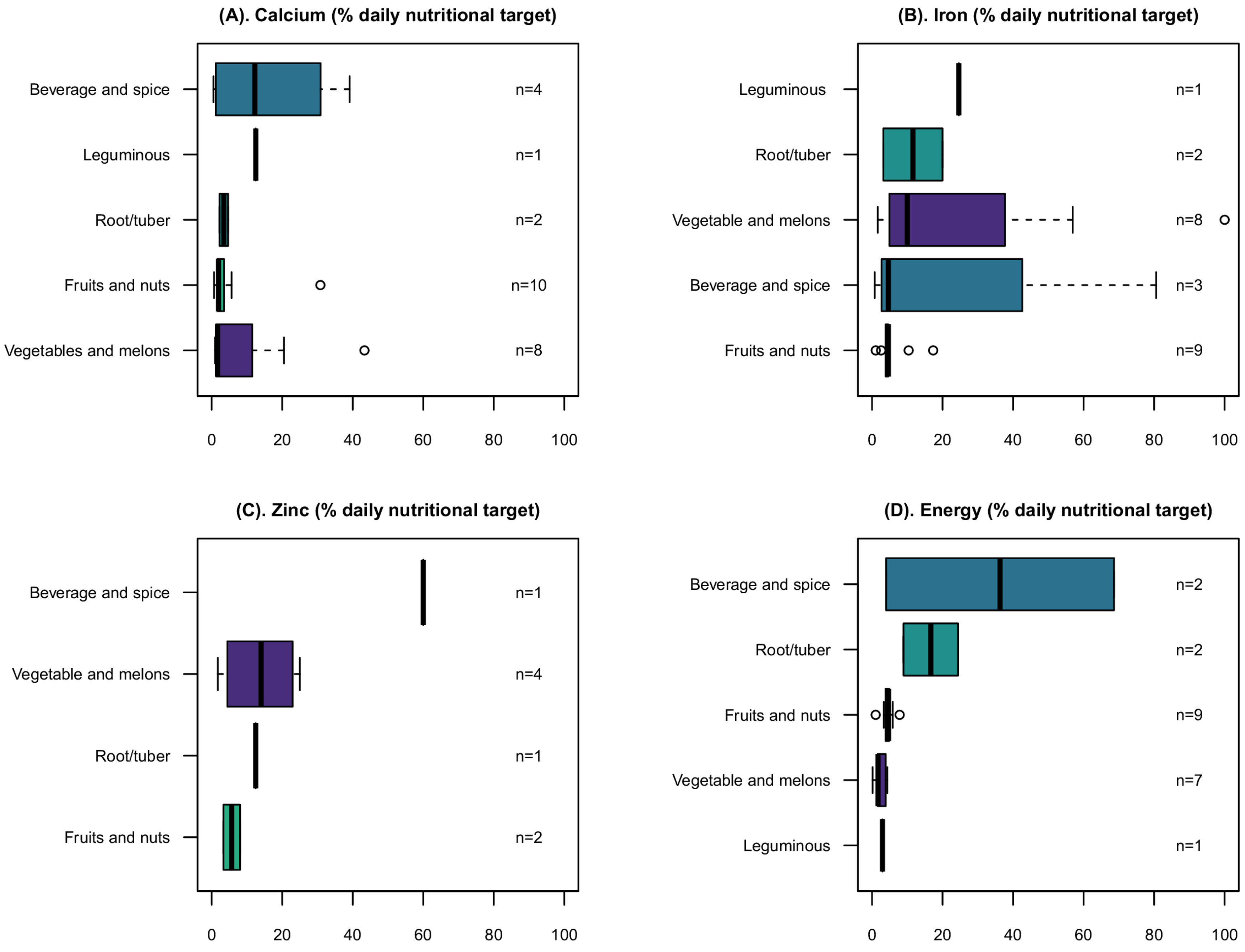

Figure S11. Boxplots indicate the median and variation of the percentage (%) of the daily nutritional target for (A) Calcium-Ca, (B) Iron-Fe, (C) Zinc-Zn, and (D) Energy separating the PGRFA not conserved in the National Plant Germplasm Bank (i.e., NCB group) by FAO food categories indicates the number of species (n).

Figure S12. The PGRFA conserved in the BGVCOL resulted in the high priority category for (A) the Geographic origin pillar and (B) the Vulnerability pillar. The PGRFA are grouped by FAO food categories, indicating the name and the number of species. The icons represent the crop taxon and are the same used in Fig. 4.

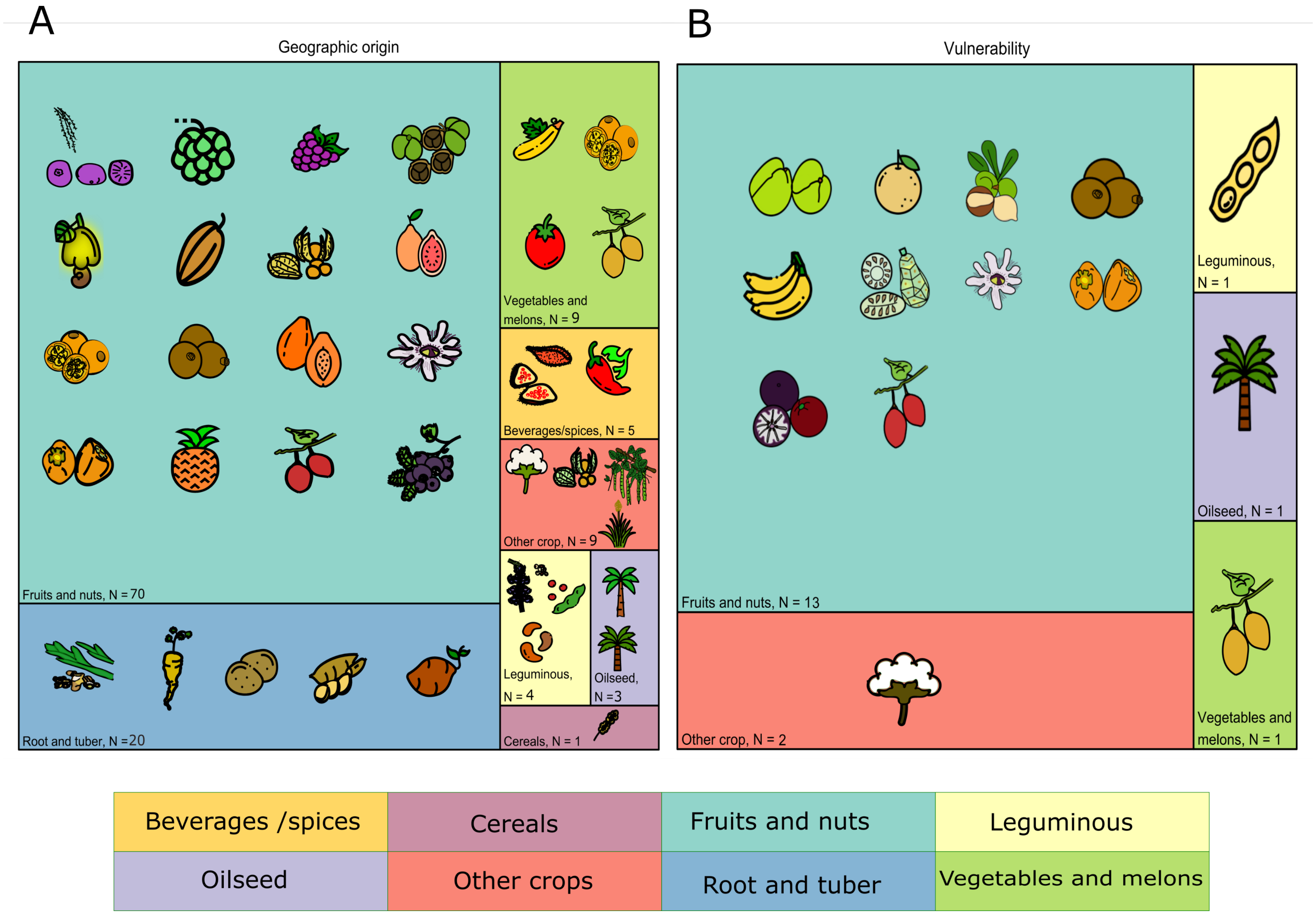

Figure S13. The PGRFA conserved in the BGVCOL resulted in the high priority category for (A) the Economics benefits pillar and (B) the Food security pillar. The PGRFA are grouped by FAO food categories, indicating the name and the number of species. The icons represent the crop taxon.

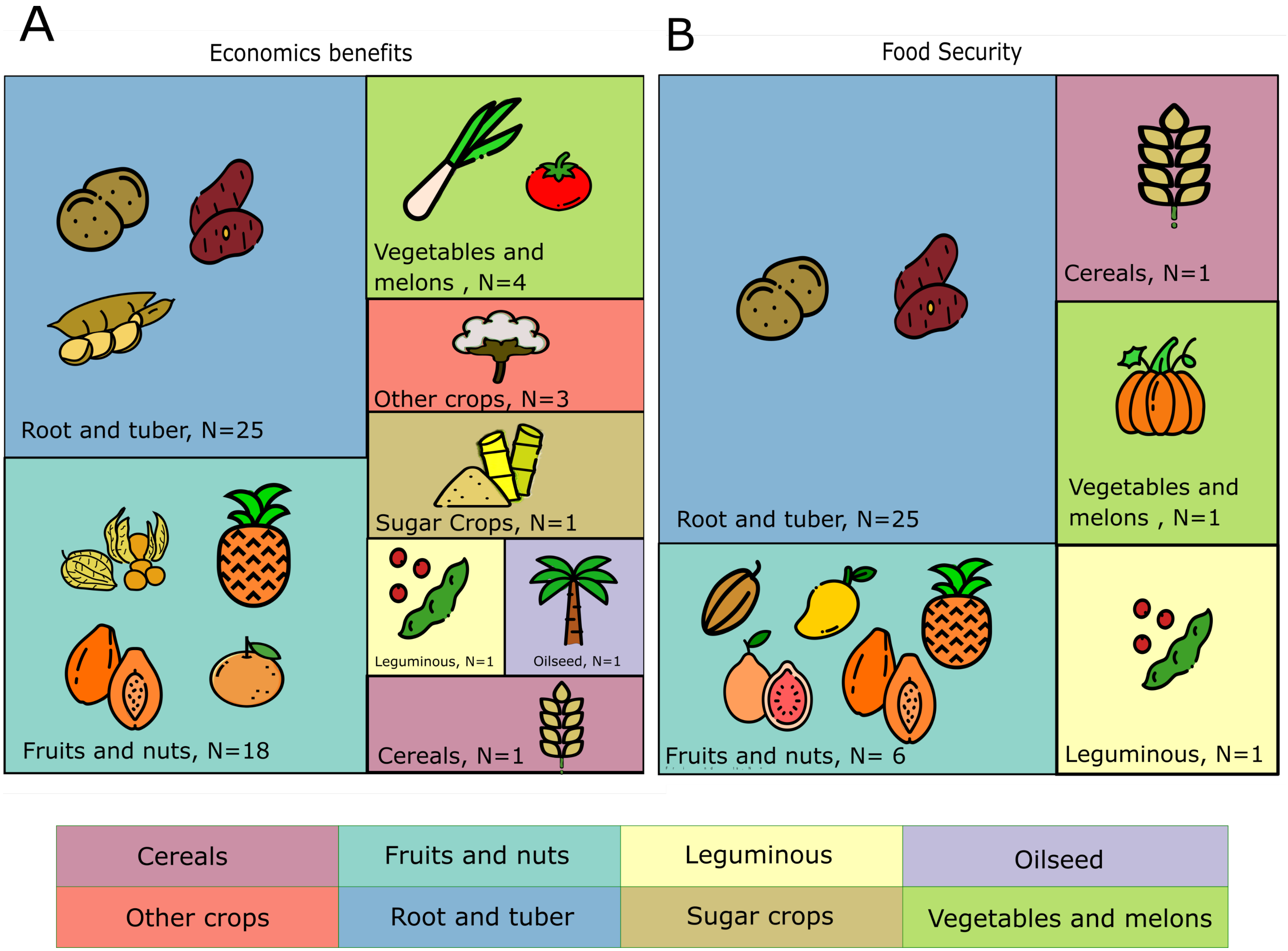

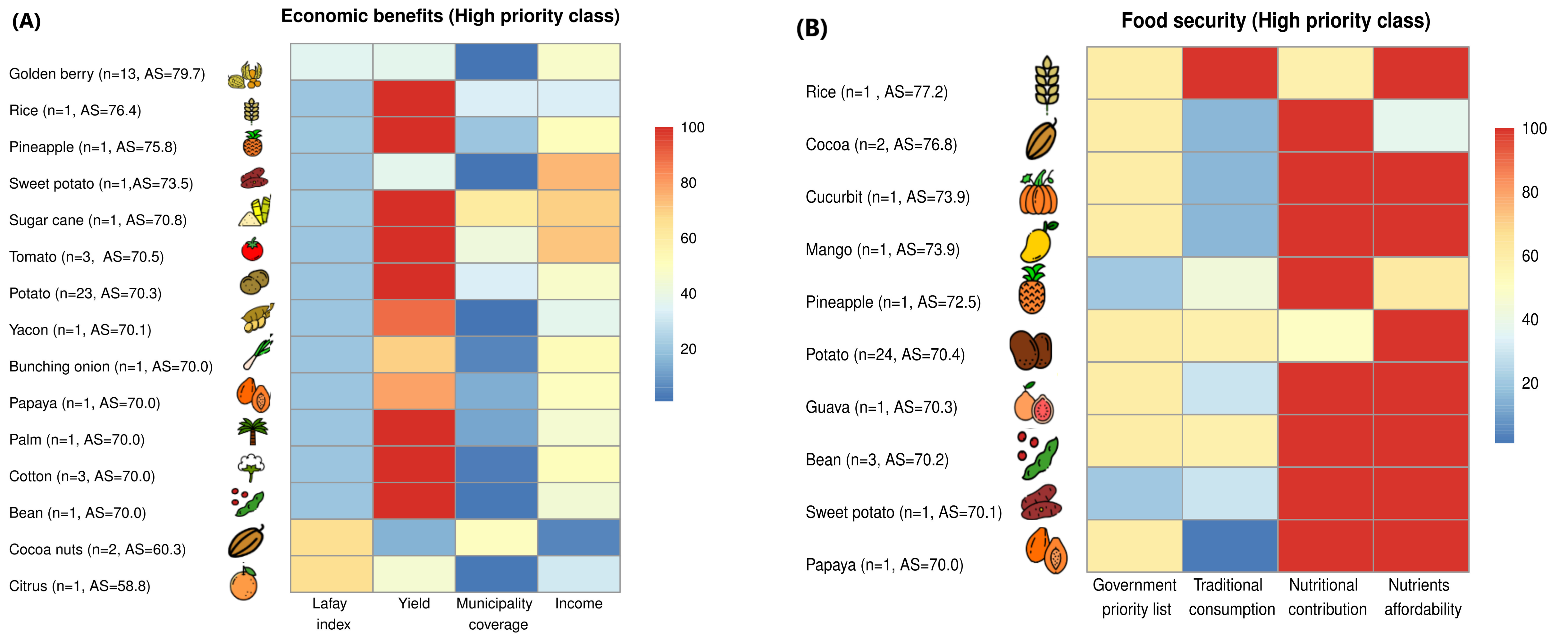

Figure S14. The PGRFA conserved in the National Plant Germplasm Bank (BGVCOL) within the highest priority class for two pillars: (A) The economic benefits pilar with four variables, Lafay index, yield, municipality coverage, and income. (B) The food security pilar with four variables, government priority list, traditional consumption, nutritional contribution, and nutrients affordability. The PGRFA are split by FAO food categories in a unique icon, indicating the common name and the number of species (n), sorted from the highest to the lowest average score (AS) measured as a percentage. The color shows the AS obtained for each variable from 0 (blue) to 100 (red). The icons are the same as used in Fig. 4.
