# Supplementary material for: Prioritizing Colombian plant genetic resources for investment in research using indicators about the geographic origin, vulnerability status, economic benefits, and food security importance
Source: Biodivers Conserv. 2023 May 19;32(7):2221–61. doi: 10.1007/s10531-023-02599-7 (PMC10195663; doi:10.1007/s10531-023-02599-7)
Supplement: Supplementary file 4 — Supplementary file4 (PDF 402 kb) [file 10531_2023_2599_MOESM4_ESM.pdf]

Priorización de los recursos fitogenéticos colombianos para la inversión en investigación utilizando indicadores sobre el origen geográfico, el estatus de vulnerabilidad, los beneficios económicos y la importancia para la seguridad alimentaria

I. Cerón-Souza[1], D. Delgadillo-Duran[1], S.M. Polo-Murcia[1], Z.X. Sarmiento-Naizaque[1] y P.H. Reyes-Herrera[1*]

[1*] CI Tibaitatá, Corporación Colombiana de Investigación Agropecuaria AGROSAVIA, Km 14 vía Mosquera, Bogotá, Colombia.

*Autor para correspondencia. E-mail(s): phreyes@agrosavia.co

Resumen

Los bancos de germoplasma son el depósito más importante de recursos fitogenéticos para la alimentación y la agricultura (RFAA) en todo el mundo. A pesar de su importancia estratégica, los bancos nacionales de germoplasma de los países tropicales megadiversos en desarrollo, como Colombia, cuentan con fondos extremadamente limitados. Por lo tanto, es esencial tomar decisiones estratégicas sobre la inversión en investigación. Aquí, diseñamos un enfoque basado en datos para construir un índice que clasifica los RFAA colombianos en tres grupos: alta, media o baja prioridad, basado en cuatro pilares de información de bases de datos de acceso abierto y alineado con los objetivos sostenibles de no pobreza y cero hambre: Origen geográfico, estatus de vulnerabilidad, beneficios económicos e importancia para la seguridad alimentaria. Utilizamos la lógica difusa para clasificar cada RFAA por cada pilar y calculamos un nivel de certeza para cada variante en caso de ausencia de datos e imputación con la especie más cercana disponible. Analizamos 345 RFAA utilizando el índice, separándolos en dos grupos, 275 ya conservados en el banco de germoplasma colombiano (grupo BGVCOL) y 70 no conservados actualmente en el BGVCOL (grupo NCB). Los RFAA con mayor prioridad son 24 (8,72%) del BGVCOL (15 de papa, tres de tomate, dos de tomates de árbol, piña, cacao, papaya y yacón), y uno (café) del NCB. Los datos obtenidos por cada RFAA se encuentran en una base de datos de libre acceso. Concluimos que esta metodología priorizó exitosamente los RFAA en Colombia y muestra los grandes vacíos de conocimiento para futuras investigaciones así como alternativas para mejorar este índice. La versatilidad de esta metodología podría ser útil en otros bancos de germoplasma con limitaciones presupuestales para la inversión en investigación.

Palabras clave: Colombia, especies de cultivos, conservación *ex situ*, lógica difusa, bancos de genes, bancos de germoplasma.

1 Introducción

Los puntos calientes de la biodiversidad están ubicados principalmente zonas tropicales y subtropicales [1]. Estas áreas también coinciden con el 28% de los centros de domesticación de cultivos [2]. De estas zonas, los países latinoamericanos son esenciales. Incluyen los centros de diversidad mesoamericanos y andinos definidos por Vavilov, donde varias especies de cultivos son críticas para la seguridad alimentaria mundial, como el maíz, la papa y el frijol [3]. Esta situación favorable debería implicar el uso sostenible de la biodiversidad para bienes y servicios entre los países latinoamericanos. Sin embargo, esto todavía no es así. En 2015, el hambre y la malnutrición afectaron a cerca de 34 millones de personas en América Latina y el Caribe, contribuyendo al 5,5% a nivel mundial [4]. Además, uno de cada cinco vive en la pobreza crónica dentro de esta región [5]. Esta situación parece agravarse debido a la pandemia de COVID-19. El bloqueo cerró los programas de alimentación para los pobres en América Latina y el Caribe, afectando principalmente a las mujeres, los niños y los inmigrantes [6, 7] . Además, el cierre impactó en la demanda y la oferta de alimentos, ambas asociadas a la seguridad alimentaria nacional [6, 8, 9].

Antes de la pandemia, los países latinoamericanos (excepto Brasil) invertían menos del 1% del PIB en investigación científica, lo que corresponde a menos que las economías de ingresos medios-bajos del mundo [10]. Sin embargo, la pandemia de COVID-19 ha mostrado la importancia de la salud y la agricultura para fortalecer la seguridad alimentaria [11, 12]. Por lo tanto, el actual contexto mundial es una oportunidad para reexaminar la importancia extrema de los países latinoamericanos en el apoyo a la recolección bien planificada, la conservación *ex situ* y la investigación de los recursos fitogenéticos para la alimentación y la agricultura (RFAA). En última instancia, esta inversión supone un seguro para evitar el coste social de la pérdida de biodiversidad [2, 13].

Los RFAA incluyen todas las plantas con valor real o potencial para la producción agrícola que los obtentores utilizan para desarrollar nuevas variedades de cultivos. Estos materiales abarcan los cultivos, los parientes silvestres de los cultivos, los cultivares antiguos, las razas autóctonas y los cultivares tradicionales obtenidos por los agricultores mediante selección natural o artificial. Por lo tanto, es la base de la supervivencia de la humanidad porque genera alimentos, combustible y fibras [14]. Actualmente, el 68,7% de los suministros nacionales de alimentos son cultivos extranjeros, y ningún país es completamente autosuficiente [15]. Esta interdependencia entre países es el resultado de la historia de las migraciones humanas, que ha promovido a lo largo de los siglos el intercambio de razas y variedades autóctonas [14]. Así, los beneficios económicos del descubrimiento y el uso de los RFAA superan ampliamente la inversión en su búsqueda y conservación dentro y entre países [16].

Lamentablemente, los RFAA son especialmente susceptibles a la disminución de la variación genética (es decir, a la erosión genética). La razón principal es la frecuente sustitución en todo el mundo de las variedades locales por variedades modernas y la agricultura intensiva que promueve la uniformidad genética, la agricultura insostenible y el abandono de los RFAA locales. Además, las nuevas plagas, las enfermedades y los factores ambientales como el calentamiento global, la urbanización y la destrucción de la tierra son otras de las principales amenazas para los RFAA [17]. Muchos países combinan estrategias de conservación *in situ* (en su hábitat natural) y *ex situ* (fuera de su hábitat natural) para los RFAA. El método *ex situ* implica la conservación a largo plazo de los RFAA, principalmente en los bancos de germoplasma, también conocidos como bancos de genes. Sin embargo, desde que se establecieron los bancos de germoplasma en diferentes países, la principal preocupación ha sido la escasez de fondos para mantener programas de investigación a largo plazo, instalaciones adecuadas para la conservación a largo plazo de los RFAA y personal especializado [16]. Actualmente, las Naciones Unidas generan 17 objetivos de desarrollo sostenible (ODS), conocidos como objetivos globales enfocados a mejorar la paz y la prosperidad de la humanidad para el año 2030 [18]. De estos objetivos, el objetivo 1: no a la pobreza y el objetivo 2: cero hambre, representan oportunidades para alinear la misión de los bancos nacionales de germoplasma con las partes interesadas para aumentar el apoyo financiero para la conservación e investigación de los RFAA en diferentes países, incluyendo Colombia [19].

Colombia está clasificado como un país "megadiverso" que alberga alrededor del 10% de la biodiversidad mundial [20]. Desde 1995, Colombia forma parte del Convenio de Diversidad Biológica (CDB), una iniciativa global que promueve la protección de la diversidad de la vida y su uso sostenible (CDB 2021). En Colombia, el Banco Nacional de Germoplasma Vegetal (BGVCOL) forma parte de este esfuerzo público de conservación *ex situ* que comenzó en 1994. Sin embargo, la recolección de RFAA en todo el país se inició entre los años 20 y 50 como parte de un programa nacional de mejoramiento genético apoyado por la Fundación Rockefeller y la cooperación técnica de Estados Unidos en América Latina, generando los primeros bancos de semillas [21]. En 1996 el gobierno impulsó el sistema nacional de bancos de germoplasma con un presupuesto anual para la conservación de todos los RFAA conservados en el BGVCOL además de los bancos nacionales de animales y microorganismos. Además, desde 2018, el Ministro de Agricultura y Desarrollo Rural ha delegado la función administrativa de los bancos nacionales de germoplasma a Agrosavia (Corporación Colombiana de Investigación Agropecuaria), incluyendo un acuerdo de entrega de material (ATM) para adquirir y entregar RFAA de acuerdo con las políticas internas nacionales y de Agrosavia en el marco del CDB. Sin embargo, algunos detalles de esta política podrían cambiar en el mediano plazo debido a que el Congreso Nacional ratificó y aprobó recientemente el ingreso al TIRFAA (Tratado Internacional sobre los Recursos Fitogenéticos para la Alimentación y la Agricultura) y ahora está en proceso de aprobación legal por parte de la Corte Constitucional colombiana.

Actualmente, el BGVCOL mantiene más de 30.000 accesiones diferentes de 275 especies y 109 grupos de taxones conservados en tres sistemas: semillas, campo e *in vitro* [21]. Sin embargo, a pesar de la dimensión del BGVCOL, no existe un sistema para priorizar la inversión en investigación para los RFAA ya conservados dentro del BGVCOL, ni un criterio para determinar qué RFAA externos no conservados en el BGVCOL deberían empezar a formar parte de un plan de conservación *ex situ*. La ausencia de información para la biodiversidad aplica para todos los RFAA, y es necesario evaluar inventarios actualizados de la diversidad colombiana, especialmente para plantas silvestres y cultivos huérfanos; nuestro fundamento es que la importancia de los RFAA podría cambiar bajo una revisión de los criterios actuales y convertirse en una prioridad para el esfuerzo nacional de conservación *ex situ*.

De acuerdo con lo anterior, este estudio se centró en la creación de un índice de priorización para llenar este vacío. Este índice utilizó cuatro pilares (es decir, criterios de información) para construir un enfoque basado en datos y en la lógica difusa [22] para una inversión en investigación bien planificada en el futuro en Colombia en dos grupos de RFAA: los actualmente conservados en el BGVCOL y los externos (Fig. 1 y Fig. 2).

Los pilares que proponemos son el origen geográfico, el estatus de vulnerabilidad, los beneficios económicos y la importancia para la seguridad alimentaria. El nivel de endemismo y el estatus de vulnerabilidad se han considerado habitualmente como indicadores de clasificación de especies para su conservación *ex situ* [23-25]. En cambio, hasta donde sabemos, los beneficios económicos y la importancia de la seguridad alimentaria no suelen incluirse como indicadores para clasificar los RFAA y tomar decisiones de inversión en investigación. En este estudio, definimos los beneficios económicos como la ganancia económica para el país por cultivar un determinado RFAA, y utilizamos cuatro variables macroeconómicas para comprender este pilar. Además, definimos la importancia de la seguridad alimentaria como el grado de nutrición de un RFAA comestible para satisfacer las necesidades de seguridad alimentaria de los colombianos, especialmente de los niños de familias rurales. Asimismo, utilizamos cuatro variables para analizarlas en función de los micronutrientes (es decir, calcio, hierro, zinc y energía). Evaluamos estos cuatro pilares juntos por dos razones principales. En primer lugar, pueden generar información crítica asociada a los objetivos de los ODS, como la no pobreza y el hambre cero, como parte de la misión de conservación *ex situ* del BGVCOL [18]. En segundo lugar, Colombia tiene actualmente desafíos muy particulares que necesitan atención urgente en la investigación agrícola nacional. Entre ellos se encuentran el alto riesgo de la producción agrícola ante el calentamiento global [26], el acuerdo de paz firmado en 2016 con un gran enfoque en el desarrollo rural (es decir, la reforma rural integral) pero con retrasos en su implementación [27], y una severa inequidad poblacional exacerbada por la pandemia de COVID-19 que afecta a zonas más rurales [28]. Por lo tanto, nuestro razonamiento es que al identificar los RFAA nativos, vulnerables, rentables y nutritivos, alineamos la misión de conservación *ex situ* a largo plazo del BGVCOL con la investigación de alto impacto para el país. Usando el índice de priorización basado en estos cuatro pilares propuestos aquí, encontramos 24 RFAA de 275 con alta prioridad de investigación conservados en el BGV- COL y sólo un RFAA de alta prioridad de 70 externos. Creemos que este enfoque basado en datos desarrollado en este estudio es adaptable para incluir más RFAA, más pilares y más variables dentro de cada pilar en el futuro. Además, a pesar de que este estudio se centró en Colombia, otros países e instituciones también podrían adoptar esta herramienta para clasificar y priorizar la inversión en la conservación *exsitu* de sus RFAA.

2 Metodología

Este estudio se centró en la generación de un índice de priorización que clasificó 345 RFAA nacionales a partir de dos conjuntos de datos diferentes. El primer conjunto corresponde a 275 RFAA conservados *ex situ* en el BGVCOL (es decir, el grupo BGVCOL) (Fig. 1). El segundo conjunto corresponde a 70 RFAA nunca conservados en el BGVCOL (es decir, el grupo NCB). Estos externos al BGVCOL representan una pequeña porción de todos los RFAA que tiene Colombia como país megadiverso [29]. Sin embargo, los seleccionamos porque han aparecido listados en varias agencias nacionales desde 2013 por su valor cultural, económico y nutricional, así: 49 del Ministerio de Agricultura y Desarrollo Rural y EVA (Evalua- ciones Agropecuarias Municipales) [30], 14 del ICBF (Instituto Colombiano de

Bienestar Familiar) [31], cinco del Ministerio de Cultura [32] y dos del PNSAN (Plan Nacional de Seguridad Alimentaria y Nutricional) [33].

Creamos una metodología basada en datos y en cuatro pilares (es decir, origen geográfico, estatus de vulnerabilidad, beneficios económicos e importancia para la seguridad alimentaria) para ordenar y clasificar estos 345 RFAA desde los más prioritarios hasta los menos prioritarios para la inversión en investigación. Combinamos los datos de los cuatro pilares utilizando la lógica difusa. La disciplina de la lógica difusa es lo contrario del razonamiento probabilístico puro, donde todo en un universo es verdadero o falso. En cambio, la lógica difusa se aparta del principio de dicotomía y asume que todo es una cuestión de grado en la que ninguna es verdadera ni falsa [34]. Así, la función de membresía permite asignar diferentes membresías a cada elemento de información que contribuye en un universo de datos específico [35].

La metodología de lógica difusa empleada aquí tuvo varios pasos consecutivos (Fig. 2). El primer paso, comúnmente denominado preprocesamiento, consistió en la normalización y el escalado de los datos numéricos brutos. A continuación, asignamos una etiqueta y una función de pertenencia difusa a cada variable, lo que se denomina fuzzificación. El origen geográfico y el estado de vulnerabilidad correspondieron a una variable única para cada uno. En cambio, los beneficios económicos y la importancia de la seguridad alimentaria son el resultado de una combinación de cuatro variables para cada pilar, ya que en ellos influyen múltiples factores (Fig. 2A). Aunque puede haber dependencias entre variables dentro de un pilar dado, los cuatro pilares permanecen separados y distintos a la hora de determinar la prioridad de los RFAA.

No existe información para todas las variables para cada uno de los RFAA. Por lo tanto, en el grupo BGVCOL, imputamos la información si encontrábamos dentro del BGVCOL una especie del mismo género o de la misma categoría de la FAO y calculamos un nivel de incertidumbre para cada especie (Fig. 2B). Sin embargo, para el grupo NCB, no utilizamos una estrategia de imputación porque al ser externos al grupo BGVCOL, no teníamos en todos los casos una estrategia directa de imputación.

A continuación, tras obtener las variables directamente o imputadas, cada variable por cada pilar corresponde a un universo de datos X :[0,100] que contiene elementos de información que pueden ser discretos o continuos. Si la información era discreta, se utilizaban funciones de pertenencia de tipo singleton. En cambio, si la información era continua, utilizamos un trapecio (utilizado para las funciones de pertenencia de entrada) o una función gaussiana (función de pertenencia de salida) (Tabla 1). Definimos la función de pertenencia utilizando el paquete R Sets 1.0-18 [36] en R Studio ver. 3.6. (Tabla 1, Fig. 2C). Finalmente, utilizamos toda la información inferida para cada pilar para determinar el índice de prioridad y el nivel de incertidumbre para cada especie de los 345 RFAA. En las siguientes secciones se explica la fundamentación de cada paso con todo detalle.

2.1 Origen geográfico

Según su origen geográfico, clasificamos los 345 RFAA siguiendo la clasificación de las regiones de diversidad de los cultivos en el mundo [15] y la base de datos Powo [37] que reconoce 26 áreas a nivel mundial. Agrupamos las 26 regiones en tres clases (conjuntos difusos): locales, cercanas y lejanas. La etiqueta de local incluía los Andes y Sudamérica Tropical porque ambos contenían a Colombia. La etiqueta de cercano correspondió al Caribe, Centroamérica, México y Sudamérica templada porque son áreas vecinas a los dos locales. Por último, agrupamos las 22 regiones restantes dentro de la etiqueta de distante. Fueron Australia, Islas del Océano Índico, África Central, África Oriental, África Meridional, África Oriental, África Occidental, América del Norte, Asia, Asia Occidental, Asia Meridional, Asia Sudoriental, Asia Central, Asia Oriental, Asia Sudoriental, Europa, Europa Sudoriental, Mediterráneo Meridional, Europa Nororiental, Europa Sudoccidental, Europa Noroccidental y Mediterráneo Oriental. Por lo tanto, las funciones de pertenencia a este pilar son del tipo singleton con tres categorías de clasificación de prioridad, es decir, alta para lo local, media para lo cercano y baja para lo lejano (Tabla 1).

2.2 Estatus de vulnerabilidad

Clasificamos el estatus de vulnerabilidad de los 345 RFAA en cinco categorías: en peligro, vulnerable, casi amenazado, preocupación menor y no evaluado según el catálogo nacional colombiano [38]. Actualizamos la clasificación para tres especies locales en peligro *Elaeis oleifera*, *Bactris gasipaes* y *Passiflora jardinesis*, utilizando la resolución 1912 de 2017 del Ministerio de Ambiente y Desarrollo Sostenible de Colombia. Estas listas no contienen todas las especies consideradas en este estudio. En consecuencia, se consultaron las plataformas Jardín Botánico para la Conservación Internacional [39] y la Lista Roja [40]. Ambas bases de datos separaron las especies en seis categorías en lugar de cinco: en peligro crítico, en peligro, vulnerable, casi amenazada, preocupación menor y no evaluada. Por lo tanto, para analizar los 345 RFAA, fusionamos las categorías de vulnerabilidad nacional e internacional en tres: no evaluado, preocupación menor y amenazado.

La categoría no evaluada incluyó a todos los RFAA sin información nacional o internacional en las bases de datos consultadas. La categoría de preocupación menor combinó las categorías casi amenazada y de menor preocupación de las bases de datos nacionales e internacionales. Por último, la categoría amenazada incluyó las clasificaciones en peligro y vulnerable de la base de datos nacional y tres clases (es decir, en peligro crítico, en peligro y vulnerable) de las bases de datos internacionales. A partir de las categorías finales fusionadas de vulnerabilidad, la función de pertenencia para este pilar resultó del tipo singleton y con tres niveles de clasificación de las categorías amenazada con alta prioridad, preocupación menor con baja prioridad y no evaluada (Tabla 1).

2.3 Beneficios económicos

Identificamos las tendencias de la producción agrícola colombiana para cada uno de los 345 RFAA utilizando cuatro variables: Ingreso, índice de Lafay, cobertura municipal y rendimiento. Puede haber dependencias entre variables dentro del pilar de beneficios económicos, ya que múltiples factores pueden influir en cada variable. Utilizamos la información de cada variable por separado para organizar cada RFAA en categorías dentro de los grupos BGVCOL y NCB. Sin embargo, sólo clasificamos los RFAA tras integrar las cuatro variables en un único índice de beneficios económicos basado en la lógica difusa (Tabla 1 y Fig. 1). Para obtener la información sobre cada variable, utilizamos tres bases de datos (1) la Red de Información y Comunicación del Ministerio de Agricultura y Desarrollo Rural (MADR) para los datos sobre el área cosechada (ha), la producción (t) y el rendimiento (t ha^{-1}) de cada RFAA separados por municipios para diez años desde2007 hasta 2017 [30], (2) los precios al consumidor de cada RFAA del Sistema de Información de Precios del Sector Agropecuario (DANE) en 2019 [41], (3) la información de importación y exportación registrada en la base de datos LegisComex de 2019 [42].

2.3.1 Ingreso

Aumentar la productividad y los ingresos de los agricultores es un aspecto fundamental para reducir la pobreza rural y garantizar la seguridad alimentaria [43, 44]. En este caso, elegimos el ingreso por unidad de superficie como un indicador de la eficiencia económica de cada RFAA a nivel agregado en Colombia. Expresamos esta relación como $Inc_i = (Pi \times Pr_i)/A_i$. El Inc_i es el ingreso por hectárea de la especie *i* (unidades en USD ha^{-1}, donde los últimos datos disponibles son de 2017). El Pi indica el precio de venta de la especie *i* expresado en USD t^{-1}). El Pr es la producción anual de la especie *i* descrita en toneladas. Por último, el A_i es la superficie cosechada de la especie *i* expresada en hectáreas.

Etiquetamos los datos de ingresos como indeterminados, bajos, medios y altos. La etiqueta indeterminada se refiere a la información ausente o no disponible y tuvo un valor cero. Las otras tres etiquetas (baja, media y alta) corresponden a datos continuos disponibles con valores entre 1 y 50 (miles de USD ha^{-1}). La distribución de estos datos tuvo un rango amplio, por lo que los pre-procesamos utilizando un logaritmo $Log(Inc_i)$. Basándonos en $Log(Inc_i)$, calculamos el centroide de tres clusters (es decir, K=3) y asignamos los individuos con datos a uno de los tres grupos mediante k-means (paquete R Ckmeans.1d.dp [45]). Las etiquetas bajo, medio y alto tuvieron funciones de pertenencia trapezoidales para el RFAA (véase la Tabla 1 para los detalles sobre los parámetros de la función).

2.3.2 Índice de Lafay

Medimos el índice de Lafay (Li) para la contribución a la balanza comercial de la siguiente manera $Li = Pdi/(Pdi + Mi - Xi)$, donde Pdi es la producción anual de la especie *i* (t), Mi son las importaciones anuales de la especie *i* (t) y Xi son las exportaciones anuales de la especie *i* (t) [46]. El resultado de esta ecuación es un cociente entre la producción de un cultivo y su consumo aparente (es decir, la producción más la importación menos la exportación) en un año. Si este valor es superior a uno, el país es un exportador neto del cultivo, y cuanto mayor sea el nivel, más importantes son las exportaciones como destino de la producción nacional del cultivo.

Definimos cuatro categorías basadas en las interpretaciones del Índice de Lafay para dividir los RFAA analizados: indeterminado, bajo, medio y alto. La categoría indeterminada significa información no disponible en la base de datos, representada por una función única. Los datos tenían un rango de valores entre -0,07 y 5,28. A continuación, utilizamos funciones de pertenencia trapezoidales para las categorías baja (Índice de Lafay < 1), medio (Índice de Lafay = 1) y alto (Índice de Lafay > 1), para los RFAA analizados (véase la Tabla 1 para más detalles sobre los parámetros de la función).

2.3.3 Cobertura municipal

La cobertura municipal (%) es el porcentaje de municipios colombianos que cultivan una especie específica. Esta cobertura describe tanto la concentración como la adaptabilidad de cada especie a nivel regional. Así, los valores más cercanos al 100% representan cultivos muy extendidos y que son la base principal de los ingresos de los agricultores. La calculamos como $CM_i = (M_i/TM)100$, donde CM_i es la cobertura municipal para la especie *i* (en porcentaje). M_i es el número de municipios donde se cultiva la especie i. Además, TM es el número total de número de municipios colombianos. Utilizando esta ecuación, determinamos las clases para separar los RFAA analizados: indeterminado (función de pertenencia función de membresía), y cobertura estrecha, cobertura media y cobertura grande, con una función de pertenencia trapezoidal (véase la Tabla 1 para los detalles de los parámetros de la función).

2.3.4 Rendimiento

El rendimiento (t ha^{-1}) representa la productividad agrícola por superficie medida como $Ri = Pri/Ai$. Donde Ri es el rendimiento de la especie *i* (t ha^{-1}), Pri es la producción media anual en 10 años (2007-2017) de la especie *i* (t), y Ai es la superficie media cosechada de la especie *i* en 10 años (2007-2017) en ha en Colombia. Etiquetamos este conjunto de datos en cuatro categorías: Indeterminado, bajo, medio y alto. La categoría Indeterminado indica la ausencia de datos en la base de datos Agronet [30]. Para las otras tres categorías (es decir, baja, media y alta), agrupamos las especies en el rendimiento de los cultivos según las categorías de alimentos de la FAO [7] (Tabla 1).

Encontramos diferencias considerables en las escalas de rendimiento en los diez grupos de la FAO. Por lo tanto, tuvimos que normalizar todos los datos antes de aplicar la lógica difusa. Utilizamos k-means (k=3) para las tres categorías alto, medio y bajo del paquete CKmeans.1d.dp [45] para obtener los centroides por separado dentro de cada uno de los diez grupos de la FAO. La función de pertenencia fue un singleton para la categoría indeterminada y un trapezoide para las otras tres categorías (es decir, rendimiento bajo, rendimiento medio y rendimiento alto) (véase la Tabla 1 para más detalles sobre los parámetros de la función).

2.3.5 Reglas de lógica difusa para los beneficios económicos

El índice de beneficios económicos integra cuatro variables: el índice de Lafay, el rendimiento (t ha-*1*), el ingreso (USD ha^{-1}) y la cobertura municipal (%). Cada una de estas cuatro variables tuvo cuatro opciones para cada etiqueta y un valor único asignado a través de su función de pertenencia con el fin de clasificar los RFAA bajo análisis (Tabla 1). Por lo tanto, definimos reglas para las 256 combinaciones diferentes (cuatro variables con cuatro opciones cada una) para describir los beneficios económicos de cada RFAA dentro de este estudio.

Calculamos una categoría de beneficios económicos como resultado de las 256 reglas utilizando la ecuación $ID = \sum_{z=1}^{Y}(S_z w_z) \times 100; Y = 4$. El ID es el índice de decisión, y S_z representa la puntuación de cada variable (es decir, 0 para no tener información, 1 para baja, 2 para media y 3 para alta). Además, el w_z es el peso de cada variable, que es 0,25 cuando $\sum w_z = 4$. Con esta ecuación, utilizamos una función de pertenencia trapezoidal para representar cuatro clases diferentes. Si $ID < 0{,}24$, la clase de salida fue "sin decisión". Si $0{,}25 \; < ID < 0{,}45$, la clase de salida fue "bajos beneficios económicos". Si $0{,}46 < ID < 0{,}64$, la clase de salida fue "beneficios económicos medios". Por último, si $0{,}65 < ID < 1$, la clase de salida fue de “beneficios económicos altos”. Estas tres categorías también representan los tres niveles de prioridad para los RFAA analizados para este pilar (Tabla 1).

2.4 Importancia para la seguridad alimentaria

El segundo Objetivo de Desarrollo Sostenible (ODS) desafía al mundo a lograr la seguridad alimentaria y mejorar la nutrición para 2030 [18]. En consecuencia, adoptamos un enfoque

multidimensional para evaluar cuatro variables de seguridad alimentaria para cada RFAA en este estudio. Se trata de la asequibilidad basada en los nutrientes, la contribución nutricional, el hecho de formar parte de la lista de prioridades del gobierno y la lista de consumo tradicional por regiones. Puede haber dependencias entre variables dentro del pilar de beneficios económicos, ya que múltiples factores pueden influir en cada variable. Utilizamos la información de cada variante por separado para organizar cada RFAA en categorías dentro de los grupos BGVCOL y NCB. Sin embargo, sólo clasificamos los RFAA tras integrar las cuatro variables en un índice de importancia para la seguridad alimentaria basado en la lógica difusa.

2.4.1 Contribución nutricional

Colombia ha realizado importantes progresos económicos y sociales en las últimas décadas. Aunque su clasificación actual es la de un país de renta media-alta, todavía existen retos considerables para lograr una convergencia hacia niveles de vida más altos. En este sentido, las deficiencias de micronutrientes siguen prevaleciendo entre los niños menores de cinco años y contribuyen al deterioro del desarrollo infantil y al aumento de la carga de enfermedad nacional [47]. Se evaluaron los 275 RFAA en base a su contribución a los requerimientos de ingesta diaria de nutrientes para garantizar la suficiencia alimentaria de tres micronutrientes (Calcio, Hierro y Zinc) en la población infantil del país.
Desarrollamos dos ecuaciones basadas en la composición nutricional de cada especie [31], el objetivo dietético diario de tres micronutrientes deficientes en la población colombiana (es decir, Calcio, Hierro y Zinc) [48], y el consumo de cada especie por día [48]. En primer lugar, se calculó el aporte medio diario AD_{ij}de la especie i en función de sus micronutrientes *j*. Se estimó $AD_{ij} = \left(CD_i \times M_j\right)/100$ donde CD_i representaba el consumo lácteo de la especie *i* en gramos (g/día) y M_j era la composición media del micronutriente *j* en 100 gramos de alimento comestible (mg de *j* /100 g de *i*). A continuación, estimamos la contribución de la especie *i* a las necesidades diarias de micronutrientes *j* en porcentaje (%), así $C_{ij} = AD_{ij}/MN_j \times 100$. En la ecuación, C_{ij} era la contribución de la especie i a las necesidades diarias de micronutrientes *j* (en porcentaje). El AD_{ij} era la contribución nutricional media del micronutriente *j* durante un día (mg /día), y el MN_j era el objetivo de micronutrientes *j* en un día (mg/día).

Definimos un sistema difuso para la contribución global, en el que las entradas son una contribución media diaria de cada micronutriente (es decir, Calcio, Hierro, Zinc y contribución energética). Se establecieron cuatro categorías: indeterminada (ausencia de información), baja, media y alta contribución media diaria de cada micronutriente. El tipo indeterminado para todos los micronutrientes tenía una función de pertenencia simple, mientras que los otros tres tenían funciones de pertenencia trapezoidal. A continuación, normalizamos los datos disponibles para cada micronutriente en valores de 1 a 100. Por último, definimos los parámetros de la función de pertenencia basándonos en la distribución de cuartiles de los datos normalizados (Tabla 1).

Al igual que las reglas difusas definidas para los beneficios económicos, la categoría de salida de la contribución nutricional fue la siguiente. Si $ID < 0{,}24$, entonces la especie no tiene suficiente

información para decidir su contribución nutricional. Si $0{,}25 < ID < 0{,}45$, la especie tiene una contribución nutricional baja. Si $0{,}46 < ID < 0{,}64$ la especie tenía una contribución nutricional media. Por último, si $0{,}65 < ID < 1$, la especie tuvo una contribución nutricional alta (Tabla 1).

2.4.2 Asequibilidad basada en sus nutrientes

Definimos la asequibilidad de los 345 RFAA en términos monetarios para suplir la carencia de micronutrientes con recursos locales. Calculamos la relación nutriente-precio como el precio al consumidor en USD por cada 100 gramos de una porción comestible en cuatro variables: unidades de Calcio (mg), unidades de Hierro (mg), unidades de Zinc (mg), y unidades de energía (kcal). A continuación, estimamos la competitividad de la especie desde el punto de vista nutricional y de mercado utilizando estos valores.

Definimos un sistema difuso para obtener la asequibilidad por especie; en este caso, los inputs son el precio medio por micronutriente (es decir, el aporte energético, el Calcio, el Hierro y el Zinc). Los datos disponibles para cada micronutriente se normalizaron por separado en el rango de 1 a 100 y, a continuación, basándonos en la distribución de cuartiles de los datos normalizados, definimos los parámetros de la función de pertenencia. Se clasificaron los datos como indeterminados (ausencia de información), bajos, medios y altos. La categoría desconocida tenía una función de pertenencia única, mientras que utilizamos funciones de pertenencia trapezoidales para los otros tres tipos de clasificación (Tabla 1).

Por último, calculamos una categoría de contribución nutricional combinando la asequibilidad de los cuatro nutrientes. Utilizamos el mismo enfoque para definir las reglas difusas en los beneficios económicos con los mismos umbrales de identificación para estimar la clase de salida para la combinación de variables. Así, las clases de salida tenían cuatro categorías objetivo: indeterminada, baja, media y alta. Las especies para el aumento de la asequibilidad de los nutrientes, y el mayor porcentaje del objetivo nutricional de cada micronutriente, corresponden a las especies con mayor prioridad (Tabla 1).

2.4.3 Lista de prioridades del Gobierno

En 2013, el Gobierno colombiano definió una lista de recursos fitogenéticos para mejorar su consumo estable en la dieta de la población colombiana y garantizar políticas para mejorar su producción y suministro [33] (Fig. S1). Utilizamos esta información para construir una función de membresía singleton con dos etiquetas, "incluido" y "no incluido" (Tabla 1).

2.4.4 Consumo tradicional por regiones

Esta variable relacionó las tradiciones agrícolas y alimentarias que reflejan el carácter multicultural, multiétnico y biodiverso de Colombia en 11 regiones geográficas [32] (Fig. S2). A partir de esta información se obtuvo una variable numérica que muestra el número de regiones donde cada especie es esencial para la tradición alimentaria. Asignamos

una etiqueta con tres categorías basadas en el número de áreas en las que aparece cada especie. Uso restringido (es decir, entre una y tres regiones), uso medio (es decir, entre cuatro y seis áreas) y uso amplio (es decir, entre siete y 11 regiones).

La función de pertenencia fue de un único valor para la categoría indeterminada y correspondió a los datos faltantes. Además, la función de pertenencia para las otras tres categorías (es decir, uso restringido, medio y amplio) fue un trapezoide. Asimismo, aplicamos la normalización de rangos sobre los datos disponibles de uno a ocho en uno a 100 (Tabla 1).

2.4.5 Reglas de lógica difusa para la seguridad alimentaria

Construimos el indicador de seguridad alimentaria para cada especie vegetal integrando cuatro variables. En primer lugar, la lista de prioridades del gobierno (A_p) con dos etiquetas: incluido o no incluido [33]. En segundo lugar, el consumo tradicional (T_c) contiene el uso restringido, medio y amplio [32]. En tercer lugar, el aporte nutricional (C_N) con cuatro etiquetas: bajo, medio, alto e indeterminado [31]. Por último, la asequibilidad de los nutrientes (P_N) con cuatro etiquetas: indeterminada, baja, media y alta. Así, el número de combinaciones por cada variable y etiqueta fue $A_p(2) \times T_c(3) \times C_N(4) \times P_N(4) = 96$.
A continuación, calculamos un valor de índice mediante la ecuación $ID = \sum_{z=1}^{Y}(S_z w_z) \times 100; Y = 4$ (igual que las reglas difusas de los beneficios económicos). El ID es el índice de decisión. La S_z representa la puntuación de cada variable, y tiene diferentes valores en función de las etiquetas. Para las etiquetas no incluidas uso estrecho y no determinado $S_z = 0$. Para las etiquetas uso medio y bajo $S_z = 1$. Además, para las etiquetas incluidas, de uso extensivo y alto $S_z = 3$. Por último, el w_z es el peso de cada variable, que es de 0,25, desde 0 a 1 donde $\sum w_z = 1$. Según esta ecuación, si $ID < 0{,}24$, la especie no tiene información para decidir su importancia en la seguridad alimentaria. Si $0{,}25 < ID < 0{,}44$, la especie tuvo una importancia baja para la seguridad alimentaria. Si $0{,}45 < ID < 0{,}64$, la especie tuvo una importancia media para la seguridad alimentaria. Si $0{,}65 \quad < ID < 1$, la especie tuvo una importancia de seguridad alimentaria alta (Tabla 1).

2.5 Imputación de datos y niveles de incertidumbre para cada RFAA analizado

Muchos de los RFAA conservados en el BGVCOL tienen una limitación de datos sobre el beneficio económico (62%) y la importancia para la seguridad alimentaria (73%). Una solución fiable para la ausencia de información fue imputar los valores aplicada exclusivamente para el grupo BGVCOL. Dentro del grupo BGVCOL, disponíamos de información para completar los datos que faltaban, ya sea del cultivo representativo más cercano o de la especie más cercana del mismo género (es decir, imputación del pool genético - GP) o de la misma categoría de alimentos definida por la FAO (es decir, el grupo de cultivos provisional - PCG). Por el contrario, no fue así en el caso del grupo de NCB, ya que en la mayoría de los casos representan cultivos únicos. Por lo tanto, la búsqueda de posibles GP o PCG implicaría la selección de especies fuera de los 70 PGRFA de las bases de datos gubernamentales. Así, en ausencia de datos para los RFAA del grupo NCB, los clasificamos directamente como indeterminados.

Aplicamos esta estrategia de imputación dentro del grupo BGVCOL a tres pilares: origen geográfico, beneficio económico e importancia para la seguridad alimentaria. Omitimos el pilar de la vulnerabilidad porque estas variables son únicas para cada especie. Por lo tanto, los niveles de vulnerabilidad entre los cultivos y los parientes silvestres podrían ser opuestos, aunque pertenezcan al mismo género o a la misma categoría alimentaria de la FAO.
El procedimiento siguió esta secuencia. En caso de falta de información para los RFAA dentro del grupo BGVCOL, se buscó una especie dentro del grupo GP como primera opción. Si no había especies disponibles dentro del grupo GP, imputamos los datos utilizando el grupo PCG. Para este caso, elegimos una especie que fuera el peor escenario en términos de datos para evitar el sesgo debido a la imputación (Tabla 2). Por último, si no había grupos GP ni PCG que imputar, el RFAA quedó como indeterminado. Paralelamente al proceso de imputación, también asignamos una etiqueta de incertidumbre. Si la información existe para el RFAA o era indeterminada, la etiqueta asignada fue "fiable". En caso contrario, se dispuso de dos etiquetas posibles cuando faltaba la información y se imputó los datos. La etiqueta fue "incertidumbre GP" si se imputó a partir del pool genético (GP) o "incertidumbre PCG" si se imputó con el grupo de cultivos provisional definido por la FAO (Tabla 1, Fig. 2B).

2.6 Reglas de lógica difusa utilizadas para cada grupo de información

Una vez calculadas las variables de cada uno de los cuatro pilares y el nivel de incertidumbre para cada uno, definimos las reglas para la clasificación final de interés para la lista de 345 RFAA (es decir, 275 conservados en el BGVCOL y 70 del grupo NCB). Utilizamos una función de pertenencia gaussiana para las clases de salida y utilizamos un centroide para mapear desde la función de pertenencia a un valor que es el interés del BGVCOL.

Si 4/4 o 3/4 de los pilares tenían una etiqueta "alta", el interés del BGVCOL se clasificaba como de alta prioridad. Si el origen geográfico tenía una etiqueta “media” o "alta", y 2/3 de los otros tres pilares tenían etiquetas superiores o iguales a la categoría media, clasificamos el RFAA como de prioridad media. En caso contrario, clasificamos el RFAA como de baja prioridad (Tabla 1).

3 Resultados

El análisis de todos los pilares de información generó un índice que clasificó los 345 RFAA con valores de 0 a 100. De los 275 RFAA del grupo BGVCOL, 24 resultaron en prioridad alta (8,72%), 72 en prioridad media (26,18%) y 179 en prioridad baja (65,09%) (Fig. 3). Los 24 RFAA en la clase de prioridad alta representan siete taxones: papa (15 especies), tomate (3 especies), tomate de árbol (2 especies), piña, cacao, papaya y yacón (Fig. 4). De los 24 RFAA, 20 son del género *Solanum*, que es también el género más representado en el BGVCOL (Fig 1). Además, estos cultivos pertenecen a cuatro categorías alimentarias de la FAO: raíces y tubérculos, frutas

y frutos secos, hortalizas y melones, y otros cultivos. La clasificación final de los 70 NCB no conservados en el BGV mostró solo una especie en prioridad alta (1,42%), el café (*Coffea arabica*, del grupo de cultivos de bebidas y especias de la FAO. Además, 11 (15,72%) resultaron en el nivel de interés medio y 58 (82,86%) en el nivel más bajo. A continuación, presentamos los resultados obtenidos por cada pilar después de aplicar la lógica difusa, organizando las tendencias por los grupos de alimentos de la FAO (es decir, la mediana, la media y el error estándar), y destacando los RFAA que identificamos como de alta prioridad para la inversión en investigación y conservación.

3.1 Origen geográfico

Este pilar tuvo la categoría indeterminada más pequeña, con sólo tres especies sin información sobre su origen geográfico (*Solanum chrenhkigii*, *Solanum lobbianum* y *Solanum faoensis*). Estos corresponden al 0,86% de las 345 especies de RFAA de los dos grupos. Las imputamos en la categoría de origen cercano, considerando la amplia distribución del género *Solanum* en América Central y del Sur. Tras la imputación de estas tres especies, ninguna de las RFAA analizadas se encontraba en la categoría de origen indeterminado. Utilizando esta información de origen geográfico, encontramos que el BGVCOL conserva RFAA de 21 regiones de las 26 a nivel mundial, mientras que el grupo NCB representó 16 regiones (Fig. S3).
El 44% (n=121) de los RFAA conservados en el BGVCOL son locales y, por lo tanto, tienen una alta prioridad porque son originarios de América del Sur tropical (n=88) y de los Andes (n=33), dos regiones en las que Colombia está localizada. Además, el 18,55% (n=51) de los RFAA son originarios de Centroamérica y México, el Caribe y los tres imputados, lo que representa un origen cercano y una prioridad media. El otro 37,45% (n=103) se originó en regiones distantes y son de baja importancia (Fig. 3 y S3A). De la lista de alta prioridad (n=121), 70 son frutas y frutos secos, 20 son raíces y tubérculos, nueve son hortalizas y melones, cinco son bebidas y especias, cuatro son leguminosas, tres son oleaginosas, una es un cereal y nueve corresponden a otros cultivos (Fig. S12A).

En el caso de los 70 RFAA del grupo BCN, el 61% (n=43) proceden de regiones lejanas y, por tanto, tienen una prioridad baja. Por otra parte, el 30% (n=21) son locales (es decir, 17 formados en Sudamérica Tropical y cuatro de los Andes) con prioridad alta. Por último, el 8,5% (n=6) tienen el origen más cercano (América Central y México) con una prioridad intermedia (Fig. S3B). El NCB de alta prioridad (n=21) representa siete categorías de alimentos de la FAO, a saber: cuatro cultivos de bebidas y especias, cuatro de frutas y frutos secos, uno de leguminosas, dos de semillas oleaginosas, cinco de raíces y tubérculos, uno de hortalizas y melones, y cuatro clasificados como otros cultivos.

3.2 Estatus de vulnerabilidad

De los 345 RFAA del estudio, encontramos que el 48% (n=132) del grupo BGVCOL y el 38,6% (n=27) del grupo NCB no tienen datos asociados a su estado de vulnerabilidad en las bases de datos analizadas. Las categorías de alimentos de la FAO con más datos de ausencia sobre el

estado de vulnerabilidad de los RFAA analizados fueron los cultivos de azúcar (n=1), las hortalizas y los melones (n=20) y los tubérculos y raíces (n=33) (Fig. S4A). En cuanto a la región geográfica de origen, el mayor número de datos faltantes correspondió a Sudamérica tropical (n=34), seguido de Centroamérica y México (n=22) y los Andes (n=25) (Fig. S4B). Los datos faltantes correspondieron a la categoría indeterminada para este pilar, y no aplicamos una estrategia de imputación. Después del proceso de lógica difusa, encontramos que el 6,5% (n=18) de los RFAA conservados en el BGVCOL están en la categoría de amenaza prioritaria. Además, el 45,4% (n=125) tenía una preocupación menor y una prioridad baja (Fig. 3). El BGVCOL amenazado representa cinco categorías de alimentos de la FAO: 13 frutas y frutos secos, una leguminosa, una oleaginosa, una hortaliza y melones, y dos clasificadas como otros cultivos (Fig. S12B).

En el grupo de NCB, tres RFAA (4,3%) están en la categoría de amenazados con alta prioridad de conservación, y 40 (57,1%) tienen una preocupación menor. Los RFAA amenazados representan dos categorías de alimentos de la FAO: dos bebidas y especias (café - *Coffea arabica*, y cardamomo - *Elettaria cardamomum*) y una semilla oleaginosa (almendra - *Prunus dulcis*).

3.3 Beneficios económicos

El pilar de los beneficios económicos integró cuatro variables: Índice de Lafay, rendimiento, cobertura del municipio e ingresos. Para este pilar, se aplicó un proceso de imputación dentro del grupo BGVCOL. Después de este proceso, 54 (19,6%) de los RFAA tuvieron prioridad alta, 80 (29%) con prioridad medio, 56 (20,3%) con prioridad baja y 85 (30,9%) indeterminados de los 275 analizados (Fig. 3). Los 54 grupos de especies representan 15 grupos taxonómicos con alta prioridad, así uchuva (n=13), arroz (n=1), piña (n=1), batata (n=1), caña de azúcar (n=1), tomate (n=3), papa (n=23), yacón (n=1), cebolla de rama (n=1), papaya (n=1), palma (n=1), algodón (n=3), frijol (n=1), cacao (n=2) y cítricos (n=1). De ellos, la batata, el tomate y la caña de azúcar presentaron los ingresos más altos. El cacao y los cítricos presentaron el mayor índice de Lafay. Además, la caña de azúcar y el cacao tuvieron la mayor cobertura de municipios. Finalmente, las especies con los grupos de mayor rendimiento fueron el arroz, la piña, la caña de azúcar, el tomate, la papa, la palma, el algodón y el frijol (Fig. S14A). En comparación, para el grupo NCB, no aplicamos una metodología de imputación, y los 70 RFAA se distribuyeron así 11 (15,7%) con prioridad alta, 13 (18,6%) con prioridad media, 28 (40%) con baja, y 18 (25,7%) indeterminados (Fig. 3). Los 11 con prioridad alta fueron: *Coffea arabica* (café), *Zingiber officinale* (jengibre), *Raphanus sativus* (rábano), *Curcuma longa* (azafrán indio), *Spinacia oleracea* (espinacas), *Lactuca sativa* (lechuga), *Opuntia ficus*-indica (nopal), *Beta vulgaris* (remolacha azucarera), *Brassica oleracea* (col), *Fragaria vesca* (fresa silvestre) y *Manihot esculenta* (yuca). A continuación, describimos los resultados medios de cada variable, recopilando los RFAA por las categorías de alimentos de la FAO para mostrar las principales tendencias.

3.3.1 Proceso de imputación dentro del grupo BGVCOL

Dentro del grupo BGVCOL, 171 (62%) RFAA tuvieron información faltante sobre las cuatro variables consideradas en el pilar de beneficios económicos. Para este grupo, 86 RFAA tuvieron un grupo de genes (GP) o un grupo de cultivos provisional (PCG) para imputar los valores (Tabla 2). Así, tras la imputación, 85 (31%) resultaron indeterminados, 54 (20%) altos, 80 (29%) medios y 56 (20%) en la categoría baja de la clasificación (Tabla 3).

3.3.2 Ingresos

Para el grupo BGVCOL, encontramos información sobre esta variable para 55 (20%) de los RFAA. La visualización de los ingresos (miles USD/ha) para cada RFAA organizados por categorías de alimentos de la FAO mostró que los cultivos de azúcar presentaron los mayores ingresos, seguidos por las hortalizas y melones, raíces y tubérculos, semillas oleaginosas, bebidas y especias, frutas y frutos secos, otros cultivos, cereales, leguminosas (Tabla 4 y Fig. S6A). En el caso del BCN, 38 (54%) contaban con información para esta variable. Separando los RFAA por categorías de alimentos de la FAO, las hortalizas y los melones tuvieron la media de ingresos más alta, seguidos de las bebidas y especias, las frutas y frutos secos y las leguminosas. Por el contrario, las raíces y tubérculos presentaron unos ingresos más bajos (Tabla 4, Fig. S7A).

3.3.3 Índice de Lafay

Para el grupo BGVCOL, sólo encontramos la información para crear este índice para 88 (32%) especies. Por lo tanto, imputamos utilizando otros RFAA dentro del BGVCOL correspondientes al grupo GP o PCG (Tabla 2). Tras el proceso de imputación, la media del índice de Lafay por categorías de alimentos de la FAO mostró que los cereales, las leguminosas y hortalizas y los melones tienen una producción insuficiente para las necesidades del país (es decir, media <1). Por lo tanto, representan el grupo de baja prioridad porque su importación es obligatoria. El caso opuesto es el de las especies de la categoría de otros cultivos, las frutas y frutos secos, que indican que Colombia los exporta principalmente (es decir, media >1) y tienen prioridad alta. Finalmente, los cultivos de azúcar, raíces y tubérculos, oleaginosas, y bebidas y especias mostraron igual producción y consumo interno (i.e., media = 1) con prioridad media (Tabla 4, Fig. S6B).

En el caso del BCN, 61 (87%) de los RFAA contaban con información para generar este índice. Separándolos por categorías de alimentos FAO, encontramos que Leguminosas, frutas y frutos secos, y hortalizas y melones tienen valores medios < 1, indicando que el país está importando estos RFAA debido a la baja producción nacional. Por lo tanto, tienen una prioridad baja para este índice. Las otras categorías, como cultivos de azúcar, raíces y tubérculos, semillas oleaginosas y otros cultivos, indican una producción y un consumo similares en el país, con una prioridad media (Tabla 4, Fig. S7B).

3.3.4 Cobertura municipal

Para el grupo BGVCOL, encontramos información sobre esta variable para 85 (31%) de las especies. La visualización de la cobertura municipal (%) para cada RFAA organizada por categorías de alimentos de la FAO mostró que los cultivos de azúcar tenían la mayor cobertura, seguidos por los cereales. Las demás categorías tenían una cobertura municipal alrededor de 10%. Entre ellas se incluyeron semillas oleaginosas, raíces y tubérculos, hortalizas y melones, otros cultivos, frutas y frutos secos, leguminosas, y bebidas y especias (Tabla 4, Fig. S6C).

En el caso del BCN, 36 (51%) de los RFAA tuvieron información para esta variable. Separándolos por categorías de alimentos de la FAO, encontramos que otros cultivos, frutas, frutos secos, hortalizas y melones tienen una cobertura baja (~ 10%). Además, los cultivos de azúcar (n=1) y leguminosas (n=3) no tienen información de cobertura municipal en el país, con una media de 0,1% para ambas categorías (Tabla 4, Fig. S7C).

3.3.5 Rendimiento

Para el grupo BGVCOL, encontramos información sobre esta variable para 115 (42%) de las especies. El rendimiento (t ha^{-1}) medio por categorías de alimentos de la FAO mostró que los cultivos azucareros tenían el mayor rendimiento en comparación con las demás categorías. Las hortalizas y los melones, las semillas oleaginosas y las raíces y tubérculos tuvieron un rendimiento superior a > 10 t $ha\text{-}^{1}$. En cambio, las frutas y frutos secos, los cereales, las leguminosas, las bebidas y especias, y otros cultivos tuvieron un rendimiento < 10 t ha^{-1} (Tabla 4 y Fig. S6D).

En el caso del BCN, 66 (94\%) de los RFAA contaban con información para esta variable. Separándolos por categorías de alimentos de la FAO, encontramos que hortalizas y melones, frutas y frutos secos, raíces y tubérculos tenían la media de rendimiento más alta (es decir, > 10 t ha^{-1}). En cambio, las semillas oleaginosas, las bebidas y especias, los cultivos de azúcar, las leguminosas y otros cultivos tuvieron la media más baja (es decir, < 10 t ha^{-1}) (Tabla 4 y Fig. S7D).

3.4 Importancia para la seguridad alimentaria

El pilar de la seguridad alimentaria está compuesto por cuatro variables: lista de prioridades del gobierno, consumo tradicional, contribución nutricional y asequibilidad basada en sus nutrientes. Para los 275 RFAA de la BGVCOL, obtuvimos tras la imputación 36 (13%) en importancia alta, 76 (27,6%) en media, 60 (21,81%) en baja, y 103 (37,45%) indeterminada (Fig. 3). Los 36 grupos de especies con alta importancia incluyen arroz, cacao, cucurbitáceas, mango, piña, papaya, batata, frijol, guayaba y papa. A excepción de la piña y el cacao, todas las especies muestran una menor asequibilidad en función de sus nutrientes (es decir, una mayor importancia). La contribución nutricional también es muy alta para los 36 taxones, el arroz y la papa con valores más bajos que los demás. Además, todas las especies son importantes para el Gobierno en la priorización de la lista. Por último, el arroz es esencial para el consumo

tradicional, con un valor medio para la piña, frijol y papa. El resto de cacao, cucurbitáceas, mango, papaya, batata y guayaba tuvieron menos importancia para este factor (Fig. S13B).

En el caso de los 70 RFAA del grupo NCB, ninguno resultó en la categoría de importancia alta; 19 (27,1%) en la media, 12 (17,1%) en la baja y 39 (55,7%) fueron indeterminados, sin ningún proceso de imputación (Fig. 3). A continuación, describimos los resultados medios de cada variable, recopilando los RFAA por las categorías alimentarias de la FAO para mostrar las principales tendencias.

3.4.1 Proceso de imputación dentro del grupo BGVCOL

Para el grupo BGVCOL, 201 (73%) RFAA no tuvieron ninguna información sobre la importancia del pilar de seguridad alimentaria (es decir, indeterminada). 98 RFAA tenían un grupo de genes o un grupo de cultivos provisional para imputar los valores (Tabla 2). Por lo tanto, 103 (37%) fueron indeterminados. Además, tras la imputación, 36 (13%) se encontraron en el rango alto, 76 (28%) en el medio y 60 (22%) en el rango de priorización baja (Tabla 3). En el caso del NCB, no se imputó ninguno de los 70 RFAA.

3.4.2 Asequibilidad basada en sus nutrientes

Para analizar esta variable, utilizamos información sobre tres micronutrientes, Calcio (Ca), Hierro (Fe), Zinc (Zn), y unidades de energía. En el grupo BGVCOL, encontramos que la información sobre esta variable estaba desequilibrada para cada nutriente considerado. Para el Calcio, encontramos datos disponibles en 64 (24%), Hierro 64 (23%), Zinc 29 (11%), y unidades de energía 67 (24%) de los 275 RFAA analizados en este grupo (Fig. S8).

La visualización de la asequibilidad de los nutrientes organizada por categorías de alimentos de la FAO mostró que los USD más baratos por 100 g de porción comestible para el Ca fueron los cultivos de azúcar, las leguminosas, las hortalizas y los melones, seguidos de los cereales, las frutas y los frutos secos. El Ca más caro por 100 g de porción comestible correspondió a bebidas, especias, otros cultivos, raíces y tubérculos (Tabla 5 y Fig. S8A). En el caso de Fe, la categoría más barata fue la de los cultivos de azúcar, seguida de los cultivos de leguminosas, frutas y frutos secos, cereales, hortalizas y melones, y otros cultivos. Las categorías más caras fueron bebidas, especias, raíces y tubérculos (Fig. S8B). Además, el USD más barato para 100 g de porción comestible con Zn fue el de los cultivos de azúcar, seguido de los cereales, las hortalizas y los melones, las frutas y los frutos secos, y las leguminosas. Las categorías más caras (es decir, > 0,4 USD) fueron raíces y tubérculos y otros cultivos (Tabla 5, Fig. S8C). Por último, en cuanto a la energía, la categoría más barata fue la de los cultivos azucareros. A continuación, se sitúan los cereales, las frutas y frutos secos, las raíces y tubérculos, y las hortalizas y melones. Las categorías más caras (es decir, > $3,6 \times 10^{-3}$ USD) fueron otros cultivos y bebidas, y especias (Tabla 5, Fig. S8D). La asequibilidad de los nutrientes indicó que 36 (13,1%) son de alta prioridad con menores gastos, 6 (2,2%) son medios, 20 (7,6%) en la prioridad más baja debido a su mayor costo, y 212 fueron indeterminados para esta variable.

En comparación, en el caso del grupo NCB, encontramos que la información sobre esta variable estaba desequilibrada para cada nutriente considerado en el estudio. Para el Calcio, encontramos datos disponibles en 23 (33%), Hierro 21 (30%), Zinc 8 (11%), y unidades de energía 19 (27%) de los 70 RFAA analizados (Fig. S9). Visualizando los micronutrientes que componían esta variable organizada por categorías de alimentos de la FAO, encontramos que la más barata en USD por 100 porciones comestibles para el Ca era raíces y tubérculos y leguminosas, seguida de hortalizas y melones, y frutas y frutos secos. La categoría más cara fue la de bebidas y especias (Tabla 5, Fig. S9A). En el caso del Fe, la categoría más barata fue la de leguminosas, seguida de raíces y tubérculos, verduras y melones. El coste más elevado correspondió a frutas, frutos secos, bebidas y especias (Tabla 5, Fig. S9B). Además, la categoría más barata para el Zn fue la de raíces y tubérculos, seguida de verduras y melones. El costo más elevado correspondió a las frutas y frutos secos (Tabla 5, Fig. S9C). Por último, en el caso de la energía, el valor más barato correspondió a raíces y tubérculos, seguido de frutas y frutos secos, bebidas y especias, y leguminosas. La categoría más cara fue la de hortalizas y melones (Tabla 5, Fig. S9D). El análisis de la asequibilidad de los nutrientes por categorías indicó que 12 (17,4%) resultaron de mayor prioridad por su bajo costo, 5 (7,1%) de prioridad media y 4 (5,7%) de menor prioridad por su mayor precio, y 49 (70%) fueron indeterminados para esta variable.

3.4.3 Contribución nutricional

Se utilizó información sobre tres macronutrientes, Calcio (Ca), Hierro (Fe), Zinc (Zn), y unidades de energía. La disponibilidad de esta información fue desequilibrada para cada nutriente considerado. En el grupo BGVCOL, los RFAA con datos disponibles fueron para Calcio en 200 (73%), Hierro 184 (67%), Zinc 102 (37%), y unidades de energía 198 (72%) de los 275 RFAA analizados en este grupo (Tabla 6, Fig. S10). La visualización de esta variable organizada por categorías de alimentos de la FAO separando cada micronutriente mostró que el mayor porcentaje del objetivo nutricional diario de Ca correspondía a bebidas y especias, cultivos azucareros y hortalizas y melones. Las demás categorías tuvieron un porcentaje de Ca <10%. Son, en orden descendente, leguminosas, cereales, frutas y frutos secos, otros cultivos, raíces y tubérculos, y semillas oleaginosas (Tabla 6, Fig. S10A). En el caso del Fe, las categorías con un mayor porcentaje del objetivo nutricional diario (es decir, >10%) fueron bebidas y especias, cultivos de azúcar, cereales y leguminosas. Las otras categorías con <10\% incluyeron frutas y frutos secos, otros cultivos, hortalizas y melones, semillas oleaginosas y raíces y tubérculos (Tabla 6, Fig. S10B). Además, en el caso del Zn, el mayor porcentaje del objetivo nutricional diario correspondió a cereales, frutas y frutos secos. Las otras categorías con <10% fueron semillas oleaginosas, raíces y tubérculos, leguminosas, otros cultivos y hortalizas y melones (Tabla 6, Fig. S10C). Por último, en cuanto a la energía, los cereales, los cultivos azucareros, otros cultivos y las leguminosas fueron los que alcanzaron el porcentaje más alto del objetivo nutricional diario (es decir, >10%). Las otras categorías con <10% incluyeron raíces y tubérculos, frutas y frutos secos, bebidas y especias, semillas oleaginosas y hortalizas y melones (Tabla 6 y Fig. S10D). El análisis de la contribución nutricional por categorías de prioridad indicó que 25 (9,1%) resultaron en la prioridad más alta con mayor contribución, 17 (6,2%) en la prioridad

media con contribución media, y 18 (6,5%) en la prioridad más baja debido a su menor contribución, y 214 fueron indeterminadas para esta variable.

En comparación, en el grupo NCB, los datos disponibles para Calcio fueron 26 (37%), Hierro 23 (33%), Zinc 8 (11%), y unidades de energía 21 (30%) de los 70 RFAA analizados en este grupo (Fig. S11). La visualización de esta variable organizada por categorías de alimentos de la FAO separando cada micronutriente mostró que el mayor porcentaje del objetivo nutricional diario de Ca correspondía a bebidas y especias, y leguminosas. Las otras categorías con <10% fueron verduras y melones, frutas y frutos secos, y raíces y tubérculos (Tabla 6, Fig. S11A). En el caso del Fe, el mayor porcentaje del objetivo nutricional diario correspondió a bebidas y especias, verduras y melones, leguminosas, y raíces y tubérculos. La categoría con $<$10\% fue frutas y frutos secos (Tabla 6, Fig. S11B). Además, los porcentajes más altos del objetivo nutricional diario de Zn fueron las bebidas y especias, las verduras y melones, y las raíces y tubérculos. La categoría con un 10% fue la de frutas y frutos secos (Tabla 6, Fig. S11C). Por último, en el caso de la energía, las categorías con un mayor porcentaje del objetivo nutricional diario fueron las bebidas y especias y las raíces y tubérculos. Las categorías con <10% fueron frutas, frutos secos, leguminosas, verduras y melones (Tabla 6, Fig. S11D). El análisis de la contribución nutricional por categorías prioritarias indicó que 10 (14,3%) resultaron en la prioridad más alta con mayor contribución, 10 (14,3%) en la prioridad media con contribución media, y 7 (10%) en la prioridad más baja debido a su menor contribución nutricional, y 43 (61,4%) fueron indeterminadas para esta variable.

3.4.4 Lista de prioridades del gobierno

De los 345 RFAA del estudio, encontramos que el 8,7% (n=24) del grupo BGVCOL y el 10% (n=7) del grupo NCB están en la lista de prioridades del gobierno colombiano deben mejorar su productividad y suministro. Representan la etiqueta "incluido" (α=60) con alta prioridad. Los RFAA ausentes de esta lista tienen la etiqueta "no incluido" (α=20) y representan una prioridad baja para esta variable.

3.4.5 Consumo tradicional

De los 345 RFAA del estudio, encontramos que el 36,7% (n=101) del grupo BGVCOL y el 38,6% (n=27) del grupo NCB están presentes en al menos una de las 11 regiones geográficas de Colombia. Los datos faltantes, 63,3% (n=174) en el grupo BGVCOL y 61,4% (n=43) en el grupo NCB representan la categoría indeterminada.

De los 101 conservados en el BGVCOL, 21 se encuentran en una sola región del país, 22 en dos y nueve en tres áreas. Por lo tanto, estos 52 RFAA representan un uso limitado y tienen una prioridad baja. Además, 16 se encuentran en cuatro regiones y 10 en cinco regiones. Estos 26 representan la prioridad media. Por último, siete se encuentran en siete regiones y 16 en ocho regiones. Estos 23 representan un uso amplio con prioridad alta. En comparación, de los 27 con

información del grupo OCN, 10 están en una sola región, cuatro en dos regiones y seis en tres regiones, éstas con baja prioridad, y siete en siete regiones con uso y prioridad alta.

3.5 Limitaciones: Lagunas de información para cultivos autóctonos y parientes silvestres del banco

Incluso con la estrategia de imputación para el grupo BGVCOL, 132 RFAA resultaron en la categoría indeterminada debido a la ausencia de información sobre al menos un pilar que afecta principalmente a Frutas y frutos secos (52%, n=68) y a la región local (43%, n=23 de los Andes y n=34 para Sudamérica Tropical) (Tabla S1). Además, representan muchos parientes silvestres conservados en el BGVCOL. La falta de información por pilares fue 48% (n=132) para el estado de vulnerabilidad, 37,5% (n=103) para la seguridad alimentaria, 30,9% (n=85) para los beneficios económicos y cero para el origen geográfico. En el caso del grupo NCB, no adoptamos una estrategia de imputación para los 70 RFAA. Sin embargo, también encontramos falta de información para cada pilar, así: 55,7% (n=39) para la importancia de la seguridad alimentaria, 38,6% (n=27) para el estado de vulnerabilidad, 25,7% (n=18) para los beneficios económicos, y cero para el origen geográfico.

4 Discusión

Colombia es un país megadiverso que alberga el 10% de la diversidad de la vida en la Tierra, incluidos los recursos fitogenéticos para la alimentación y la agricultura (RFAA) [20]. Aunque se han realizado esfuerzos a nivel mundial para conservar los RFAA, el estudio de la agrobiodiversidad para la mayoría de las especies sigue siendo incipiente en todo el mundo. Los bancos de germoplasma conservan miles de accesiones de varias especies. Sin embargo, son frecuentes las grandes lagunas de falta de conocimiento, incluso para los cultivos de valor económico. Las listas de clasificación utilizando varios criterios ayudan a resaltar lo que debería cuidarse excepcionalmente y considerarse para los estudios de conservación y uso sostenible de los RFAA, incluso con un presupuesto limitado.

Propusimos priorizar los RFAA y aplicar una metodología de lógica difusa al grupo BGVCOL (es decir, 275 conservados en el banco nacional de germoplasma - BGVCOL) y al grupo NCB (es decir, 70 especies externas consideradas por el gobierno como esenciales, pero nunca conservadas en el BGVCOL). Utilizamos un método basado en datos para construir un índice basado en cuatro pilares: origen geográfico, estado de vulnerabilidad, beneficios económicos e importancia para la seguridad alimentaria, dividiendo los grupos de datos BGVCOL y NCB en tres clases: prioridad alta, media y baja. A excepción del origen geográfico, esos pilares deben actualizarse continuamente a medida que evolucionan las tendencias y se dispone de datos más completos sobre especies adicionales.

Aunque esta metodología es específica para Colombia, consideramos que otros bancos de genes nacionales pueden aplicar esta metodología para priorizar especies y orientar actividades. A continuación, explicamos los principales resultados que obtuvimos y la

justificación de cada pilar como marco para construir esfuerzos de investigación y conservación para la clase de alta prioridad. Además, destacamos las limitaciones y los futuros estudios para reforzar este enfoque metodológico en el futuro.

4.1 El origen geográfico y el estatus de vulnerabilidad

Ha habido varios criterios para clasificar y priorizar las especies para los planes de recolección y conservación *ex situ* a largo plazo en todo el mundo dentro de los bancos de germoplasma o bancos de genes [23{25]. El origen geográfico (o el endemismo) y el estado de vulnerabilidad son variables estándar a la hora de priorizar las especies de cultivo para su conservación [23, 24]. La razón principal para elegir estos dos criterios es el concepto de Vavilov de centros de diversidad de cultivos para explicar los patrones de evolución y diversificación de los cultivos alimentarios [49, 50]. Según este concepto, los bancos de genes deberían estar situados justo en las regiones donde evolucionaron los principales RFAA. Ellos tienen un alto grado de variación genética única que no se encuentra en otros territorios [51]. En consecuencia, el objetivo principal de los bancos de germoplasma es rescatar y preservar a largo plazo los RFAA nativos en peligro de extinción debido a la pérdida de hábitat, la erosión genética o la vulnerabilidad a estreses abióticos o bióticos como el calentamiento global, las plagas y las enfermedades [52].

Colombia forma parte del centro de diversidad de varios RFAA andinos y del norte de Sudamérica. Por lo tanto, debería invertir en la investigación y conservación a largo plazo de los RFAA nativos y amenazados como una oportunidad para entender cómo evolucionó esta variación genética, cómo utilizarla para la bioeconomía para mejorar los rasgos de interés para reducir la pobreza y el hambre, y cómo aumentar su resiliencia frente al calentamiento global [53]. De hecho, nuestros datos mostraron que el BGVCOL ya está conservando los RFAA locales, y que éstos representan casi la mitad de la lista. Este patrón sugiere que el BGVCOL, desde su inicio, ha estado conservando RFAA únicos que probablemente están ausentes en otros bancos de germoplasma, haciendo que este banco de germoplasma colombiano sea vital para la conservación *ex situ* de la agrobiodiversidad en el mundo. Sin embargo, detectamos vacíos importantes de información sobre el estatus de vulnerabilidad en ambos conjuntos de datos, BGVCOL y BCN, especialmente para los RFAA locales. Por lo tanto, es posible que falte información crítica en nuestro índice. Los grupos de alimentos de la FAO más afectados fueron los cereales, las frutas y los frutos secos, y las bebidas y las especias.

La ausencia de información básica sobre la biodiversidad es común en regiones tropicales como Colombia y se da en diferentes taxones [54{59]. Por ejemplo, en el caso de las pesquerías marinas de pequeña escala, existen importantes lagunas en la información biológica y ecológica básica [59]. Además, en mamíferos, un grupo conspicuo en el Caribe de Colombia mostró que el 50% de las regiones analizadas no tienen registros desde 1950 [54]. Asimismo, el estudio más actualizado sobre plantas comestibles en Colombia encontró que el 17,6% (n=673) no tiene registros, y el análisis espacial sugiere una insuficiente investigación *in situ* que genera una distribución desigual de registros entre departamentos y bioregiones [29].Las tendencias globales mostraron que los países ricos (es decir, con alto producto interno bruto -PIB- per

cápita) tienen más registros biológicos por kilómetro cuadrado que los países con bajo PIB, aunque estos países ricos no sean los más biodiversos [56].Hay varias razones para explicar esta disparidad. Por ejemplo, la falta de fondos, la falta de infraestructura adecuada, la falta de experiencia para la recolección de datos, las dificultades para acceder a los lugares por razones políticas o de seguridad personal, las dificultades para conseguir publicar los datos o que éstos sean públicos, y una baja proporción de hablantes de inglés [55, 56]. En Colombia, especialmente, los problemas de seguridad y la falta de fondos son transversales a los 60 años de conflicto interno y a la inestabilidad de los organismos gubernamentales encargados de los registros [29, 54, 59]. Afortunadamente, dentro de Colombia, el acuerdo de paz con las FARC firmado en 2016 ha abierto para los científicos nacionales nuevas oportunidades de visitar esos territorios prohibidos a través de expediciones de apoyadas por el Ministerio de Ciencia, Tecnología e innovación de Colombia llamado "Colombia BIO". Estas becas generaron información básica a través de especies y ecosistemas silvestres a través de 20 expediciones entre 2016 y 2018 [60]. De ellas, solo una fue sobre RFAA denominada "la BIO del cacao", que recolectó *Theobroma cacao* y parientes silvestres en regiones de probable origen dentro de Colombia [61].

Basándonos en esta experiencia, animamos a las instituciones de investigación, universidades y organizaciones con proyectos enfocados a cuantificar la biodiversidad silvestre en varios territorios colombianos que integren los RFAA como parte del análisis del paisaje de la biodiversidad silvestre en varios territorios colombianos que integren los RFAA como parte del análisis del paisaje. Varias metodologías en la investigación de la biodiversidad combinan la cobertura y las incertidumbres de la información para generar indicadores que permitan priorizar vacíos y acciones significativas [57, 62, 63]. En Colombia, el enfoque metodológico que relaciona la cantidad y la calidad de la información para generar un puntaje de urgencia de investigación en 271 especies de mamíferos colombianos de la región del Caribe tiene el potencial de ser adaptado para varios RFAA [54]. Además, para el análisis de los RFAA, la perspectiva etnobotánica y las ciencias participativas son extremadamente importantes para encontrar los vacíos y las prioridades del inventario de biodiversidad [29, 63-65]. Una pregunta importante es qué RFAA están en uso y si la comunidad rural o los mercados campesinos han notado algunos cambios en la presencia/ausencia de estos RFAA durante las últimas décadas. Como muchos países latinoamericanos, Colombia tiene varios problemas imbricados asociados con la agricultura de los pequeños agricultores y los mercados campesinos, además de un conflicto interno de larga duración. Entre ellos, la apertura económica de la década de 1990 y las fluctuaciones del mercado nacional e internacional [66]. Por lo tanto, un esfuerzo de investigación combinado desde una perspectiva multidisciplinar y trans-disciplinar que combine los conocimientos locales y científicos podría completar la información esencial que falta a lo largo del tiempo y es también una oportunidad para diseñar estrategias de inversión para conservar la biodiversidad local con una perspectiva de desarrollo sostenible [59]. De hecho, la información que falta también representa una oportunidad para diseñar iniciativas en las que el esfuerzo *ex situ* de BGVCOL interactúe con las comunidades que realizan la conservación *in situ*/en la granja y el seguimiento del estado de los RFAA a lo largo del tiempo [67].

4.2 Los beneficios económicos y la importancia de la seguridad alimentaria

En contraste con el origen geográfico y el estatus de vulnerabilidad, por lo que sabemos, los beneficios económicos y la importancia para la seguridad alimentaria no son variables estándar que se utilicen para clasificar los RFAA y tomar decisiones de inversión sobre la investigación de conservación *ex situ*. Las elegimos porque son muy informativas hoy en día para atender a nuestras particulares circunstancias socioeconómicas de pobreza y hambre frente a la agrodiversidad insostenible. Por lo tanto, ambos pilares nos ayudaron a poner este problema en la misión del banco nacional de germoplasma. Colombia reportó que, en 2021, el 39,3% de la población nacional sufría de pobreza monetaria, pero el promedio es peor para la población rural con un 44,6% [28]. Además, el informe de la FAO-PMA de febrero a mayo [68] alertó que la inseguridad alimentaria en Colombia podría deteriorarse en los meses siguientes por varios factores como la inestabilidad política, las altas tasas de inflación, la crisis migratoria regional, los retrasos en la implementación del acuerdo de paz de 2016 y la pandemia del COVID-19. Así, en diciembre de 2021, 7,3 millones de colombianos se encontraban en inseguridad alimentaria o necesitaban asistencia alimentaria [68]. Estos informes refuerzan la importancia de alinear la misión de la BGVCOL para atender las necesidades urgentes del 22,9% de la población rural [69].

Nuestro índice encontró 54 RFAA del grupo BGVCOL de 15 grupos taxonómicos y 11 del grupo NGB altamente priorizados por sus beneficios económicos. Ellos tienen un alto rendimiento, ingresos y perspectivas de exportación, y cubren la mayor parte del país. Por lo tanto, tienen el potencial de reducir la pobreza de las familias rurales y familias agrícolas. En 2020, la agricultura aportó el 7,6% del PIB [70]. Por lo tanto, la inversión en conservación e investigación en estos RFAA de rango altamente prioritario podría aumentar los ingresos de las familias rurales y ofrecer una oportunidad directa para conectar la desmovilización de los combatientes en proyectos productivos rurales [71]. Además, al centrar la inversión en los RFAA de mayor rango para beneficios económicos en las áreas con mayor retorno de conservación, también podría facilitar la prevención de la conversión de bosques por actividades agrícolas que actualmente están acelerando la pérdida de biodiversidad dentro de Colombia [72]. Asimismo, nuestro índice encontró 36 RFAA de 10 grupos taxonómicos conservados en el BGVCOL como altamente prioritarios para la seguridad alimentaria, pero ninguno dentro del grupo de los NCB resultó de alta prioridad. Estos representan alimentos nutritivos y asequibles que forman parte de nuestras tradiciones culturales y culinarias [73]. La mayoría forma parte de las especies NUS (Neglected and underutilized species), con muy pocos estudios etnobotánicos para promover su uso en el País [74, 75]. Por lo tanto, representan una oportunidad para ser fomentada entre los investigadores, los fitomejoradores y las partes interesadas como parte de los cultivos que podrían ayudar con la meta de los ODS de reducir la pobreza y el hambre.

Desafortunadamente, los hogares rurales de Colombia han sustituido progresivamente estos NUS de forma exponencial desde la revolución verde debido a la imposición de un enfoque tecnocrático del desarrollo rural [76]. La consecuencia ha sido el desuso de muchos NUS, incluidos los conocimientos culinarios la pérdida de autonomía y el aumento de la pobreza de determinadas comunidades y territorios [29]. Por lo tanto, el estudio de los RFAA identificados

con alto potencial para resolver la seguridad alimentaria necesita seguir reforzando los estudios biológicos básicos con perspectivas etnográficas y sociológicas para salvaguardar este legado de agrobiodiversidad [29, 76]. Los pilares de los beneficios económicos y la seguridad alimentaria fueron incluidos en el índice para establecer un entendimiento con los responsables de la toma de decisiones. Sin embargo, es importante señalar que el BGVCOL conserva una amplia gama de cultivos, incluidos cultivos rentables, plantas silvestres, NUS y parientes silvestres de cultivos, que son cruciales para garantizar la seguridad alimentaria. El índice integra cuatro pilares independientes, y propusimos una estrategia de imputación para mitigar los efectos de las lagunas de información y evitar el sesgo hacia los cultivos más rentables.

4.3 Acciones presentes y futuras para los RFAA que resultaron de alta prioridad

Aplicamos la metodología del índice a 345 RFAA de dos conjuntos, el BGVCOL y el BCN. Nuestra metodología priorizó 25 especies, 24 del BGVCOL (papa, tomate, tomate de árbol, piña, cacao, papaya y yacón) y una del BCN (café). Dentro del BGVCOL priorizado, identificamos dos grupos. El primero comprende grupos taxonómicos dentro del género *Solanum* (es decir, la papa, el tomate y el tomate de árbol) y el cacao. Este grupo tiene más de 200 accesiones conservadas por especie, incluyendo parientes silvestres. En cambio, el segundo grupo comprende los cultivos NUS, la piña, la papaya y el yacón. Tienen pocas accesiones dentro del BGVCOL, sólo una especie representativa dentro de dentro de cada taxón, y ningún pariente silvestre conservado.

Debido a las diferencias significativas entre los dos grupos, sugerimos diferentes acciones futuras de investigación. En el caso del primer grupo, sugerimos continuar apoyando el análisis de la diversidad fenotípica y genómica como base para el fortalecimiento de los programas de mejoramiento genético en Colombia. A continuación, se detallan los resultados de investigación asociados a estas especies y las acciones futuras de investigación.

En el caso de la colección de papa conservada en el BGVCOL, 809 accesiones de papa del grupo andigenum (diploides y tetraploides) cuentan con una caracterización genómica. El análisis demostró una colección de germoplasma muy diversa a nivel fenotípico y genómico [77]. Esta diversidad es prometedora para los programas de mejoramiento. Actualmente, estamos completando la caracterización molecular de la mayoría de las accesiones de razas autóctonas, explorando los datos fenotípicos históricos para asociar rasgos, y definiendo una colección central. Las futuras actividades de incluyen el uso de accesiones específicas para programas de mejoramiento para encontrar resistencia a determinadas enfermedades.

La colección de cacao conservada en el BGVCOL cuenta con 565 accesiones caracterizadas a nivel genómico. El análisis demostró una alta diversidad genética entre los grupos, apoyando que algunos grupos genéticos se originaron en Colombia que probablemente es un centro de origen para este cultivo [78]. Además, esta colección tiene estudios de asociación que apoyan un programa de mejoramiento [79, 80]. Asimismo, investigadores de Agrosavia realizaron dos expediciones en el alto Amazonas y el Pacífico, en busca de parientes silvestres del cacao. Las futuras acciones de investigación están asociadas con la caracterización fenotípica y genotípica

de los parientes silvestres y con el fortalecimiento de la investigación y conservación de parientes silvestres [81].

En los casos del tomate y del tomate de árbol, debemos seguir el mismo camino mencionado anteriormente. Sin embargo, están atrasados en los objetivos de investigación. Los esfuerzos para de caracterización de las accesiones por rasgos morfológicos tienen al menos treinta años, y no existe una caracterización molecular de estas colecciones conservadas en el BGVCOL. Por lo tanto, el estatus de alta prioridad para esas colecciones de especies es una oportunidad para solicitar financiación asociada a la caracterización fenotípica y genómica. Afortunadamente, los esfuerzos mundiales para caracterizar la genética de los tomates, incluyendo 100 genomas [82] y el pan-genoma [83], facilitarán este camino.

En contraste con el primer grupo de RFAA de alta prioridad, los futuros objetivos de investigación y los fondos son bastante diferentes para el segundo grupo de prioritarios que corresponden a NUS. La papaya, la piña y el yacón tienen 110, 72 y 2 accesiones, respectivamente, conservadas dentro del BGVCOL. Incluso dentro del BGVCOL, estos NUS están subrepresentados con base al número de accesiones. Además, no se conservan parientes silvestres. En estos casos, sugerimos reactivar su investigación diseñando un trabajo de campo para identificar y recoger muestras en todo el país. Animamos a los diferentes centros de investigación e instituciones académicas (nacionales o internacionales) a trabajar en colaboración para determinar la distribución de los cultivos y parientes silvestres en el país para planificar expediciones y proteger estos recursos genéticos.

Por último, los resultados mostraron una especie prioritaria de café del grupo NCB (70 RFAA nunca conservados en el BGVCOL). Afortunadamente, el CENICAFE (www.cenicafe.org) (una institución de investigación colombiana centrada en el café) conserva 1031 accesiones de 10 especies, con una amplia representación de *Coffea arabica* (85%). En CENICAFE, el banco caracteriza la colección para apoyar el programa de mejoramiento [84]. No obstante, queremos destacar la importancia de un plan nacional de investigación de especies por sus beneficios económicos para el país y su potencial de vulnerabilidad frente al calentamiento global [85, 86].

4.4 Futuras iniciativas de investigación para mejorar el índice

Este estudio representa el primer intento de alinear la misión de conservación *ex situ* del banco de germoplasma vegetal colombiano con los ODS y determinar cómo asignar el presupuesto limitado para los Recursos Fitogenéticos para la Alimentación y la Agricultura (RFAA) en un país megadiverso. Seleccionamos variables y pilares que tienen un único dato para cada 345 RFAA y los clasificamos con éxito en prioridad alta, media o baja utilizando la lógica difusa. Sin embargo, también identificamos varias oportunidades para mejorar este esfuerzo metodológico en el futuro. Estas son: (1) añadir otras especies externas no consideradas en los 70 RFAA del grupo NCB. (2) para algunas variables, en lugar de tener un único dato por RFAA, incluir la diversidad entre accesiones en el análisis. (3) incluir un nuevo pilar asociado a las

acciones climáticas (13º ODS) y (4) estimar variables para el coste y la gestión de la conservación *ex situ* a través de colecciones.

Una de las perspectivas más interesantes para mejorar el índice es ampliar el enfoque en otros RFAA del grupo NCB. El estudio actual se centró en 70 RFAA externos que aparecen en varias listas gubernamentales. Sin embargo, ninguna de las especies del grupo NCB tuvo una puntuación alta para resolver la seguridad alimentaria, y sólo una resultó prioritaria para los beneficios económicos (el café). Nuestra hipótesis es que los 70 RFAA de las listas gubernamentales que se ajustan al grupo NCB están sesgados hacia los RFAA externos y no vulnerables, que no son necesariamente las mejores opciones para reducir el hambre y la pobreza. Por lo tanto, es imperativo actualizar las listas gubernamentales e identificar los parientes silvestres de RFAA ya conservados en el BGVCOL. Afortunadamente, por lo que sabemos tres estudios recientes están centrando la atención en aquellos RFAA locales e infrautilizados y parientes silvestres no considerados antes. No es de extrañar que estos estudios desvelen una asombrosa biodiversidad vegetal autóctona que merece un análisis futuro por su potencial para obtener beneficios económicos y seguridad alimentaria nacional [29, 64, 87]. Además, estos estudios sugieren que esta lista actualizada de RFAA externos supera con creces los estudios anteriores que sólo reportaron de entre 50 y 167 RFAA, así como la importancia de seguir explorando una perspectiva etnobotánica [29, 87-89]. El primer estudio se centró en el mercado de hierbas y aromáticas de Bogotá en la medicina tradicional y los rituales. El análisis transdisciplinario encontró 391 especies, 201 de ellas nativas. El estudio priorizó 80 especies entrevistando a agricultores, recolectores y vendedores, y registró el estado de vulnerabilidad, la mejor conservación, recetas y usos para la industria alimentaria y farmacéutica [64]. El segundo estudio se centró en los frutos silvestres comestibles de Colombia con un alto potencial de beneficios económicos y de seguridad alimentaria. Combinando la revisión de la literatura con bases de datos de herbario, encontraron 706 especies, 45 endémicas de Colombia. Además, 613 son exclusivamente silvestres, y 90 son silvestres o cultivadas [87]. Por último, el estudio más reciente de plantas comestibles con una perspectiva biogeográfica encontró 3805 especies, 662 cultivadas y 158 nativas [29].

Otro tema para mejorar el índice es incluir en el análisis la diversidad entre accesiones en lugar de tener un único dato por RFAA para determinadas variables. Por ejemplo, la caracterización de los RFAA conservados en varios bancos de germoplasma ha demostrado que en la variable de contribución nutricional (pilar de la seguridad alimentaria), existe una amplia variación [90, 91]. Este proceso de fenotipado es costoso para todos los bancos de germoplasma, independientemente del presupuesto disponible, ya que requiere cultivar todas las accesiones en campo o un grupo seleccionado que represente toda la diversidad genética (es decir, colección núcleo o colección mini-núcleo) [92]. Así, comenzando con aquellos RFAA que resultaron de alta prioridad en este estudio, se podrían diseñar proyectos que permitieran caracterizar aquellos rasgos informativos de interés agronómico entre accesión y, eventualmente, comenzar a incluir esta información para un futuro índice mejorado.

Además, podríamos incluir nuevas variables o pilares, de los que es obligatorio tener datos por accesión, como es el caso de la acción por el clima, el 13º ODS. Este objetivo pretende "integrar las medidas relativas al cambio climático en las políticas, estrategias y planificación nacionales" [90]. Nuestra versión actual del índice no contiene ningún pilar asociado a la medición de la vulnerabilidad o la capacidad de adaptación de los RFAA ante el cambio climático. Este estudio se centró en Colombia como una sola unidad (véase el pilar de origen geográfico) o como divisiones culturales gruesas (ver la variable de regiones culturales dentro del pilar de seguridad alimentaria) para estructurar el índice de los cuatro pilares. Además, utilizamos un solo dato para cada variable por cada RFAA. Por lo tanto, estaba fuera del objetivo principal detallar la distribución espacial geográfica de cada acceso a los RFAA y dividir a Colombia en regiones o departamentos para modelar el efecto o la capacidad de adaptación de cada uno de los 375 RFAA en los escenarios de cambio climático. Sin embargo, esto es sin duda un estudio futuro a corto plazo. El principal problema en el diseño de este análisis es la ausencia de datos espaciales a nivel de accesión, sobre todo en el caso de los RFAA sin pasaportes que omiten la información espacial dentro del BGVCOL y los externos. Sin embargo, una posible solución es utilizar el Departamento Nacional de Estadística (DANE) para determinar la distribución de los cultivos [91]. Además, se podría combinar el enfoque de diversidad filogenética espacial y el análisis de brechas ya utilizado para algunos cultivos y parientes silvestres, con el fin de identificar los RFAA prioritarios para la conservación *ex situ* y los refugios climáticos globales para los RFAA ya conservados en el BGVCOL y externos [92, 93]. Una vez que completemos este análisis e identifiquemos su potencial y sus limitaciones, podemos crear un índice para clasificar los RFAA para vulnerabilidad o adaptación al cambio climático [94]. A continuación, podemos unir este pilar del cambio climático con el actual índice de cuatro pilares para tomar decisiones informadas sobre la inversión en investigación de los RFAA priorizados y el diseño de la conservación in situ/en la granja con las comunidades en las zonas de refugio identificadas [92, 93].

Por último, esperamos que el índice se asocie con las decisiones de gestión interna dentro del BGVCOL relacionadas con el coste de la conservación, la regeneración y a la actualización de la documentación, incluida la base de datos de libre acceso construida en este estudio. A pesar de la importancia de este análisis económico, todavía no tenemos análisis asociados al coste-beneficio de la conservación *ex situ* en el país. Hay algunas pistas de los cálculos de otros bancos de germoplasma. Estos análisis anteriores incluían todas las etapas de gestión, el proceso de la introducción (es decir, la regeneración, las pruebas de viabilidad y salud), el de almacenamiento a largo plazo, el coste de la regeneración y las pruebas de viabilidad que se realizan periódicamente (cada 20-30 años), y el coste de distribución. Así, en 11 bancos de germoplasma del sistema CGIAR, el coste de almacenamiento por año para la mayoría de las especies es de 1,50 dólares. Sin embargo, para las especies con polinización cruzada, como el maíz, es de 2,16 dólares, y para la conservación *in vitro*, como la yuca, es de 11,98 dólares. Además, para las especies que requieren una regeneración repetida, el coste aumenta.
Por ejemplo, el coste por accesión/año de los forrajes es de 89,35 dólares, el del arroz silvestre es de 68,76 dólares, los garbanzos 15,48 dólares y el sorgo 14,66 dólares [95]. Debido a que el coste de la conservación *ex situ* cambia en función de la biología de los RFAA, otro criterio fundamental para el banco de germoplasma colombiano, que actualmente de 275 RFAA debe

ser el costo de la conservación *ex situ* a largo plazo, basado en la periodicidad de la regeneración y la periodicidad de las pruebas de regeneración y viabilidad [2, 95]. Por lo tanto, una clasificación de los RFAA basado en el pilar del coste de conservación podría ser beneficioso. En primer lugar, podría convencer a las partes interesadas de que la inversión en el banco de germoplasma nacional es un seguro para el país, como una acción responsable para el futuro [2, 13]. En segundo lugar, estas evaluaciones económicas pueden ayudar a comprender otras metodologías rentables de conservación a largo plazo de determinados RFAA (por ejemplo, la crioconservación en vez de la conservación en el campo) [96, 97]. Por último, ayudaría a situar la conservación *ex situ* y la investigación de los bancos de germoplasma nacionales como una inversión asociada a los fondos en bioeconomía que actualmente promueven los objetivos de investigación del Ministerio de Ciencia [98, 99].

5 Conclusiones

El principal problema de la investigación en conservación *ex situ* de los RFAA es la falta de financiación. En el caso de países megadiversos como Colombia, la decisión de cómo invertir el dinero es aún más crítica. Por lo tanto, el desarrollo de una herramienta para decidir qué RFAA es prioritario para la inversión en investigación y la conservación, en consonancia con la solución de los usos sostenibles y de los problemas sociales es imperativo. Aquí desarrollamos por primera vez un índice basado en datos de cuatro pilares de información para clasificar 345 RFAA colombianos alineados con los objetivos de desarrollo sostenible tales como cero hambres y pobreza cero. Estos incluyen el origen geográfico, estado de vulnerabilidad, beneficios económicos e importancia para la seguridad alimentaria. El índice de cuatro pilares utilizó la lógica difusa y clasificó con éxito cada RFAA en tres grupos: alta, media y baja prioridad. El índice encontró 25 RFAA de alta prioridad. De ellos, 24 son ya colecciones *ex situ* del banco de germoplasma de Colombia (BGVCOL), y uno es externo (es decir, no se conservaba actualmente en el BGVCOL). Nuestra metodología demostró varias ventajas: los datos utilizados para construir el índice vienen de bases de datos de libre acceso que también resumimos en una única base de datos de libre acceso. Además, utilizamos un enfoque basado en los datos e independiente de las preferencias y de los científicos del banco, en consonancia con dos objetivos de desarrollo sostenibles. El enfoque descrito se alineó con dos de las cuatro misiones definidas recientemente por el gobierno colombiano para abordar los mayores retos del país: (1) Bioeconomía, ecosistemas naturales y territorios sostenibles, y (2) Derecho a la Alimentación [103].

Asimismo, la clasificación de las especies en tres categorías prioritarias simplificó la información para las partes interesadas no científicas y los políticos que suelen decidir cómo invertir los fondos de investigación. Además, la metodología identificó las lagunas de información más importantes sobre los RFAA autóctonos y los parientes silvestres y las posibilidades de imputar información cuando se dispone de información de los RFAA más cercanos. Por último, el índice es versátil, ya sea para añadir en el futuro más RFAA (especialmente para aquellos no conservados actualmente en el BGVCOL) o para añadir más pilares asociados con otros objetivos de sostenibilidad, como la vulnerabilidad o la adaptación al cambio climático. Estas

ventajas hacen de este índice una herramienta de decisión flexible para aplicar en otros bancos de germoplasma nacionales que carecen de financiación, pero están interesados en identificar los RFAA clave para alinear la misión de conservación *ex situ* con los objetivos sostenibles. Reconociendo que muchos de los problemas anteriores se dan en América Latina, este estudio también está disponible en español para promover una discusión sobre esta herramienta a través de los bancos de germoplasma regionales (Archivo S1).

6 Disponibilidad de los datos

Hemos construido una interfaz web para dar acceso a los datos presentados aquí. Los conjuntos de datos utilizados durante el presente estudio están disponibles temporalmente en el repositorio http://indicebgv.agrosavia.co:3997/ . El conjunto de datos y los scripts utilizados para el índice de prioridad en R están disponibles en https://github.com/phrh/BGVCOLPriorityIndex.

7 Financiación

Este estudio hizo parte del proyecto BGA Documentación VG vegetal con fondos de la Corporación Colombiana de Investigación Agropecuaria - AGROSAVIA, financiado con recursos públicos del Ministerio de Agricultura y Desarrollo Rural (MADR).

8 Contribuciones de los autores

IC-S y PHR-H concibieron y supervisaron el estudio. DAD-D desarrolló los scripts en R y el repositorio web. SMP-M y ZXS-N recuperaron la información de las bases de datos de libre acceso. IC-S y PHR-H redactaron la primera versión de este manuscrito. Todos los autores analizaron los datos, editaron el manuscrito y aprobaron la versión presentada.

9 Agradecimientos

Un agradecimiento especial a C. Medina, A. Caicedo, A. Henríquez, J. Aguirre, R. Torres y R. Garzón por ayudarnos en el inicio de este proyecto con la lista de especies del inventario BGVCOL.

10 Figuras

Figura 1. Diagrama circular que representa los 275 recursos fitogenéticos para la alimentación y la agricultura (RFAA) conservados actualmente en el Banco Nacional de Germoplasma Vegetal

(es decir, grupo BGVCOL). Cada círculo representa una especie de cultivo con su nombre común, y el tamaño del radio está en función del número de accesiones conservadas.

Figura 2. (A) La estrategia general para analizar los datos brutos de 345 Recursos Fitogenéticos para la Alimentación y Agricultura (RFAA) de Colombia. Doscientos setenta y cinco (275) forman parte del Banco Nacional de Germoplasma Vegetal (es decir, el grupo BGVCOL), y 70 son esenciales para el Gobierno de Colombia, pero no se conservan actualmente en el BGVCOL (es decir, el grupo BCN). (i) El análisis incluye cuatro pilares de datos con diferentes variables (el número en paréntesis). (ii) El pre procesamiento de los datos brutos para cada variable dentro de cada pilar: origen geográfico origen geográfico (verde), vulnerabilidad (amarillo), importancia económica (rojo) e importancia para la seguridad alimentaria (azul). Tanto la importancia económica como la importancia para la seguridad alimentaria tenían varios huecos de información para algunos RFAA. En esos casos, el pre procesamiento incluyó valores de los En estos casos, el pre procesamiento incluyó los valores del RFAA filogenéticamente más cercano para imputar y, por tanto, el cálculo de la incertidumbre (recuadro púrpura). (iii) La construcción de la función de pertenencia de las variables (conjuntos difusos) se basa en el singleton o en el trapezoide. El origen geográfico y la vulnerabilidad suponen una única variable cualitativa que generó una función lógica difusa singleton. La importancia económica y la seguridad alimentaria tuvieron cuatro y diez variables cuantitativas, que generaron funciones de lógica difusa trapezoidales. (iv) La inferencia final para la lista de priorización y el nivel de incertidumbre se basa en funciones lógicas difusas gaussianas y de un solo dígito. (B) El detalle de la estrategia de imputación con las rutas y los resultados (recuadro púrpura en A). En función de la ruta de imputación tomada, la información recibe una etiqueta para rastrear el nivel de incertidumbre (es decir, fiable, incertidumbre GP o incertidumbre PCG). (C) Un ejemplo de las funciones de pertenencia (conjuntos difusos) utilizadas en los pasos iii y iv de A. El singleton (para datos categóricos) utilizó a=50, y SD=0.001. El trapezoide (para datos numéricos) utilizó 25, 50, 75 y 100 como límites de la forma en la parte inferior izquierda a, superior izquierda m, superior derecha n, e inferior derecha b, respectivamente. Al final del proceso la función de pertenencia gaussiana (para inferir el interés del RFAA) utilizó μ=50 y σ=5. Las tres funciones tienen un grado de pertenencia igual a uno.

Figura 3. La clasificación final de 345 recursos fitogenéticos para la alimentación y la agricultura (RFAA) en diferentes categorías dentro de los cuatro pilares muestra el número y el porcentaje de edad. (A) los 255 RFAA conservados en el Banco Nacional de Germoplasma Vegetal tras la imputación (grupo BGVCOL) y (B) los 70 RFAA no conservados actualmente en el BGVCOL (grupo NCB). El pilar del origen geográfico tuvo tres categorías: Local (verde), Cercano (amarillo) y Distante (rojo). El pilar de la vulnerabilidad tenía tres categorías: Amenazada (verde), menor preocupación (amarillo) y no evaluado (gris). El pilar de la importancia económica tuvo cuatro categorías: Alta (verde), media (amarillo), baja (rojo) e indeterminada (gris). La importancia de la seguridad importancia de la seguridad alimentaria tenía cuatro categorías: Alta (verde), media (amarillo), baja (rojo) e indeterminada (gris). Por último, el índice de prioridad que combinaba los cuatro pilares tuvo tres categorías: Alta (verde), media (amarillo) y baja (rojo).

Figura 4. El resultado del índice de prioridad para los 275 recursos fitogenéticos para la alimentación y la agricultura (RFAA) conservados en el Banco Nacional de Germoplasma Vegetal (es decir, el grupo BGVCOL). Las columnas representan las categorías de la FAO para las especies alimenticias. Las filas representan los tres niveles de prioridad para la inversión en investigación; así: 24 en prioridad alta (verde), 72 en prioridad media (amarillo) y 179 en prioridad baja (rojo). Los iconos representan cada grupo de taxones, y el número debajo del icono representa el número de especies de cada taxón (las referencias de los íconos utilizados se encuentran en la tabla S2).

11 Tablas

Tabla 1. Las variables evaluadas en este estudio consideran cuatro pilares de información: Origen geográfico, vulnerabilidad, importancia económica e importancia para la seguridad alimentaria. Cada variable dentro del pilar especifica el tipo de datos (singleton para datos categóricos y trapezoidal o gaussiano para datos continuos), el número de clases para cada tipo (conjuntos difusos), el nombre de cada clase con su prioridad entre paréntesis, la función lógica difusa y los parámetros para cada función de pertenencia. La clase indeterminada significa datos no disponibles o inciertos.

Tabla 2. Lista de grupos de cultivos y el número de especies sin información imputada utilizando una especie dentro del Banco Nacional de Germoplasma Vegetal (grupo BGVCOL) para los pilares económico y de seguridad alimentaria. Había dos opciones posibles de imputación. La primera opción fue el pool genético, que se utilizaba cuando había una especie en la lista de estudio que representaba el cultivo representativo más cercano o la especie más cercana del mismo género. En ausencia de la especie o el género más cercanos, la opción fue el grupo de cultivos provisional. Estas son las especies que comparten la misma categoría alimentaria definida por la FAO. Para las especies no conservadas actualmente en el BGVCOL (grupo NCB), no imputamos ninguna especie.

Tabla 3. El número de RFAA conservados en el Banco Nacional de Germoplasma Vegetal (grupo BGVCOL) separándolos por el nivel de prioridad para los pilares de importancia económica e importancia para la seguridad alimentaria antes y después del proceso de imputación para las especies sin información en todas las bases de datos consultadas (en la categoría indeterminada). La imputación redistribuyó el recuento final para cada categoría. Aun así, el 31% y el 37% de los RFAA de cada pilar no tuvieron un grupo genético o un grupo de cultivo provisional que pudiera utilizarse para la imputación (véase la Tabla 2).

Tabla 4. La media y la desviación estándar de cada variable (ingresos, índice de Lafay, cobertura del municipio y rendimiento) dentro del pilar de beneficios económicos para las nueve categorías de alimentos de la FAO fueron calculadas para las especies conservadas en el BGVCOL y el NCB. La abreviatura "NA" se utiliza cuando no se dispone de información.

Tabla 5. Para el pilar de Seguridad Alimentaria, se calcularon la media y la desviación estándar de la variable de asequibilidad para cada una de las nueve categorías de alimentos de la FAO para las especies conservadas en BGVCOL y NCB. Si no se disponía de información, se utilizó "NA" como abreviatura.

Tabla 6. La variable contribución nutricional fue analizada para cada una de las nueve categorías de alimentos de la FAO para las especies conservadas en BGVCOL y OCN en el pilar de Seguridad Alimentaria, calculándose la media y la desviación estándar. En los casos en que la información no estaba disponible, se utilizó "NA" como abreviatura.

12 Información complementaria

12.1 Lista de tablas complementarios

Tabla S1. Lista de recursos fitogenéticos para la alimentación y la agricultura (RFAA) del grupo BGVCOL sin datos (es decir, categoría de etiqueta indeterminada) tras la estrategia de imputación. Cada RFAA muestra el nombre científico, el origen geográfico y la clasificación de la FAO.

Tabla S2. Lista de las referencias de los iconos utilizados en la Figura 4.

12.2 Lista de figuras suplementarias

Figura S1. La lista priorizada de recursos fitogenéticos para la alimentación y la agricultura (RFAA) y productos animales. El gobierno colombiano desarrolló esta lista para garantizar sus políticas de producción y abastecimiento para mejorar el consumo estable en la dieta de la población colombiana. Un icono único representa cada especie o producto, mostrando la separación por la clasificación de la FAO de los grupos de alimentos.

Figura S2. El mapa de Colombia mostrando 11 regiones geográficas con diferentes niveles de gris. Dentro de cada región hay un pastel con el número de Recursos Fitogenéticos para la Alimentación y la Agricultura (RFAA) esenciales para la tradición alimentaria de cada región, separándolos por diferentes colores que representan ocho grupos de alimentos de la FAO. El tamaño de la tarta equivale al número de RFAA incluidos.

Figura S3. Origen geográfico de los 345 recursos fitogenéticos para la alimentación y la agricultura (RFAA) en 26 regiones del mundo. (A) Doscientos setenta y cinco conservados en el Banco Nacional de Germoplasma Vegetal (grupo BGVCOL), (B) 70 no se conservan actualmente en el BGVCOL (grupo NCB). La barra muestra el nombre de la región, indicando en azul el número de RFAA con origen en cada región. El color de la barra representa las tres categorías de priorización utilizadas en este estudio. En verde, el origen local con prioridad alta incluye Sudamérica Tropical y los Andes, dos regiones donde se localiza Colombia. El origen cercano

con prioridad media incluye a Centroamérica, México y el Caribe en amarillo. Finalmente, en rojo, las regiones lejanas con prioridad baja.

Figura S4. Clasificación de 275 RFAA conservados en el Banco Nacional de Germoplasma Vegetal (BGVCOL) según su estado de vulnerabilidad. (A) El porcentaje de estado de vulnerabilidad de los RFAA analizados por categorías alimentarias de la FAO: No evaluado (gris), Preocupación menor (amarillo) y Amenazado (naranja). (B) El número de RFAA sin información sobre la vulnerabilidad por regiones geográficas en el mundo.

Figura S5. La clasificación de 70 RFAA no conservados en el Banco Nacional de Germoplasma Vegetal (es decir, el grupo NCB) por su estado de vulnerabilidad. (A) El porcentaje del estado de vulnerabilidad de los RFAA analizados por categorías de alimentos de la FAO: No evaluado (gris), Preocupación menor (amarillo) y Amenazado (naranja). (B) El número de RFAA sin información sobre la vulnerabilidad por regiones geográficas en el mundo.

Figura S6. Boxplots mostrando la mediana y la variación de cuatro variables consideradas en el pilar de beneficios económicos para los RFAA conservados en el Banco Nacional de Germoplasma Vegetal (BGVCOL) separados por categorías de alimentos de la FAO con el número de especies dentro de cada categoría (n). (A) Ingresos (miles de USD ha^{-1}). (B) Índice de Lafay. (C) Cobertura municipal (%). (D) Rendimiento (t ha^{-1}). Un USD = 3.418 COP promedio anual en los últimos cinco años.

Figura S7. Boxplots mostrando la mediana y la variación de cuatro variables consideradas en el pilar de beneficios económicos para los RFAA no conservados en el Banco Nacional de Germoplasma Vegetal (grupo BCN) separados por categorías de alimentos de la FAO con el número de especies dentro de cada categoría (n). (A) Ingresos (miles de USD ha^{-1}). (B) Índice de Lafay. (C) Cobertura municipal (%). (D) Rendimiento (t ha^{-1}). Un USD = 3.418 COP promedio anual en los últimos cinco años.

Figura S8. Boxplots mostrando la mediana y la variación de la asequibilidad en USD por 100 g de porción comestible para (A) Calcio-Ca, (B) Hierro-Fe, (C) Zinc-Zn, y (D) Energía separando los RFAA conservados en el Banco Nacional de Germoplasma Vegetal (BGVCOL) por categorías de alimentos de la FAO indica el número de especies (n). Un USD = 3.418 COP promedio anual en los últimos cinco años.

Figura S9. Boxplots mostrando la mediana y la variación de la asequibilidad en USD por 100 g de porción comestible para (A) Calcio-Ca,(B) Hierro-Fe, (C) Zinc-Zn, y (D) Energía separando los RFAA no conservados actualmente en el Banco Nacional de Germoplasma Vegetal (grupo NCB) por categorías de alimentos de la FAO indica el número de especies (n). Un USD = 3.418 COP promedio anual en los últimos cinco años.

Figura S10. Boxplots mostrando la mediana y la variación del porcentaje (%) de la meta nutricional diaria para (A) Calcio-Ca,(B) Hierro-Fe, (C) Zinc-Zn, y (D) Energía, separando los RFAA

conservados en el Banco Nacional de Germoplasma Vegetal (BGVCOL) por categorías de alimentos de la FAO indicando el número de especies (n).

Figura S11. Boxplots mostrando la mediana y la variación del porcentaje (\%) del objetivo nutricional diario para (A) Calcio-Ca, (B) Hierro-Fe, (C) Zinc-Zn, y (D) Energía separando los RFAA no conservados en el Banco Nacional de Germoplasma Vegetal (es decir, el grupo NCB) por categorías de alimentos de la FAO indicando el número de especies (n).

Figura S12. Los RFAA conservados en el BGVCOL que resultaron en la categoría de alta prioridad para (A) el pilar de origen geográfico y (B) el pilar de vulnerabilidad. Los RFAA están agrupados por categorías alimentarias de la FAO, indicando el nombre y el número de especies. Los iconos representan el taxón del cultivo y son los mismos utilizados en la Fig. 4.

Figura S13. Los RFAA conservados en el BGVCOL que resultaron en la categoría de alta prioridad para (A) el pilar de beneficio económico y (B) el pilar de seguridad alimentaria. Los RFAA están agrupados por categorías alimentarias de la FAO, indicando el nombre y el número de especies. Los iconos representan el taxón del cultivo.

Figura S14. Los RFAA conservados en el Banco Nacional de Germoplasma Vegetal (BGVCOL) dentro de la clase de mayor prioridad para dos pilares: (A) El pilar de beneficio económico con cuatro variables, índice de Lafay, rendimiento, cobertura municipal e ingresos. (B) El pilar de seguridad alimentaria con cuatro variables, lista de prioridades del gobierno, consumo tradicional, contribución nutricional y asequibilidad de nutrientes. Los RFAA están divididos por categorías alimentarias de la FAO en un icono único, indicando el nombre común y el número de especies (n), ordenadas de mayor a menor puntuación media (AS) medida como porcentaje. El color muestra la AS obtenida para cada variable va de 0 (azul) a 100 (rojo). Los iconos son los mismos que los utilizados en la Fig. 4.

12.3 Lista de archivos suplementarios

Archivo S1. El manuscrito en español.
